# Visible-light-mediated flow protocol for Achmatowicz rearrangement

**DOI:** 10.3762/bjoc.20.213

**Published:** 2024-10-08

**Authors:** Joachyutharayalu Oja, Sanjeev Kumar, Srihari Pabbaraja

**Affiliations:** 1 Department of Organic Synthesis & Process Chemistry, CSIR-Indian Institute of Chemical Technology, Hyderabad-500007, India; 2 Academy of Scientific and Innovative Research (AcSIR), Ghaziabad 201002, India

**Keywords:** Achmatowicz reaction, flow chemistry, furfuryl alcohols, photocatalyst, sunlight

## Abstract

The batch processes of APIs/pharmaceutical synthesis are prone to suffer significant limitations, including longer process time, shortage of skilled manpower, laborious post-synthetic work-up, etc. To address the inherent limitations of batch processes, a novel approach was undertaken, resulting in the establishment and development of a visible light-assisted modular photo-flow reactor with a seamlessly integrated post-synthetic work-up procedure enabling the efficient synthesis of dihydropyranones from furfuryl alcohols. The reaction uses sun light as green energy source, and the novel photo-flow reactor platform developed with an integrated system enabling a downstream process in a time and labor-efficient manner which facilitates the Achmatowicz rearrangement, resulting in a fast (10 min) formation of the dihydropyranone products.

## Introduction

**Scheme 1:**
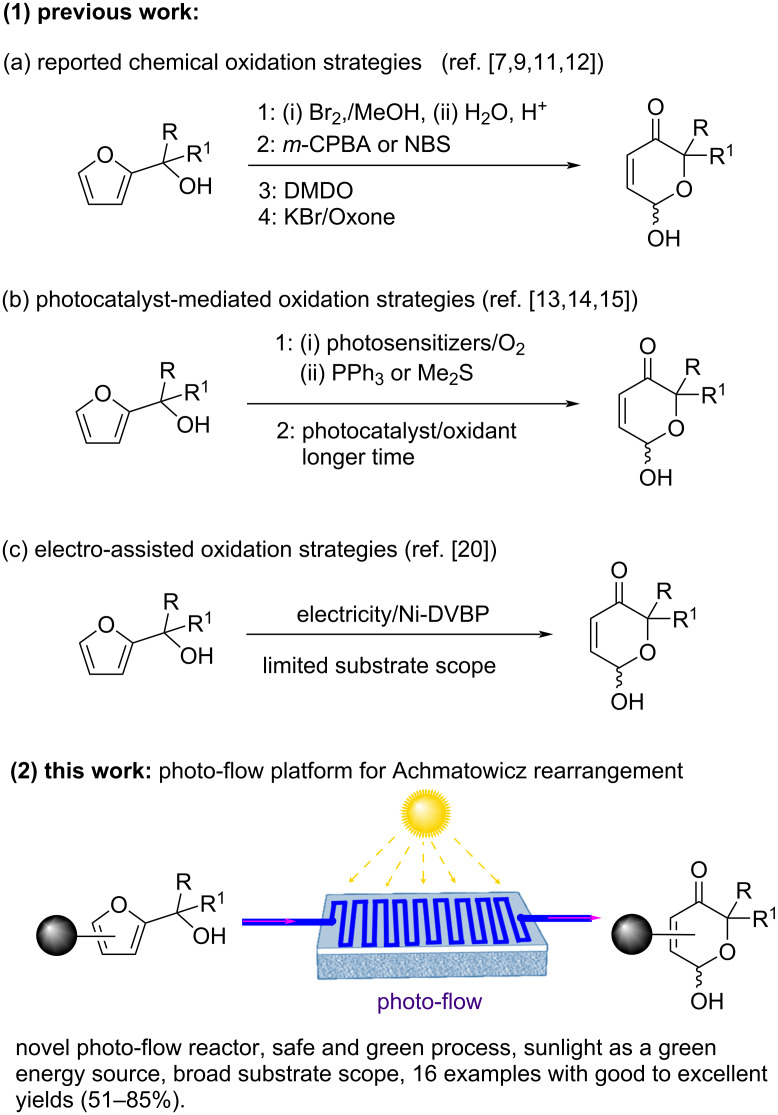
Strategies for Achmatowicz rearrangement.

The furan ring moiety is present in several natural products [1] and serves as a key precursor to 1,4-dicarbonyls [2], cyclopentanones [3], and carboxylic acids [4], in synthetic organic chemistry. Furfuryl alcohols, a family of 2-substituted furan molecules, are Achmatowicz rearrangement substrates for accessing highly decorated dihydropyranones [5]. In recent years, several groundbreaking approaches for the synthesis of dihydropyranones have been described by diverse groups of researchers [6]. These techniques do not require any pre-functionalization of non-prefunctionalized materials in order to proceed with the rearrangement. However, the Achmatowicz reaction or similar methodologies involve a catalytic to stoichiometric amount of oxidants such as *m*-CPBA [7], PCC [8], Br_2_ [9], NBS [10], DMDO [11], KBr/Oxone [12], Na_2_S_2_O_8_ [13], photosensitizers/O_2_^1^ [14], or Me_2_S [15], spirulina [16], Ti(OiPr)_4_/*t*-BuOOH [17], VO(acac)_2_)/*t*-BuOOH [18], and enzymatic oxidation [19] etc., which may compromise the environmental benefits (Scheme 1a,b) and take longer reaction times. Alternatively, a new green pathway is necessary for the Achmatowicz reaction to be performed in a faster and safer manner.

Towards this direction, few research groups have already reported the Achmatowicz reaction utilizing greener approaches, yet they suffered due to some limitations. Sun et al. [20] have successfully demonstrated an Achmatowicz rearrangement using electricity as a green oxidant (Scheme 1c) in a batch protocol. This electrochemical batch process utilizes electricity to enhance the vibrational energy of the substrate for the completion of the reaction and involves a noble metal, such as Ni-DVBP as an electrode. Also, the batch technique encounters a serious issue when considered for bulk production as a result of the expensive cost of Ni-DVBP and Ag/AgCl electrode, low surface-to-volume ratio, ineffective mixing, and sluggish heat exchange. The longer inter-electrode distances, may also lead to longer reaction times and poorer reaction selectivity and decreased yields [21]. Jeremy Robertson et al. [22] have demonstrated a combined use of flow and batch processes involving an electrochemical flow cell for the oxidation of furfuryl alcohols and subsequently utilizing the crude electrolysis mixture for hydrolysis in a traditional batch process to get the rearranged Achmatowicz product.

As a result, developing an alternative strategy would be an attractive solution to address the limitations described above. Flow chemistry was chosen as the most attractive alternative for the photo-induced (visible light) Achmatowicz rearrangement to convert furfuryl alcohol scaffolds into dihydropyranones due to its ability to better control of reaction conditions, including more uniform light exposure and improved mixing efficiency, which result in higher reaction rates and more consistent product quality. Additionally, the flow system enhances mass transfer and reduces reaction times, leading to more efficient processes and potentially higher yields compared to the batch processes [23]. The first photoredox-mediated Achmatowicz reaction was reported by Gilmore et al. [13] in batch mode utilizing furfuryl alcohols with Ru(bpy)_3_Cl_2_·6H_2_O as photocatalyst, Na_2_S_2_O_8_ as an oxidant and H_2_O/DMSO/MeCN as solvent system to get the product in 2 h excluding work-up procedure. However, safety concerns, long reaction time, downstream process in a time and labor-efficient manner and scalability of the product still arose and thus prompted us to initiate a flow process that could overcome the above challenges. To address these points, a novel continuous photo-flow platform for the Achmatowicz reaction including integrated post-synthetic work-up in a safe and faster manner with less intervention of human was developed (Scheme 1 (2)). This process involves a nature abundant energy source such as sun light and biomass-derived furfuryl alcohols as starting material making it more environmental benign.

## Results and Discussion

A batch process Achmatowicz rearrangement mediated by less expensive rose bengal as photocatalyst (PC) was first reported by Vassilikogiannakis et al. [24] and was further developed by Guan-Ben Du et al. [14]. Very often, the photolysis reactions performed in batch process encounter issues that include ineffective illumination, improper mixing, prolonged reaction durations, low quantum efficiency and mass transfer, a limited surface-to-volume ratio, along with an inadequate reaction selectivity [25]. Continuous flow technology has emerged as an attractive solution for many of these challenges, gaining popularity for its ability to efficiently address these issues. Additionally, this platform offers the advantage of integrating multiple steps, i.e., performing multistep transformations, then reaction extraction and separation, into a single process, which are typically performed individually/separately in batch methods. This enhances the overall efficiency for obtaining the desired product(s) from the reaction mixture. In continuation to our efforts on developing flow-based platforms, we herein present a photo-flow platform for Achmatowicz reactions. A novel photo-flow solar panel reactor was fabricated to test and validate the Achmatowicz rearrangement reaction (Figure S1, Supporting Information File 1), and the reaction conditions were optimized with a ruthenium catalyst. As illustrated in Figure S1, Supporting Information File 1, a flat photo-flow reactor was predesigned comprising a wide-surface polystyrene sheet (length 50 cm × width 50 cm × height 5 cm) bearing a reactor (PFA tubing) that was manually put and hooked on the polystyrene surface (Figure S1b, Supporting Information File 1). This reactor was then exposed to sunlight/handmade LED to carry out the Achmatowicz rearrangement reaction. To test the model reaction's feasibility, a stock solution containing ethyl 3-(furan-2-yl)-3-hydroxypropanoate (**2a**)/Ru(bpy)_3_Cl_2_·6H_2_O/K_2_S_2_O_8_/ACN (2 mL)/DMSO (2 mL)/H_2_O (4 mL) as a molar ratio of 1:1:0.005:70:54:408 was taken in one syringe and pumped to our fabricated photo-flow reactor (10 mL – PFR). The passing solution was then exposed to solar light (February to April sunlight in Hyderabad, India), to execute the Achmatowicz rearrangement, and a complete conversion was observed within 10 min (Table 1, entry 1). The Achmatowicz rearrangement product **3a** was obtained in 82% yield (Table 1, entry 1) using Ru(bpy)_3_Cl_2_·6H_2_O as a PC under solar light at 28–34 °C, light intensity 330000–322000 lux [26] with a flow rate of 1.0 mL min^−1^ in 10 mL reactor (10 min duration). A light-off experiment was performed to examine the Achmatowicz rearrangement's dependence on light, and it was observed that continuous light irradiation was required (Table 1, entry 2). Next, we considered running the process without utilizing the Ru(bpy)_3_Cl_2_·6H_2_O catalyst (Table 1, entry 3). There was no evidence of product formation, indicating that the Ru catalyst was required to pursue the photoinduced Achmatowicz rearrangement. Furthermore, it was observed that the product yield depended on resident time and it dropped over time as the residence time was reduced (see details in Supporting Information File 1, Table S1, entries 4–6) however, when the time was increased there was minimal enhancement in yield (see details in Supporting Information File 1, Table S1, entry 2). Following additional adjustment of the reaction conditions with various solvents, oxidants, lights, and photocatalysts, (see details in Supporting Information File 1, Table S1, entries 10–18), it was discovered that a combination of ACN/DMSO/H_2_O solvent, K_2_S_2_O_8_ oxidant, Ru(bpy)_3_Cl_2_·6H_2_O photocatalyst, and 28–34 °C temperature were the best-suited and optimized reaction conditions. After optimization, our efforts were put towards investigating for the selective elimination of the waste solvent using an integrated one-flow work-up procedure to generate **3a** with maximum practicality. Initially, the individual stages of the liquid–liquid extraction was achieved using the droplet micro fluidic method for extracting the compound (see details in Supporting Information File 1, Table S2). In this context, our previously in-house-developed liquid–liquid extractor was utilized (see details in Supporting Information File 1, Figure S4) [27]. After few trials towards optimization of extraction and separation, the best yield (82%) was obtained when EtOAc (flow rate set at 2.5 mL/min), water (flow rate set at 4.5 mL/min) were taken with the extraction time of 0.25 min and separation time of 0.11 min (see details in Supporting Information File 1, Table S2, entry 5). To verify the steadiness of the newly constructed integrated photo-flow system, we performed the experiment continuously for one hour under our optimized conditions, yielding 16.058 g day^−1^ production (see details in Supporting Information File 1, section 5.1). On the other hand, the traditional batch process requires longer durations to make an identical Achmatowicz rearrangement reaction [7–10,14–15].

**Table 1:** Optimization of continuous flow Achmatowicz reaction^a^.

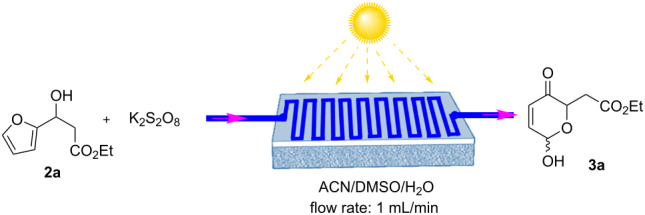		

Entry	Deviation from standard conditions	% Yield^b^

1	none	82
2	without light	NR
3	without Ru(bpy)_3_Cl_2_·6H_2_O	NR
4	without K_2_S_2_O_8_	NR
5	Na_2_S_2_O_8_ instead of K_2_S_2_O_8_	82
6	blue LED (30 W)	70
7	white LED (30 W)	80
8	RuCl_2_·6H_2_O instead of Ru(bpy)_3_Cl_2_·6H_2_O	32
9	methylene blue or rose bengal	trace

^a^Reaction conditions: **2a** (100 mg, 0.54 mmol, 1.0 equiv)/K_2_S_2_O_8_ (146 mg, 0.54 mmol, 1.0 equiv)/Ru(bpy)_3_Cl_2_·6H_2_O (2 mg, 2.6 µmol, 0.005 equiv)/ACN (2 mL)/DMSO (2 mL)/H_2_O (4 mL) in molar ratio of 1:1:0.005:70:54:408 and a reactor volume of 10 mL at room temperature; ^b^yields are based on isolated yields.

**Figure 1:**
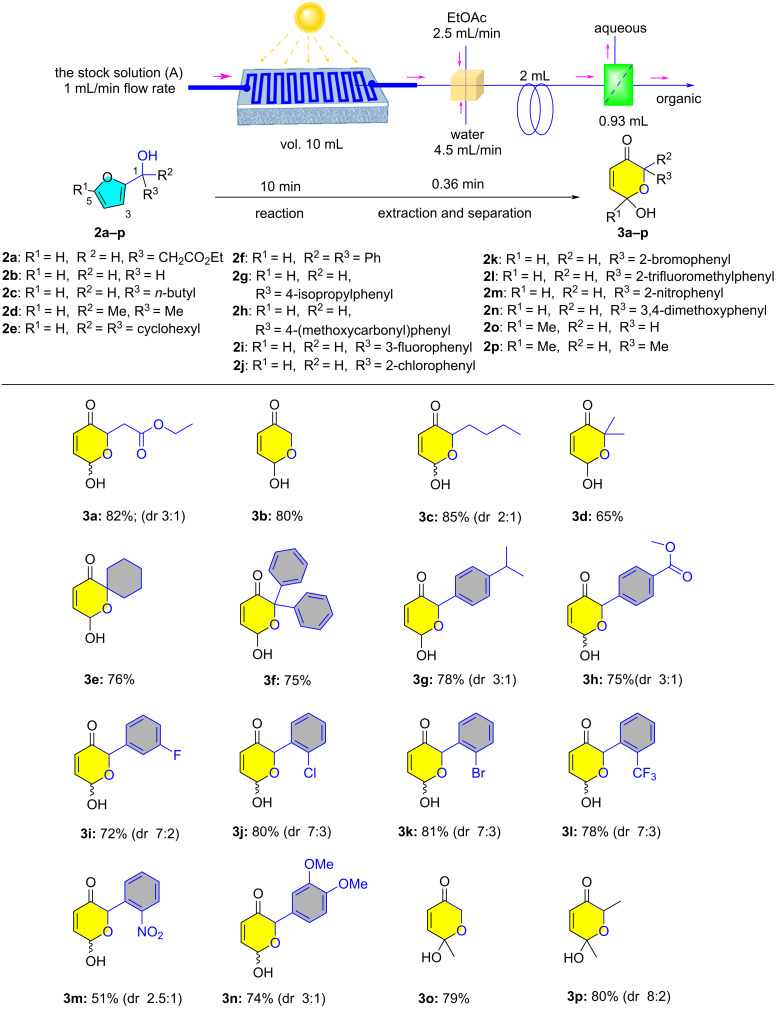
Scope of the integrated continuous photo-flow (visible light)-induced Achmatowicz rearrangement reaction. Reaction conditions: Stock solution (A) comprise of **2**/K_2_S_2_O_8_/Ru(bpy)_3_Cl·6H_2_O/ACN/DMSO/H_2_O in a molar ratio of 1:1:0.005:70:54:408; yields are based on isolated yields.

With the established photo-flow platform and optimized conditions, we were interested in investigating the scope of this methodology using several substituted furfuryl alcohol substrates (**2a–p**) which were subjected to the Achmatowicz reaction utilizing our photo-flow platform to provide the corresponding dihydropyranone products (**3a**–**p**) with moderate to good yields (51–85%), Figure 1.

We investigated several furanol derivatives by altering the substituents at two critical positions: the hydroxymethyl carbon and the 5-position of the furan ring, that were essential for the Achmatowicz reaction. Figure 1 demonstrates that even when there was no substituent at the hydroxymethyl position (in **2b**), the reaction proceeded effectively, leading to the formation of **3b** with an 80% yield inferring that the absence of substituents at the hydroxymethyl group does not prevent the formation of the desired product. At the same position, introducing more substituents such as *n*-butyl (**2c**), dimethyl (**2d**), cyclohexyl (**2e**) and sterically crowded diphenyl (**2f**) groups also led to the corresponding products (**3c**–**f**) with average to good yields that were comparable to that obtained from the unsubstituted furfuryl alcohol **2b**. These findings highlight the robustness of the Achmatowicz reaction in accommodating variations at these key sites. Next, the effect of the *p*-substituted phenyl group was also investigated by evaluating substrates **2g**, **2h** showing not much variations in the yields of the products **3g** and **3h**. The more electron-withdrawing *m*-fluorophenyl (**2i**), *o*-chlorophenyl (**2j**), *o*-bromophenyl (**2k**), *o*-trifluoromethylphenyl (**2l**), and *o*-nitrophenyl (**2m**) groups also resulted in comparable yields of **3g–m**, respectively, indicating no significant effect of substitutents on the aryl ring. A similar result was also obtained with dimethoxy-substituted phenyl moiety (**2n**) affording **3n** with a slightly lower yield of 74%. The substitution at C5-position on the furan ring in furfuryl alcohol derivatives were further investigated. A methyl group at the 5-position (**2o**, **2p**) resulted in the corresponding products (**3o**, **3p**) in good yields. All the products obtained were characterized by ^1^H NMR, ^13^C NMR and mass spectrometry techniques.

**Figure 2:**
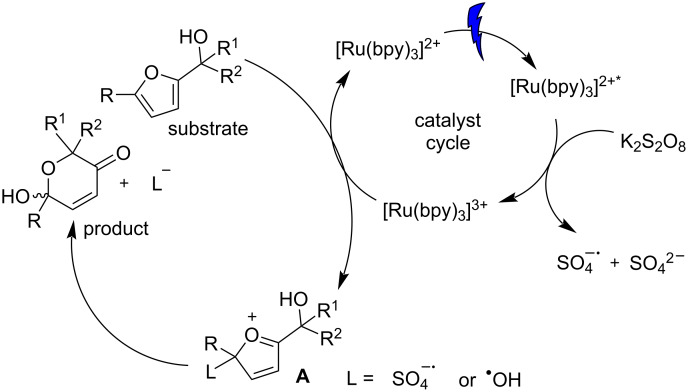
Proposed mechanism for the photochemically induced Achmatowicz rearrangement.

A plausible catalytic cycle has been postulated based on a literature study [13], and is shown in Figure 2. With the exposure of photocatalyst to sunlight/LED light, [Ru(bpy)_3_]^2+^ undergoes transition to [Ru(bpy)_3_]^2+*^ which is quenched by persulfate resulting in [Ru(bpy)_3_]^3+^ along with the simultaneous generation of sulfate and a sulfate radical. SET from furfuryl alcohol closes the catalytic cycle of the PC and an intermediate A is generated with L (L = SO_4_^−·^ or OH). The Achmatowicz product is formed by addition of water to oxocarbenium intermediate A followed by elimination of L.

## Conclusion

In conclusion, an integrated continuous PFR platform for photocatalytic functionalization of furfuryl alcohols to dihydropyranones through an Achmatowicz rearrangement is accomplished. The combined steps include fast reaction (10 min), and post-synthetic work-up (extraction time 0.25 min and separation time 0.11 min) in safe environment with minimal human intervention and avoiding hazardous chemical exposure. The process utilizes nature abundant energy sources such as sun light, as a greener approach and easily available biomass-derived furfuryl alcohols as starting materials. The flow platform with the developed protocol was utilized successfully to make several dihyropyranones that may find wide applications in medicinal and materials chemistry and natural product synthesis.

## Supporting Information

File 1 Experimental section and NMR spectra.

File 2 Video of the experimental set up.

## Data Availability

All data that supports the findings of this study is available in the published article and/or the supporting information of this article.
