# Supplementary material for: Visible-light-mediated flow protocol for Achmatowicz rearrangement
Source: Beilstein J Org Chem. 2024 Oct 8;20:2493–9. doi: 10.3762/bjoc.20.213 (PMC11472655; doi:10.3762/bjoc.20.213)
Supplement: File 1 — Experimental section and NMR spectra. [file Beilstein_J_Org_Chem-20-2493-s001.pdf]

BEILSTEIN JOURNAL OF ORGANIC CHEMISTRY

Supporting Information

for

Visible-light-mediated flow protocol for Achmatowicz rearrangement

Joachyutharayalu Oja, Sanjeev Kumar and Srihari Pabbaraja

Beilstein J. Org. Chem. **2024,** *20,* 2493–2499. doi:10.3762/bjoc.20.213

Experimental section and NMR spectra

License and Terms: This is a supporting information file under the terms of the Creative Commons Attribution License (https://creativecommons.org/licenses/by/4.0). Please note that the reuse, redistribution and reproduction in particular requires that the author(s) and source are credited and that individual graphics may be subject to special legal provisions.
The license is subject to the *Beilstein Journal of Organic Chemistry* terms and conditions: (https://www.beilstein-journals.org/bjoc/terms)

Table of Contents

Sl. No.	Description	Page NO.
**1.**	General information	S3
**2**	Procedure for the preparation of starting materials	S4–S15
**3.**	Fabrication of photo flow reactor and optimization conditions for the Achmatowicz reaction<br>3.1 Fabrication of photo flow reactor<br>3.2 optimization condition for Achmatowicz reaction	S16–S18
**4.**	Fabrication and design of micro separator and extraction, phase separation of **3a**<br>4.1 Fabrication and design of micro separator<br>4.2 In-line solvent exchange, extraction and separation of product **3a**<br>4.3 Quantum efficiency of photo reaction in flow	S19–S25
**5.**	General procedure for the integrated photo-flow Achmatowicz reaction, extraction and phase separation<br>5.1 Gram scale synthesis under optimized condition with 10 mL reactor.	S26–40
**6.**	Supporting references	S41
**7.**	NMR spectra	S42–S74

1. General information

1.1 Material and method used in experiments

Most of the reagents and chemicals were purchased from M/s. Sigma-Aldrich and M/s. AVRA chemicals, which were used without further purification, and demineralized water (18.2 mS conductivity) was used in the experiments. Purification procedures were carried out with reagent-grade solvents. Analytical thin-layer chromatography (TLC) was performed using analytical chromatography silica gel 60 F254 pre-coated plates (0.25 mm). The developed chromatogram was analyzed by UV lamp (254 nm). Polytetrafluoroethylene (PTFE) (id = 1000 μm) tubing, X-junction, syringe pump and high-purity Perfluoro alkoxy alkanes (PFA) (id =1000 μm) tubings were purchased from Upchurch IDEX HEALTH & SCIENCE. White LEDS, blue LEDS were all purchased from the M/s. Smart Chem. Synth. Pvt. Ltd. Hyderabad, India.

1.2 Measurement method

Nuclear magnetic resonance (NMR) spectra were recorded on Bruker, 500 and 400 MHz spectrometers in $CDCl_3$ solvent. Chemical shifts for ^1H NMR were expressed in parts per million (ppm) relative to tetramethylsilane (δ 0.00 ppm). Chemical shifts for ^{13}C NMR were expressed in ppm relative to $CDCl_3$ (δ 77.0 ppm) and data were reported as follows: chemical shift, multiplicity (brs = broad singlet, s = singlet, d = doublet, dd = doublet of doublets, t = triplet, q = quartet, m = multiplet), coupling constants (Hz), and integration. A Power-Sonic 405 sonication instrument was used for making homogeneous reaction mixture. IR analysis was recorded on ALPHA BRUKER spectrometer. The melting points were recorded on POLMAN MP-96 apparatus. A Waters-TOF spectrometer was used to record high-resolution mass spectra (HRMS).

2. Procedure for the preparation of starting materials

Compound **2b** was commercially available and other compounds **2a**, **2c**–**p** were synthesized following known protocols. Compounds **2h**,**i**,**l**,**m** were synthesized by new protocols.

Ethyl 3-(furan-2-yl)-3-hydroxypropanoate (2a)[1]:

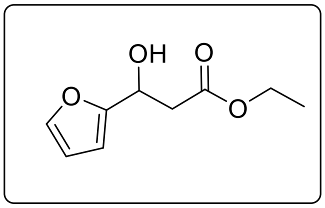

To a solution of diisopropylamine (8.02 mL, 57.29 mmol) in dry THF (40 mL) was added *n*-BuLi (1.60 M in hexane, 35.80 mL, 57.29 mmol) at –78 °C under a nitrogen atmosphere. Stirring was continued for 1 h, then anhydrous EtOAc (7.52 mL, 77.0 mmol) in THF (20 mL) was added at –78 °C. After 1 h, furfuraldehyde (4.31 mL, 52.08 mmol) in THF (20 mL) was added at –78 °C, and the mixture was stirred for 1 h before being quenched with aq. sat. NH_4Cl (20 mL). The reaction mixture was diluted with water (50 mL), extracted with EtOAc (3 × 30 mL) and the combined organic layer was washed with brine (40 mL), dried with anhydrous Na_2SO_4, filtered and concentrated under vacuum. The residue was purified by column chromatography (hexane/EtOAc 9:1) to give **2a** as a yellow oil, (9.0 g, 94%); R_f = 0.4 (hexane/EtOAc 4:1). IR (neat): υ_{max} 3453.51, 2985.77, 2931.08, 1727.80, 1160.19, 1020.73, 742.65 cm^{-1}. ^{1}H NMR (400 MHz, $CDCl_3$) δ: 7.38 (dd, *J* = 1.8, 0.8 Hz, 1H), 6.34 (dd, *J* = 3.3, 1.8 Hz, 1H), 6.29 (d, *J* = 3.3 Hz, 1H), 5.18 – 5.12 (m, 1H), 4.20 (q, *J* = 7.1 Hz, 2H), 3.25 (d, *J* = 5.1 Hz, 1H), 2.91 (dd, *J* = 16.5, 8.5 Hz, 1H), 2.84 (dd, *J* = 16.5, 4.2 Hz, 1H), 1.28 (t, *J* = 7.1 Hz, 3H). ^{13}C NMR (100 MHz, $CDCl_3$) δ: 172.09, 154.84, 142.38, 110.39, 106.44, 64.34, 61.11, 39.91, 14.28 ppm. HRMS (ESI) *m/z* $[M+Na]^+$ calcd for $C_9H_{12}NaO_4$: 207.0622 found: 207.0624

1-(Furan-2-yl)pentan-1-ol (2c)[2]:

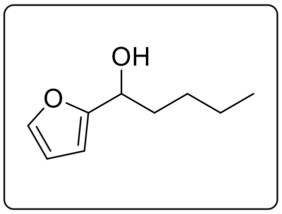

To a solution of furfural (960.8 mg, 10.0 mmol, 1.1 equiv) in dry THF (15 mL) at −78 °C under N_2 was added *n*-BuLi (3.6 mL, 9.1 mmol, 1.0 equiv, 2.5 M solution in hexanes). The reaction mixture was stirred further for 1.5 h at −78 °C. After 1.5 h, the reaction mixture was quenched with saturated aqueous NH_4Cl solution (50 mL) and extracted with EtOAc (3 × 25 mL). The combined organic layers were dried over with Na_2SO_4, filtered and concentrated in vacuum. The crude material was purified by silica gel column chromatography (EtOAc/hexane 1:4) to get **2c** as a yellowish oil (995.6 mg, 71% yield). R_f = 0.5 (10:1 hexane/ethyl acetate). IR (neat): υ_{max} 3368.31, 2939.77, 2864.06, 1147.44, 1004.37, 734.49 cm^{-1}. ^1H NMR (400 MHz, $CDCl_3$) δ: 7.37 (dd, *J* = 1.8, 0.8 Hz, 1H), 6.32 (dd, *J* = 3.2, 1.8 Hz, 1H), 6.22 (d, *J* = 3.2 Hz, 1H), 4.66 (t, *J* = 6.8 Hz, 1H), 1.95 (brs, 1H), 1.88 – 1.82 (m, 2H), 1.44 – 1.26 (m, 4H), 0.91 (t, *J* = 7.1 Hz, 3H). ^{13}C NMR (125 MHz, $CDCl_3$) δ: 157.05, 141.98, 110.22, 105.87, 67.96, 35.40, 27.83, 22.62, 14.13 ppm. HRMS (ESI) *m/z* $[M-OH]^+$ calcd for $C_9H_{13}O$: 137.0959 found: 137.0960

2-(Furan-2-yl)propan-2-ol (2d)[2]:

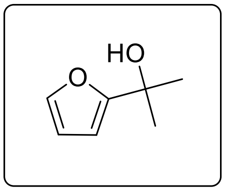

Under a nitrogen atmosphere, 2.2 mL of *n*-BuLi (2.5 M in hexane, 1.1 equiv) was added to the solution of furan (0.436 mL, 6.0 mmol, 1.2 equiv) in dry THF (15 mL) at 0 °C, and the

mixture was stirred at room temperature for 1 h. Then, a solution of acetone (0.382 mL, 5.0 mmol, 1.0 equiv) in THF (3 mL) was added into the reaction mixture dropwise at 0 °C. After stirring for 4 h, the reaction was quenched by water. The resulting solution was extracted three times with ethyl acetate (25 mL). The organic layer was dried over Na_2SO_4, filtered and concentrated under a vacuum. The residue was purified by silica gel column chromatography with hexane/ethyl acetate (9.5:0.5) as an eluent to give **2d** as clear oil (491.1 mg, 78% yield). R_f = 0.4 (9:1 hexane/ethyl acetate). IR (neat): υ_{max} 3387.80, 2973.17, 1368.40, 1155.80, 1012.52, 946.27, 731.88 cm^{-1}. ^1H NMR (400 MHz, $CDCl_3$) δ: 7.34 (dd, *J* = 1.7, 0.7 Hz, 1H), 6.29 (dd, *J* = 3.2, 1.8 Hz, 1H), 6.17 (d, *J* = 2.7 Hz, 1H), 2.26 (brs, 1H), 1.57 (s, 6H). ^{13}C NMR (125 MHz, $CDCl_3$) δ: 160.25, 141.51, 110.04, 103.59, 68.84, 28.70 ppm. HRMS (ESI) *m/z* $[M-OH]^+$ calcd for C_7H_9O : 109.0647 found : 109.0647

1-(Furan-2-yl)cyclohexan-1-ol (2e)[2]:

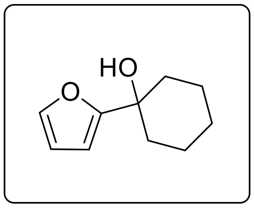

Under an nitrogen atmosphere, 2.2 mL of *n*-BuLi (2.5 M in hexane, 1.1 equiv) was added into the solution of furan (0.435 mL, 6.0 mmol, 1.2 equiv) in dry THF (15 mL) at 0 °C, and the mixture was stirred at room temperature for 1 h. Then, a solution of cyclohexanone (0.516 mL, 5.0 mmol, 1.0 equiv) in THF (3 mL) was added into the reaction mixture dropwise at 0 °C. After stirring for 4 h, the reaction mixture was quenched by water (20 mL). The resulting solution was extracted three times with ethyl acetate (25 mL). The organic layer was dried over Na_2SO_4, filtered and concentrated under vacuum. The residue was purified by silica gel column chromatography with hexane/ethyl acetate (9:1) as an eluent to give **2e** as yellowish oil, (851 mg, 81%), R_f = 0.37 (hexane/EtOAc, 10:1). IR (neat): υ_{max} 3423.72, 2933.87,

2860.50, 1715.16, 1449.30, 1214.40, 1016.20, 749.79 cm^{-1}. ^{1}H NMR (400 MHz, $CDCl_3$) δ: 7.34 (dd, *J* = 1.7, 0.6 Hz, 1H), 6.31 (dd, *J* = 3.2, 1.8 Hz, 1H), 6.20 (dd, *J* = 3.2, 0.6 Hz, 1H), 2.01 – 1.93 (m, 3H), 1.86 – 1.79 (m, 2H), 1.71 (tdd, *J* = 9.5, 5.9, 3.9 Hz, 2H), 1.54 – 1.43 (m, 3H), 1.39 – 1.32 (m, 1H). ^{13}C NMR (100 MHz, $CDCl_3$) δ: 159.98, 141.41, 110.05, 104.44, 70.13, 36.63, 25.52, 22.25 ppm. HRMS (ESI) *m/z* $[M-OH]^+$ calcd for $C_{10}H_{13}O$: 149.0966 found: 149.0973

Furan-2-yldiphenylmethanol (2f)[2]:

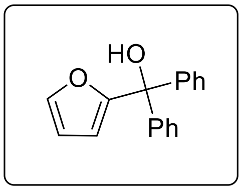

The furan (1.1 mL, 15.0 mmol) was dissolved in tetrahydrofuran (15 mL) and cooled to −78 °C. To this solution, *n*-butyllithium (6.6 mL, 16.5 mmol, 1.1 equiv, 2.5 M in hexanes) was added slowly and the cold solution was stirred for 1 h. The benzophenone (3.28 g, 16.5 mmol, 1.2 equiv) dissolved in tetrahydrofuran (10 mL) was added slowly over 30 min, the resulting mixture was stirred for 1 h at −78 °C and gradually warmed to room temperature overnight. The reaction mixture was quenched with saturated aqueous ammonium chloride solution (50 mL) and extracted with diethyl ether (3 × 20 mL). The combined organic extracts were washed with brine (40 mL), and dried over anhydrous sodium sulfate (Na_2SO_4), filtered and concentrated. The resulting crude alcohol was then isolated after column chromatography over silica gel (9:1, hexane/EtOAc as eluent) affording **2f** as a white solid (3.15 g, 84% yield), M.P = 94-96 ^{o}C, R_f = 0.4 (9:1 hexane/ethyl acetate). IR (neat): $υ_{max}$ 3417.68, 3061.41, 1490.72, 1445.13, 1335.03, 1147.14, 1009.99, 743.55, 694.15 cm^{-1}. ^{1}H NMR (400 MHz, $CDCl_3$) δ: 7.46 (dd, *J* = 1.8, 0.9 Hz, 1H), 7.35 – 7.30 (m, 10H), 6.34 (dd, *J* = 3.3, 1.8 Hz, 1H),

5.94 (dd, J = 3.3, 0.8 Hz, 1H), 3.11 (brs, 1H). ^{13}C NMR (125 MHz, $CDCl_3$) δ: 157.99, 144.74, 142.78, 128.12, 127.79, 127.34, 110.21, 109.77, 78.14 ppm. HRMS (ESI) *m/z* $[M-OH]^+$ calcd for $C_{17}H_{13}O$: 233.0966 found : 233.0957.

Furan-2-yl(4-isopropylphenyl)methanol (2g)[3]:

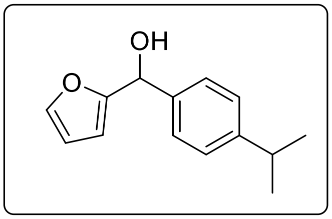

To a solution of furan (1.45 mL,20.0 mmol, 2 equiv) in THF (120 mL) was added *n*-BuLi (4.0 mL, 10 mmol, 2.5 M) at −20 0 °C under N_2, the mixture was warmed to 0 °C and stirred for 4 h. The above mixture was cooled to −78 °C, and 4-isopropyl benzaldehyde (1.51 mL, 10.0 mol, 1 equiv) in THF (10 mL) was added. The reaction mixture was warmed to rt and stirred for 1 h. The reaction was then quenched by addition of saturated aqueous NH_4Cl solution (50 mL), washed with brine (80 mL), dried over sodium sulfate. After filtration, the solvent was removed under reduced pressure and purified by silica gel column chromatography with hexane/ethyl acetate (9:1) as an eluent to give **2g** as yellowish oil (hexane/EtOAC, 9:1), (1.77 g, 82%), R_f = 0.45 (hexane/EtOAc, 10:1). IR (neat): $υ_{max}$ 3369.82, 2955.29, 2875.42, 1145.21, 1006.56, 829.12, 732.23 cm^{-1}. ^{1}H NMR (400 MHz, $CDCl_3$) δ: 7.40 – 7.39 (m, 1H), 7.36 (d, J = 8.2 Hz, 2H), 7.24 (d, J = 8.2 Hz, 2H), 6.32 (dd, J = 3.2, 1.8 Hz, 1H), 6.15 (d, J = 3.2 Hz, 1H), 5.79 (d, J = 4.3 Hz, 1H), 2.92 (dt, J = 13.8, 6.9 Hz, 1H), 2.46 (d, J = 4.5 Hz, 1H), 1.27 (s, 3H), 1.25 (s, 3H). ^{13}C NMR (100 MHz, $CDCl_3$) δ: 156.12, 148.84, 142.47, 138.34, 126.67, 126.60, 110.23, 107.31, 70.13, 33.90, 24.01 ppm. HRMS (ESI) *m/z* $[M-OH]^+$ calcd for $C_{14}H_{15}O$: 199.1115 found: 199.1117.

Methyl 4-(furan-2-yl(hydroxy)methyl)benzoate[4] (2h):

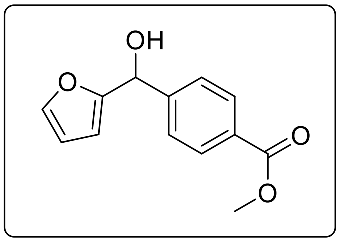

To a solution of furan (0.72 mL 10.0 mmol, 1 equiv.) in THF (100 mL) was added *n*-BuLi (4.0 mL, 10 mmol, 2.5 M) at −78 °C under N_2, the mixture was warmed to 0 °C and stirred for 1 h. The above mixture was cooled to −78 °C, and methyl 4-formylbenzoate (1.64 g, 10.0 mol, 1 equiv) in THF (10 mL) was added. The reaction mixture was warmed to rt and stirred for 1 h. The reaction was then quenched by addition of saturated aqueous NH_4Cl solution (50 mL), washed with brine (60 mL), dried over anhydrous sodium sulfate. Filtration followed by evaporation of the solvent under reduced pressure followed by purification over silica gel column chromatography with hexane/ethyl acetate as an eluent provided **2h** as solid (hexane/EtOAC 7:3), M.P = 80-83 ^{o}C. , (1.32 g 60%) R_f = 0.34 (hexane/EtOAc, 7:3). IR (neat): $υ_{max}$ 3297.35, 3137.97, 1716.57, 1409.04, 1287.22, 1257.46, 1110.02, 1007.59, 763.32 cm^{-1}. ^{1}H NMR (400 MHz, $CDCl_3$) δ: 8.02 (d, *J* = 8.5 Hz, 2H), 7.50 (d, *J* = 8.1 Hz, 2H), 7.38 (dd, *J* = 1.8, 0.8 Hz, 1H), 6.31 (dd, *J* = 3.2, 1.8 Hz, 1H), 6.11 (d, *J* = 3.3 Hz, 1H), 5.87 (d, *J* = 4.0 Hz, 1H), 3.90 (s, 3H), 2.75 (d, *J* = 4.4 Hz, 1H). ^{13}C NMR (100 MHz, $CDCl_3$) δ: 166.93, 155.26, 145.72, 142.83, 129.77, 126.51, 110.36, 107.80, 69.62, 52.19 ppm. HRMS (ESI) *m/z* $[M+H]^+$ calcd for $C_{13}H_{13}O_4$: 233.0814 found : 233.0845

3-Fluorophenyl)(furan-2-yl)methanol[12] (2i):

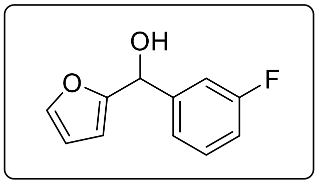

An oven-dried flask was evacuated and filled with nitrogen three times. To this flask was added 10 mL of THF, followed by furan (0.23 mL, 3.2 mmol, 1.6 equiv). The solution was

stirred and cooled to 0 °C, and then *n*-BuLi (1.5 mL, 1.6 M solution in hexanes) was added slowly. The reaction mixture was allowed to stir at 0 °C for 1 h at which point the reaction mixture was cooled to −78° C. The corresponding carbonyl compound 3-fluorobenzaldehyde (0.21 mL, 2 mmol, 1 equiv) was added slowly and the reaction mixture was allowed to stir and warm to room temperature over 16 h. The resulting reaction mixture was quenched under inert atmosphere via slow addition of 5 mL of saturated ammonium chloride solution followed by saturated aqueous sodium chloride solution (5 mL). The aqueous phase was extracted (3 × 10 mL) with ethyl acetate. The combined organic phase was dried over sodium sulfate, filtered and concentrated via rotary evaporator and purification over silica gel column chromatography with hexane/ethylacetate (9:1) as an eluent provided **2i** as oil (hexane/EtOAC 9:1), (80%), R_f = 0.45 (hexane/EtOAc, 9:1). IR (neat): υ_{max} 3352.06, 2881.03, 1594.74, 1444.03, 1239.61, 1136.69, 1010.91, 746.45 cm^{-1}. ^{1}H NMR (400 MHz, $CDCl_3$) δ 7.40 (dd, *J* = 1.8, 0.8 Hz, 1H), 7.33 (m, 1H), 7.20 – 7.16 (m, 2H), 7.04 – 6.98 (m, 1H), 6.33 (dd, *J* = 3.2, 1.8 Hz, 1H), 6.14 (d, *J* = 3.3 Hz, 1H), 5.81 (s, 1H), 2.56 (brs, 1H). ^{13}C NMR (101 MHz, $CDCl_3$) δ 163.01(d, J_{C-F} = 245.9 Hz), 155.42, 143.42 (d, J_{C-F} = 6.9 Hz), 142.89, 130.07 (d, J_{C-F} = 8.18 Hz), 122.28 (d, J_{C-F} = 2.4 Hz), 115.03 (d, J_{C-F} = 21.15 Hz), 113.73 (d, J_{C-F} = 22.45 Hz), 110.45, 107.80, 69.56 ppm. HRMS (ESI) *m/z* $[M-OH]^+$ calcd for $C_{11}H_8FO$: 175.0551 found : 175.0553.

(2-Chlorophenyl)(furan-2-yl)methanol[4] (2j):

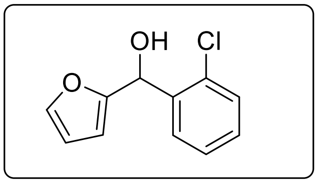

Under a nitrogen atmosphere, *n*-BuLi (1.7 mL, 4.14 mmol, 1.2 equiv) was added dropwise to a rapidly stirred solution of furan (0.4 mL, 5.50 mmol, 1.6 equiv) in THF (10 mL). The mixture

was stirred at room temperature for 2 h, and then cooled to −78 °C. The solution of *o*-chlorobenzaldehyde (0.4 mL, 3.45 mmo, l equiv) in THF (10 mL) was added dropwise, the mixture was stirred at −80 °C for 0.5 hour. After quenching by water, the product was extracted with ethyl acetate (2 × 15 mL) and the organic layer was washed with saturated aqueous NH_4Cl solution (10 mL) and brine (10 mL), dried over anhydrous Na_2SO_4, filtered and concentrated under vacuum. Purification by column chromatography (hexane/EtOAc, 9:1) provided **2j** as a yellow oil (609 mg, 82%). R*f* = 0.31 (hexane/ethyl acetate 4:1). IR (neat): υ_{max} 3344.42, 3065.05, 1443.41, 1189.24, 1139.53, 1011.23, 739.14 cm^{-1}. ^1H NMR (400 MHz, $CDCl_3$) δ: 7.68 (dd, *J* = 7.7, 1.5 Hz, 1H), 7.40 (m, 1H), 7.35 (m, 2H), 7.28 (dd, *J* = 7.6, 1.7 Hz, 1H), 6.32 (dd, *J* = 3.2, 1.9 Hz, 1H), 6.22 (d, *J* = 4.0 Hz, 1H), 6.08 (dd, *J* = 3.3, 0.6 Hz, 1H), 2.52 (d, *J* = 4.3 Hz, 1H). ^{13}C NMR (100 MHz, $CDCl_3$) δ: 154.59, 142.81, 138.36, 132.64, 129.61, 129.30, 128.21, 127.21, 110.47, 108.04, 66.95 ppm.. HRMS (ESI) *m/z* $[M\text{-}OH]^+$ calcd for $C_{11}H_8ClO$: 191.0256 found: 191.0258

(2-Bromophenyl)(furan-2-yl)methanol[5] (2k):

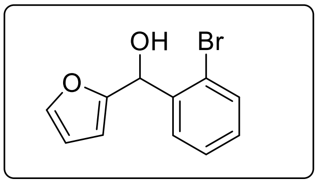

Under an nitrogen atmosphere, *n*-BuLi (1.7 mL, 4.14 mmol, 1.2 equiv) was added dropwise to a rapidly stirred solution of furan (0.4 mL, 5.50 mmol, 1.6 equiv) in THF (10 mL). The mixture was stirred at room temperature for 2 h, and then cooled to −78° C. The solution of *o*-bromobenzaldehyde (0.4 mL, 3.45 mmol, 1.6 equiv) in THF (10 mL) was added dropwise, the mixture was stirred at −78 °C for 0.5 hour. After quenching by water (10 mL), the product was extracted with ethyl acetate (2 × 15 mL) and the organic layer was washed with saturated

aq. NH_4Cl solution (10 mL) and brine (10 mL), dried over anhydrous Na_2SO_4, filtered and concentrated under vacuum. Purification by column chromatography (hexane/EtOAc 9:1) afforded **2k** as a yellow oil (813 mg, 93%); R_f = 0.6 (hexane/EtOAc 4:1). IR (neat): $υ_{max}$ 3346.28, 1440.67, 1192.12, 1142.27, 1009.26, 734.90 cm^{-1}. ^1H NMR (400 MHz, $CDCl_3$) δ: 7.68 (dd, *J* = 7.8, 1.2 Hz, 1H), 7.55 (d, *J* = 8.0 Hz, 1H), 7.41 – 7.35 (m, 2H), 7.19 (td, *J* = 7.9, 1.4 Hz, 1H), 6.31 (dd, *J* = 3.0, 1.8 Hz, 1H), 6.17 (s, 1H), 6.07 (d, *J* = 3.1 Hz, 1H), 2.90 (s, 1H). ^{13}C NMR (100 MHz, $CDCl_3$) δ: 154.58, 142.81, 139.95, 132.86, 129.59, 128.48, 127.82, 122.69, 110.48, 108.27, 69.16 ppm. HRMS (ESI) *m/z* $[M-OH]^+$ calcd for $C_{11}H_8BrO$: 234.9751 found : 234.9753

Furan-2-yl(2-(trifluoromethyl)phenyl)methanol (2l):

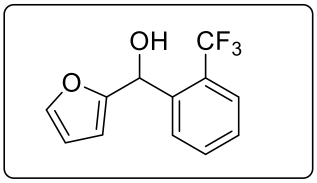

Under a nitrogen atmosphere, *n*-BuLi (1.7 mL, 3.64 mmol, 1.2 equiv) was added dropwise to a rapidly stirred solution of furan (0.4 mL, 4.85 mmol, 1.6 equiv) in THF (10 mL). The mixture was stirred at room temperature for 2 h, and then cooled to −78 °C. The solution of 2-(trifluoromethyl)benzaldehyde (0.4 mL, 3.0 mmol, 1 equiv) in THF (10 mL) was added dropwise, the mixture was stirred at −78 °C for 0.5 h. After quenching by water, the product was extracted with ethyl acetate and the organic layer was washed with saturated NH_4Cl solution (15 mL) and brine (15 mL), dried over anhydrous Na_2SO_4, filtered and concentrated under vacuum. Purification by column chromatography provided **2l** as colorless oil. (hexane/EtOAc, 9:1), (0.646 g, 88%), R_f = 0.28 (hexane/EtOAc, 1:1). IR (neat): $υ_{max}$ 3345.37, 1308.56, 1115.88, 1016.73, 740.65 cm^{-1}. ^1H NMR (400 MHz, $CDCl_3$) δ: 7.91 (d, *J* = 7.8 Hz, 1H), 7.66 (m, 2H), 7.44 (m, 1H), 7.39 (dd, *J* = 1.8, 0.8 Hz, 1H), 6.30 (dd, *J* = 3.3, 1.8 Hz, 1H),

6.25 (s, 1H), 6.05 (d, J = 3.3 Hz, 1H), 2.56 (s, 1H). ^{13}C NMR (126 MHz, $CDCl_3$) δ 155.1, 142.8, 139.5, 132.3, 129.1, 129.0, 128.3, 125.4 (q, J_{C-F} = 273 Hz), 125.7 (q, J_{C-F} = 5.5 Hz), 110.4, 108.0, 65.8 ppm. HRMS (ESI) *m/z* $[M-OH]^+$ calcd for $C_{12}H_8F_3O$: 225.0510 found: 225.0494

Furan-2-yl(2-nitrophenyl)methanol[13] (2m):

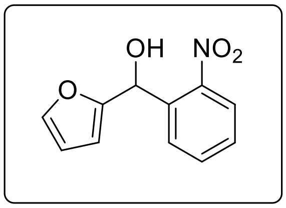

Under an argon atmosphere, *n*-BuLi (1.6 mL, 4.14 mmol, 1.2 equiv, 2.5 M in hexane) was added to a rapidly stirred solution of furan (0.4 mL, 5.50 mmol, 1.6 equiv) in THF (10 mL) dropwise. The mixture was stirred at room temperature for 2 h, and then cooled to −80 °C. The solution of 2-nitrobenzaldehyde (0.4 mL, 3.45 mmol, 1 equiv) in THF (10 mL) was added dropwise, the mixture was stirred at −80 °C for 0.5 h. After quenching by water, the product was extracted with ethyl acetate and the organic layer was washed with saturated aqueous NH_4Cl solution (20 mL) and brine (15 mL), dried over anhydrous Na_2SO_4, filtered and concentrated under vacuum. Purification by silica column chromatography (hexane/EtOAc, 8:2), provided **2m** as brown solid (0.657 g 87%), M.P = 50–53 ^oC, R_f = 0.21 (hexane/EtOAc, 8:2). IR (neat): $υ_{max}$ 3319.14, 3238.17, 2925.08, 2859.76, 1521.34, 1351.22, 1010.74, 722.65 cm^{-1}. ^{1}H NMR (400 MHz, $CDCl_3$) δ 7.99 (dd, J = 8.2, 1.2 Hz, 1H), 7.86 (dd, J = 7.9, 1.1 Hz, 1H), 7.68 (td, J = 7.7, 1.2 Hz, 1H), 7.52 – 7.46 (m, 1H), 7.37 (dd, J = 1.8, 0.8 Hz, 1H), 6.50 (s, 1H), 6.31 (dd, J = 3.2, 1.8 Hz, 1H), 6.14 (d, J = 3.3 Hz, 1H), 2.99 (brs, 1H). ^{13}C NMR (125 MHz, $CDCl_3$) δ 153.93, 147.95, 142.74, 136.02, 133.63, 129.10, 128.88, 124.83, 110.44, 107.90, 65.88 ppm. HRMS (ESI) *m/z* $[M-OH]^+$ calcd for $C_{11}H_8NO_3$: 202.0496 found : 202.0498

(3,4-Dimethoxyphenyl)(furan-2-yl)methanol[6] (2n):

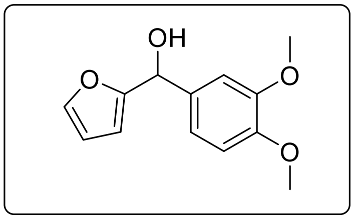

At –78 °C, to a solution of furan (1.36 g, 20 mmol, 2 equiv) in THF (60 mL) was added a solution of nBuLi (6.0 mL, 15 mmol, 1.5 equiv 2.5 M in hexanes). The reaction mixture was then warmed to 0 °C and stirred for 1 h. The reaction mixture was then cooled to –78 °C again, and a solution of 3,4-dimethoxybenzaldehyde (1.6 g, 10 mmol, 1 equiv) in THF (20 mL) was added dropwise. The mixture was allowed to warm to room temperature and stirred at the same temperature. The reaction progress was monitored by thin layer chromatography. Upon completion (2 h), the reaction mixture was cooled to 0 °C and quenched by dropwise addition of a saturated aqueous NH_4Cl solution (20 mL). The mixture was then extracted with EtOAc (50 mL × 3). The combined organic layers were dried over Na_2SO_4 and concentrated. The residue was purified by flash chromatography (hexanes/EtOAc = 8:2) to afford the pure alcohol product **2n** as dark brown oil (2.01 g, 86%). R_f = 0.4 (hexane/EtOAc, 8:2). IR (neat): υ_{max} 3494.29, 3001.18, 2938.38, 2837.06, 1723.65, 1594.22, 1510.35, 1456.41, 1255.92, 1233.16, 1135.93, 1075.17, 1015.97, 740.40 cm^{-1}. ^1H NMR (400 MHz, $CDCl_3$) δ: 7.39 (dd, *J* = 1.7, 0.7 Hz, 1H), 7.00 (d, *J* = 1.9 Hz, 1H), 6.94 (dd, *J* = 8.2, 1.9 Hz, 1H), 6.85 (d, *J* = 8.2 Hz, 1H), 6.32 (dd, *J* = 3.2, 1.8 Hz, 1H), 6.12 (d, *J* = 3.2 Hz, 1H), 5.77 (s, 1H), 3.87 (d, *J* = 2.3 Hz, 6H), 2.48 (s, 1H). ^{13}C NMR (100 MHz, $CDCl_3$) δ: 156.19, 149.13, 148.96, 142.62, 133.57, 119.12, 111.01, 110.36, 109.90, 107.40, 70.14, 56.05, 56.00 ppm. HRMS (ESI) *m/z* $[M-H]^+$ calcd for $C_{13}H_{13}O_4$: 233.0814 found : 233.0845

(5-Methylfuran-2-yl)methanol[7] (2o):

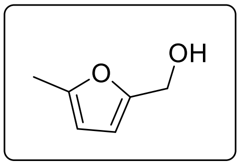

5-Methylfuran-2-carbaldehyde (1.0 g, 9.08 mmol, 1 equiv) was dissolved in anhydrous CH_3OH (10.0 mL) in a 50 mL round-bottom flask under nitrogen. $NaBH_4$ (0.678 g, 18.2 mmol, 2 equiv) was added, and the mixture was stirred for 16 h at room temperature after evaporation of MeOH under reduced pressure. Then H_2O (25 mL) was added, the aqueous phase was extracted with dichloromethane (15 mL). The organic layer was dried with Na_2SO_4 and filtered. Evaporation of the solvent afforded (5-methylfuran-2-yl)methanol (**2o**) as an orange liquid (1.01 g, 98%), R_f = 0.21 (hexane/EtOAc, 8:2). IR (neat): $υ_{max}$ 3418.28, 2927.19, 1721.28, 1375.21, 1216.85, 1011.91, 932.18, 782.72 cm^{-1}. ^1H NMR (400 MHz, $CDCl_3$) δ 6.16 (d, *J* = 3.0 Hz, 1H), 5.91 (d, *J* = 2.0 Hz, 1H), 4.53 (d, *J* = 3.9 Hz, 2H), 2.28 (s, 3H), 2.02 (s, 1H). ^{13}C NMR (125 MHz, $CDCl_3$) δ 152.55, 152.37, 108.90, 106.36, 57.66, 13.68 ppm. HRMS (ESI) *m/z* $[M-OH]^+$ calcd for C_6H_7O: 95.0497 found: 95.0522

1-(5-Methylfuran-2-yl)ethan-1-ol[11] (p):

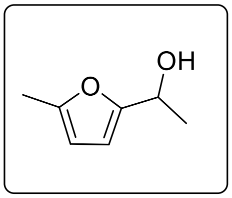

To the solution of 5-methylfuran-2-carbaldehyde (1.0 g, 9.08 mmol, 1 equiv) in THF (10.0 mL) was added a MeLi solution (2.9 mL, 9.08 mmol, 1.0 equiv 3.1 M in dimethoxymethane) under nitrogen atmosphere at −78 °C. The reaction mixture was stirred for 1 h, after completion of reaction indicated by TLC the reaction mixture was quenched with saturated ammonium chloride solution. The organic layer was extracted with ethyl acetate dried over Na_2SO_4 and concentrated via a rotary evaporator to afford 1-(5-methylfuran-2-yl)ethan-1-ol as yellow oil (1.13 g, 99%). IR (neat): $υ_{max}$ 3349.19, 2980.47, 2926.55, 2881.39, 1219.04, 1076.73, 1017.81, 950.10, 880.39, 783.00 cm^{-1}. ^1H NMR (400 MHz, $CDCl_3$) δ 6.09 (d, *J* = 3.1 Hz, 1H), 5.90 – 5.88 (m, 1H), 4.82 (q, *J* = 6.5 Hz, 1H), 2.28 (d, *J* = 0.7 Hz, 3H), 1.98 (s,

1H), 1.52 (d, J = 6.6 Hz, 3H). ^{13}C NMR (101 MHz, $CDCl_3$) δ 155.91, 151.78, 106.07, 63.74, 21.29, 13.65. ppm. HRMS (ESI) m/z $[M-OH]^+$ calcd for C_7H_9O: 109.0647 found: 109.0647

3. Fabrication of photo flow reactor and optimization conditions for the Achmatowicz reaction

3.1 Fabrication of flow reactor.

For the Achmatowicz photo-flow reaction, we used a homemade photo-flow reactor. To fabricate the solar panel reactor, we used a polystyrene foam sheet, aluminum foil, PFA tubing, and cello-tape. The polystyrene foam (length 50 cm × width 50 cm × height 5 cm) was covered by aluminum foil (0.016 mm) for reflecting sunlight. Then manually, PFA tubing (OD 1/16", ID 1.0 mm, Length = 12.8 m, volume = 10 mL) was mounted over the aluminum-covered surface (Figure S1 (b)).

(a)

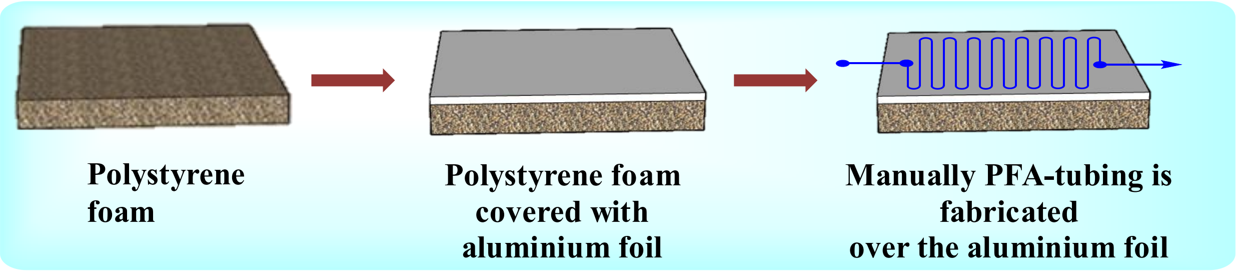

(b)

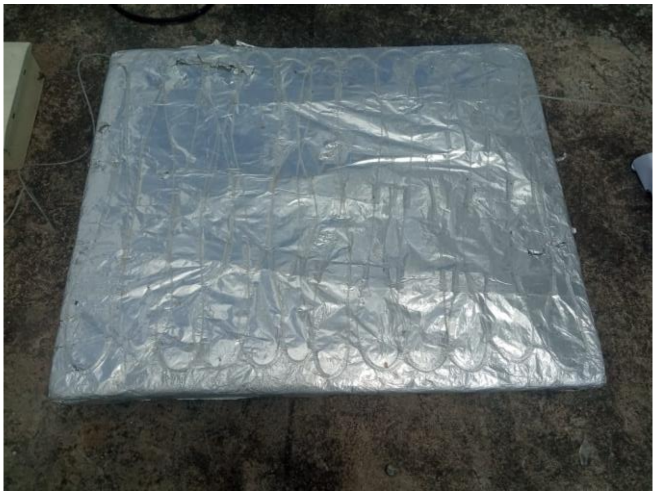

Figure S1. (a) homemade fabrication step for a solar panel reactor (polystyrene foam size length 50 cm × width 50 cm × height 5 cm); (b) real image of homemade fabricated solar panel reactor.

3.2 Optimization conditions for Achmatowiz reaction

In a systematic one-flow synthesis protocol for the Achmatowicz reaction, a test tube was charged with potassium persulfate (146 mg, 0.54 mmol, 1.0 equiv) and $Ru(bpy)_3Cl_2$•$6H_2O$ (2 mg, 2.6 µmol, 0.005 equiv). Subsequently, water (4 mL) was introduced, followed by the addition of the furfuryl alcohol substrate (100 mg, 0.54 mmol, 1.0 equiv) in a 1:1 acetonitrile/DMSO mixture (4 mL). The resulting reaction mixture was then sonicated by using a Power-Sonic 405 sonication instrument for making a homogeneous reaction mixture. Then, the mixture was transferred into a 10 mL BD syringe and delivered into the reactor under sunlight with varied flow rate, light, oxidant, solvents screening and photo-catalysts (see optimization Table S1). After screening all these conditions, we found that a combination of furfuryl alcohol, with $K_2S_2O_8$ oxidant, $Ru(bpy)_3Cl_2\cdot 6H_2O$ photocatalyst in ACN/DMSO/H_2O solvent in molar ratio of (1:1:0.005:70:54:408), in a 10 mL photo-flow reactor were the best-suited and optimized reaction conditions for an optimal yield of the product.

Table S1: Optimization of the continuous flow Achmatowicz reaction.

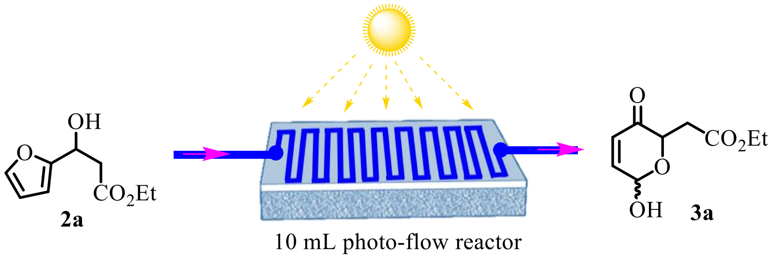

Entry	Deviation from standard conditions	% Yield[a]
1	None	82
2	0.5 mL/min. instead of 1 mL/min	84
3	0.75 mL/min. instead of 1 mL/min	82
4	1.5 mL/min. instead of 1 mL/min	71
5	2.0 mL/min. instead of 1 mL/min	45
6	2.5 mL/min. instead of 1 mL/min	30
7	without light	NR
8	without $Ru(bpy)_3Cl_2{\cdot}6H_2O$	NR
9	without $K_2S_2O_8$	NR
10	acetone:H_2O instead of ACN:DMSO:H_2O	55
11	IPA:H_2O instead of ACN:DMSO:H_2O	20
12	MeOH:H_2O instead of ACN:DMSO:H_2O	42
13	ACN:H_2O instead of ACN:DMSO:H_2O	45
14	$Na_2S_2O_8$ instead of $K_2S_2O_8$	82
15	blue LED (30 W)	70
16	white LED (30 W)	80
17	$RuCl_2.6H_2O$ instead of $Ru(bpy)_3Cl_2.6H_2O$	32
18	methylene blue, rose bengal	trace

Reaction conditions: 2a (100 mg, 0.54 mmol, 1.0 equiv):$K_2S_2O_8$ (146 mg, 0.54 mmol, 1.0 equiv):$Ru(bpy)_3Cl_2{\cdot}6H_2O$ (2 mg, 2.6 µmol, 0.5 mol %):ACN (2 mL):DMSO (2 mL):H_2O (4 mL) in molar ratio (1:1:0.005:70:54:408), reactor volume (10 mL), room temperature; [a]yields are based on isolated yield.

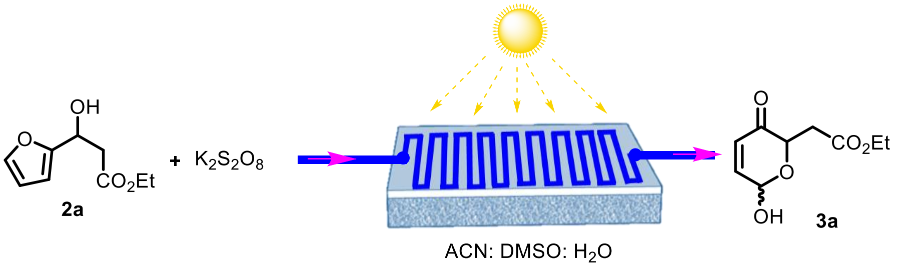

Figure S2. Schematic graphic presentation of photo-flow setup for Achmatowicz reaction.

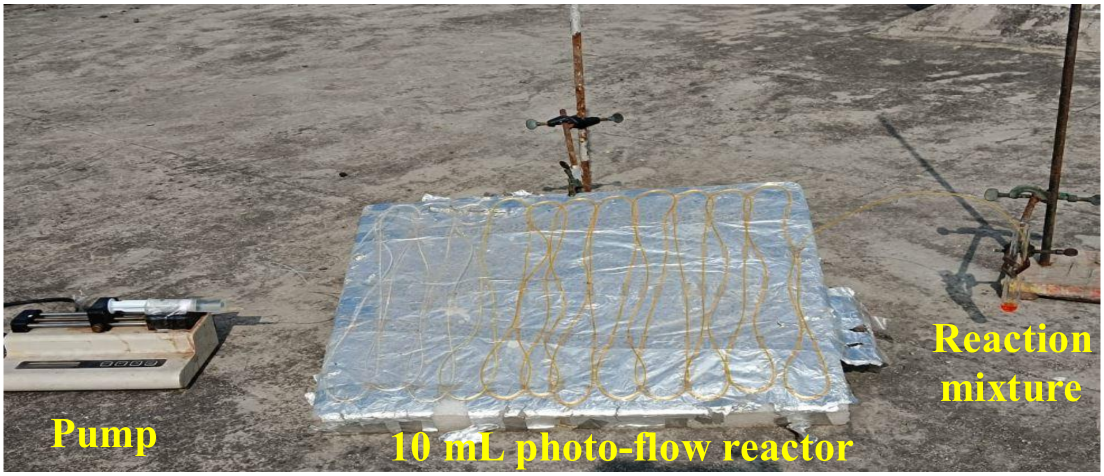

Figure S3. Snapshot of real-experimental set-up for the Achmatowicz reaction.

4. Fabrication and design of micro separator and extraction, phase separation of 3a.

4.1 Fabrication and design of micro separator. Further to minimize the post-downstream work-up process, we fabricated a micro separator with a stainless steel body (Figure S4, a) (60 mm length × 60 mm width × 10 mm thickness). The first Teflon (60 mm length × 60 mm width × 1 mm thickness) (Figure S4, b) layer was made with a laser cutter to protect the stainless steel. The second layer consists of a laser-grooved Teflon film (length = 3 mm × width = 1 mm × 2 mm height) (Figure S4, c) zig-zag groove with cuboids shape and the third layer consisting of cylindrical holes (r = 1.5 mm,1 mm thickness) (Figure S4, d). To align the film patterns, the four corners of each Teflon film were drilled to make a hole (1 mm diameter). Thereafter, a polypropylene coated polytetrafluoroethylene (PTFE) membrane (Whatmann, 0.45 μm pore, 47 mm diameter.) was sandwiched between two Teflon sheets with identical dimensions to fit the groove channels and coupled to each other by inserting metal pins through the holes at the film corners. Finally, the metal holder was tightly screwed to pack all the layers of the device, to ensure no leaks. Then we appended the continuous droplet based extraction, phase separation.

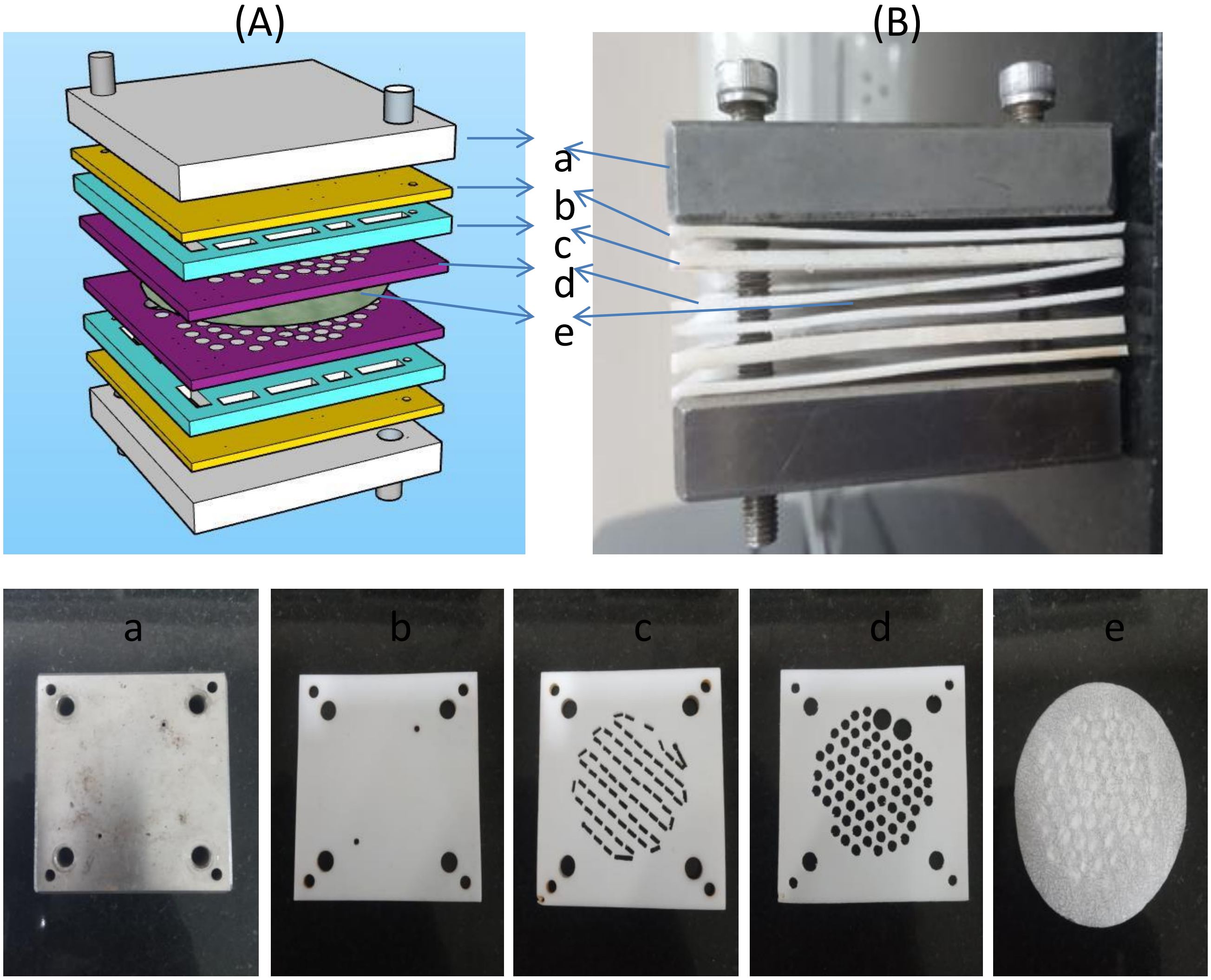

Figure S4. Illustration of a fluoropolymer PTFE membrane-based micro separator; **(A)** 3D model; **(B)** original photograph; **a**. SS-metal body; **b**. metal protecting PTFE layer; **c** laser grooved PTFE zig-zag channel; **d**. laser grooved PTFE cylindrical channel **e**. propylene coated PTFE membrane.

4.2 In-line solvent exchange, extraction and separation of product 3a.

To exchange the solvent by extracting the product from DMSO/ACN the additional PTFE membrane embedded phase separator was connected with the crude reaction mixture of **3a** from the outlet of the photo-flow reactor under standard Achmatowicz reaction condition. A sequential process of droplet formation, extraction and separation for purification of the

product **3a** was obtained with the PTFE membrane micro separator, as explained in a step-wise manner below. At first, the flow rate (1 mL/min) of **3a** the crude reaction mixture solution was passed then alternating organic-aqueous droplets were formed by introducing varied flow rates of water and ethyl acetate into the product mixture in DMSO/ACN through X-junction. Then, aqueous-organic droplet was smoothly passed through perfluoro alkoxy (PFA) tubing (id = 1000 µm, l = 2.5 m, vol. = 2 mL) for the extraction to occur. Finally, the mixture of organic phase containing **3a** was selectively separated by wetting and penetrating through the PTFE membrane of micro separator (vol. = 0.93mL) into the bottom channel of the separator, whereas the aq DMSO containing aqueous phase did not wet the membrane to stay at the upper channel, and gone to the waste (Table 2).

Table S2. Solvent exchange through the micro-separator for **3a** separation.

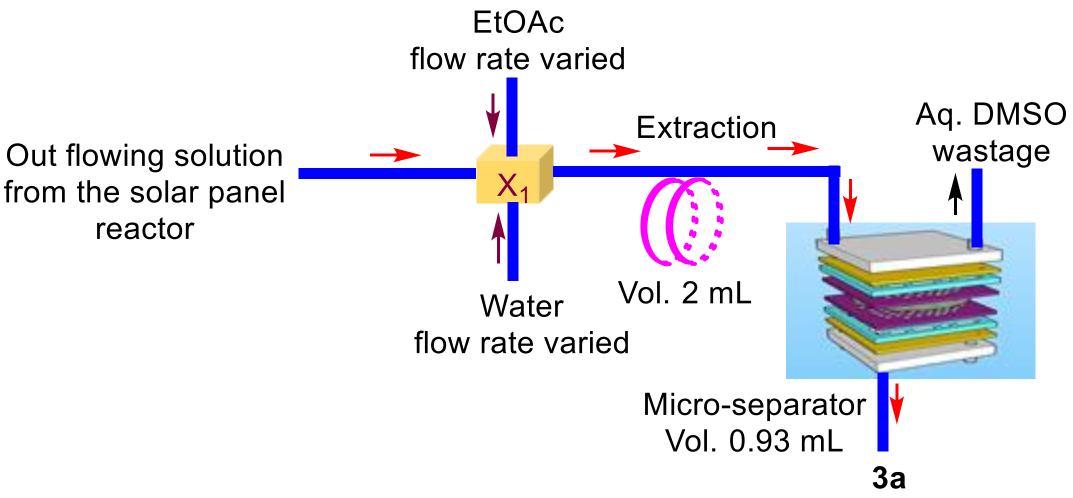

Entry	Flow rate (mL/min)		Extraction time (min.)	Separation time (min.)	% Yield[a] 3a
	EtoAc	Water			
1	1	3	0.4	0.18	66
2	1.5	4	0.3	0.14	71
3	2	4.5	0.2	0.12	79

4	2.5	5	0.23	0.10	80
**5**	**2.5**	**4.5**	**0.25**	**0.11**	**82**
6	3	4.5	0.23	0.10	74
[a]Isolated yields based on average of two experiments.					

5. General procedure for an integrated photo-flow Achmatowicz reaction, extraction, phase separation.

After the successful completion of the individual Achmatowicz reaction optimization, extraction and separation of the product, we have interconnected all the pre-optimized reactor sets in a continuous flow manner (Figure S5. (a)). The homogeneous stock solution (**A**) containing **2a**: (100 mg, 0.54 mmol, 1.0 equiv):$K_2S_2O_8$ (146 mg, 0.54 mmol, 1.0 equiv):$Ru(bpy)_3Cl_2{\cdot}6H_2O$ (2 mg, 2.6 µmol, 0.005 equiv):ACN (2 mL):DMSO (2 mL):H_2O (4 mL) in molar ratio (1:1:0.005:70:54:408) was taken in a syringe (10 mL BD syringe) and connect with the pump. The solution was passed through a photo-flow reactor for the synthesis of the Achmatowicz rearrangement product under optimal conditions (flow rate = 1 mL/min) during 10.0 min. of residence time under sunlight. The out flowed product mixture **3a** was directly connected with an additional X-junction, then alternating organic-aqueous droplets were formed by introducing cool water (flow = 4.5 mL/min) and ethyl acetate (flow = 2.5 mL/min) into the product mixture in DMSO/ACN through the X-junction. Then the aqueous-organic droplets were smoothly passed through perfluoro alkoxy (PFA) tubing (*id* = 1000 µm, *l* = 2.56 m, vol. = 2 mL) for the extraction of product **3a** during 0.25 min. Finally, the mixture of organic phase containing **3a** was selectively separated during 0.11 min by wetting and penetrating through the PTFE membrane of micro separator (vol = 0.93 µL) into the bottom channel of the separator, whereas the aq. DMSO containing aqueous phase did

not wet the membrane to stay at the upper channel, and gone to the waste (Figure S5. (b)). The extracted waste aqueous layer was further extracted with ethyl acetate and analyzed by NMR and confirmed by the absence of the corresponding peaks in the crude NMR analysis (^{1}H and ^{13}C NMR spectra) which showed no product. The ethyl acetate layer was concentrated under a vacuum to give the product and subsequent purification by column chromatography.

(a)

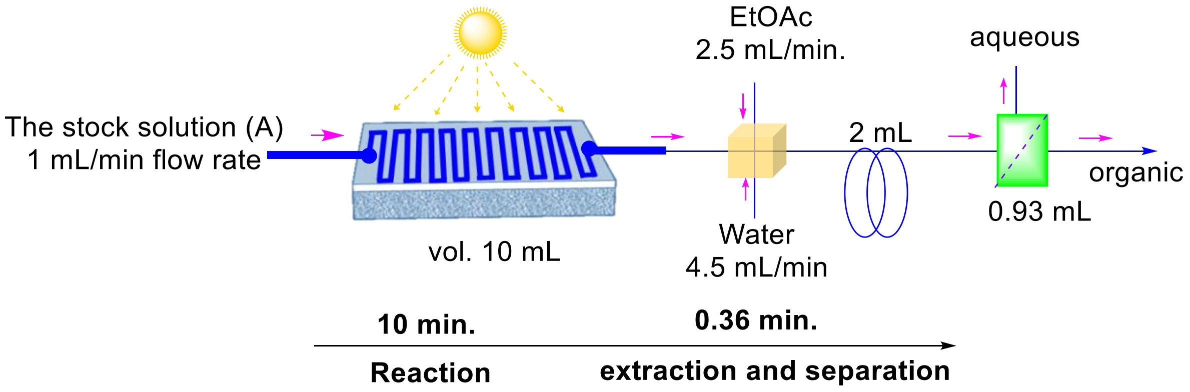

(b)

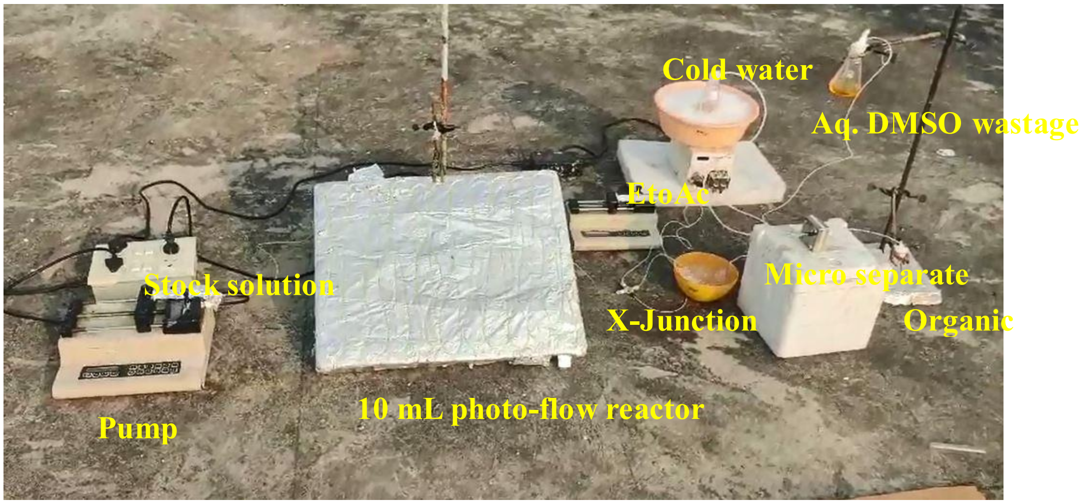

Figure S5. (a) Schematic graphic presentation of an integrated photo-flow Achmatowicz reaction, extraction, phase separation in continues flow manner. (b) Real image of an integrated photo-flow Achmatowicz reaction, extraction, phase separation set up.

Quantum efficiency of flow photo reaction (ϕ):

Starting compound molecular weight (MW start): 184 g/mol

Product compound molecular weight (MW product): 200 g/mol

Reaction time: 10 minutes

Product yield: 82%

Sun light wave length = 460 nm = 460 $\times 10^{-9}$ m

power of sun light in Hyderabad (for 8 h) = 5.44 kWh/m^2/day

power of sun light in Hyderabad (for 1 h) = $\frac{5.44}{8} = 0.68$ kWh/m^2

= 680 W/m^2

PFA tubing: Outer diameter (OD): 1/16-inch, Inner diameter= 0.79 $\times 10^{-3}$ m

(ID): 1.0 mm, length: 12.8 m, volume: 10 mL

Calculate the outer surface area of the PFA tubing = $2\pi rL$

$=2 \times 3.14 \times 0.79 \times 10^{-3} \times 12.8 = 63.50336 \times 10^{-3} = 0.0635$ m^2

Power of sunlight on PFA tubing = 0.0635 $m^2 \times$ 680 W/m^2

=43.18 W/m^2

Calculation for the number of photons incidence in 10 minutes:

$N_{photons} = \frac{P\lambda t}{hc}$

P = power of light = 43.18W = 43.18 Js^{-1}

λ= Wave length of light = 460 nm = 460 $\times 10^{-9}$

t = Duration of irradiation time,10 minutes = 600 s

h= Plank's constant = 6.626×10^{-34} Js^{-1}

c = Velocity of light = 3×10^8 m s^{-1}

$$= \frac{43.18 \times 460 \times 10^{-9} \times 600}{6.626 \times 10^{-34}\ 3 \times 10^8}$$

$$= \frac{11917680}{19.878} \times 10^{17}$$

$= 599541.201 \times 10^{17} = 5.99 \times 10^{22}$

Calculation for the Moles of photons incidence in 10 minutes = $\frac{\text{N photons}}{\text{N}}$

[N=Avogadro's number = 6.023×10^{23}

$= \frac{5.99 \times 10^{22}}{6.023 \times 10^{23}}$ = 0.0995 moles of photon incidence in 10 minutes

Calculation for the Moles of product formed in 10 minutes = $\frac{\textbf{Productivity in 10 minutes}}{\textbf{Molecular weight of product}}$

The product formed for 10 minutes:

$$\frac{\text{Concentration of reaction mixture } \times \text{flow rate} \times \text{ time of reaction} \times \text{ yied of product} \times \text{product M.wt}}{1000}$$

$$= \frac{0.068 \times 1 \times 10 \times 0.82 \times 200}{1000}$$

=0.1152 g/10 minutes

Calculation for the Moles of product formed in 10 minutes =$\frac{\textbf{Productivity in 10 minutes}}{\textbf{Molecular weight of product}}$

$= \frac{0.115268}{200} = 0.00057634$

$= 5.576 \times 10^{-4}$

Quantum efficiency (ϕ) in flow =$\frac{\textbf{Moles of product formed in 10 minutes}}{\textbf{Moles of photons incidence}} \times 100$

$= \frac{5.576 \times 10^{-4}}{0.0995} \times 100 = 56.0402 \times 10^{-2}$ = **0.56%**

5.1 Gram scale synthesis under optimized conditions with 10 mL reactor.

To validate the long time steadiness of the newly developed photo-flow protocol with continuous system, we have performed the gram scale synthesis under optimized conditions with 10 ml reactor. The homogeneous stock solution (**A**) containing **2a**: (1 g, 5.4 mmol, 1.0 equiv): $K_2S_2O_8$ (1.46 g, 5.4 mmol, 1.0 equiv):$Ru(bpy)_3Cl_2·6H_2O$ (20 mg, 26 µmol, 0.005 equiv):ACN (20 mL):DMSO (20 mL):H_2O (40 mL) in molar ratio (1:1:0.005:70:54:408) was taken in a siring (100 mL BD siring) and connect with the pump. The solution was passed through a photo-flow reactor for the synthesis of the Achmatowicz Rearrangement product under optimal conditions (flow rate = 1 mL/min) during 10.0 min of residence time under sunlight. The out flowing product mixture **3a** was directly connected with additional X-junction, then alternating organic-aqueous droplets were formed by introducing cool water (flow = 4.5 mL/min) and ethyl acetate (flow = 2.5 mL/min) into the product mixture in DMSO/ACN through X-junction. Then aqueous-organic droplets were smoothly passed through perfluoro alkoxy (PFA) tubing (*id* = 1000 µm, *l* = 2.56 m, *vol* = 2 mL) for the extraction of product **3a** during 0.25 min. Finally, the mixture of organic phase containing **3a** was selectively separated during 0.11 min by wetting and penetrating through the PTFE membrane of micro separator (*vol* = 0.93 µL) into the bottom channel of the separator, whereas the aq. DMSO containing aqueous phase did not wet the membrane to stay at the upper channel, and gone to the waste (Figure S4. (b)). The extracted waste aqueous layer was further extracted with ethyl acetate and analyzed by NMR and confirmed by the absence of the corresponding peaks in the crude NMR analysis (^{1}H and ^{13}C NMR spectra) which showed no product. The ethyl acetate layer was concentrated under a vacuum to give the product and subsequent purification by column chromatography using hexane and ethyl

acetate as mobile phase (hexane/EtOAC, 7:3), to afford **3a** as yellowish oil (891 mg, 82%; d.r. 3:1) as a diastereomeric mixture that was not separated. R_f = 0.5 (hexane/EtOAc, 7:3). IR (neat): υ_{max} 3435.91, 2983.25, 2937.84, 1723.15, 1697.74, 1374.75 1353.86, 1288.14, 1179.65, 1089.67, 1019.80 cm^{-1}. Major diastereomer: ^{1}H NMR (400 MHz, $CDCl_3$) δ: 6.92 (dd, *J* = 10.3, 3.5 Hz, 1H), 6.15 (d, *J* = 10.3 Hz, 1H), 5.65 (t, *J* = 3.8 Hz, 1H), 5.03 (dd, *J* = 7.6, 3.9 Hz, 1H), 4.17 (qd, *J* = 7.1, 4.6 Hz, 2H), 3.66 (d, *J* = 4.7 Hz, 1H), 3.00 (dd, *J* = 16.8, 3.9 Hz, 1H), 2.75 (dd, *J* = 16.7, 7.6 Hz, 1H), 1.27 (t, *J* = 7.1 Hz, 3H). ^{13}C NMR (101 MHz, $CDCl_3$) δ: 194.95, 170.99, 144.49, 127.35, 87.89, 70.95, 61.15, 35.43, 14.27.ppm. Minor diastereomer: ^{1}H NMR (400 MHz, $CDCl_3$) δ: 6.96 (dd, *J* = 10.3, 1.6 Hz, 1H), 6.19 (dd, *J* = 10.3, 1.5 Hz, 1H), 5.71 (dd, *J* = 7.4, 1.3 Hz, 1H), 4.59 (ddd, *J* = 7.9, 3.9, 1.2 Hz, 1H), 4.17 (qd, *J* = 7.1, 4.6 Hz, 2H), 3.91 (d, *J* = 7.4 Hz, 1H), 3.01 (dd, *J* = 16.7, 4.0 Hz, 1H), 2.84 – 2.71 (m, 1H), 1.27 (t, *J* = 7.2 Hz, 3H). ^{13}C NMR (100 MHz, $CDCl_3$) δ: 194.48, 170.99, 148.20, 128.49, 90.96, 75.50, 61.28, 36.29, 14.27 ppm. HRMS (ESI) *m/z* $[M+Na]^+$ calcd for $C_9H_{12}NaO_5$: 223.0582 found: 223.0565

Productivity under optimized condition

Molar solution = 0.068 M

Flow rate = 1 mL/min.

Product molecular weight = 200

Product yield = 82 %

$$Productivity\ g/day\ = \frac{0.068 \times 1.0 \times 60 \times 24 \times 200 \times 0.82}{1000}$$

Productivity g/day = 16.058 g/day.

All the products **3a–p** were synthesized following the general procedure as mentioned above.

Ethyl 2-(6-hydroxy-3-oxo-3,6-dihydro-2*H*-pyran-2-yl)acetate (3a)[8]:

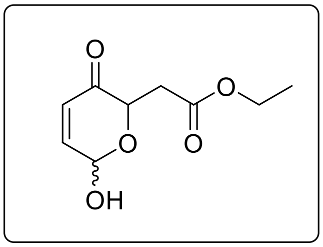

The compound was synthesized by general procedure, purified by column chromatography using hexane and ethyl acetate as mobile phase (hexane/EtOAC, 7:3), to afford **3a** as yellowish oil (89 mg, 82%; d.r. 3:1) as a diastereomeric mixture that were not separated. R_f = 0.5 (hexane/EtOAc, 7:3). IR (neat): υ_{max} 3435.91, 2983.25, 2937.84, 1723.15, 1697.74, 1374.75 1353.86, 1288.14, 1179.65, 1089.67, 1019.80 cm^{-1}. Major diastereomer: ^1H NMR (400 MHz, $CDCl_3$) δ: 6.92 (dd, *J* = 10.3, 3.5 Hz, 1H), 6.15 (d, *J* = 10.3 Hz, 1H), 5.65 (t, *J* = 3.8 Hz, 1H), 5.03 (dd, *J* = 7.6, 3.9 Hz, 1H), 4.17 (qd, *J* = 7.1, 4.6 Hz, 2H), 3.66 (d, *J* = 4.7 Hz, 1H), 3.00 (dd, *J* = 16.8, 3.9 Hz, 1H), 2.75 (dd, *J* = 16.7, 7.6 Hz, 1H), 1.27 (t, *J* = 7.1 Hz, 3H). ^{13}C NMR (101 MHz, $CDCl_3$) δ: 194.95, 170.99, 144.49, 127.35, 87.89, 70.95, 61.15, 35.43, 14.27.ppm. Minor diastereomer: ^1H NMR (400 MHz, $CDCl_3$) δ: 6.96 (dd, *J* = 10.3, 1.6 Hz, 1H), 6.19 (dd, *J* = 10.3, 1.5 Hz, 1H), 5.71 (dd, *J* = 7.4, 1.3 Hz, 1H), 4.59 (ddd, *J* = 7.9, 3.9, 1.2 Hz, 1H), 4.17 (qd, *J* = 7.1, 4.6 Hz, 2H), 3.91 (d, *J* = 7.4 Hz, 1H), 3.01 (dd, *J* = 16.7, 4.0 Hz, 1H), 2.84 – 2.71 (m, 1H), 1.27 (t, *J* = 7.2 Hz, 3H). ^{13}C NMR (100 MHz, $CDCl_3$) δ: 194.48, 170.99, 148.20, 128.49, 90.96, 75.50, 61.28, 36.29, 14.27 ppm. HRMS (ESI) *m/z* $[M+Na]^+$ calcd for $C_9H_{12}NaO_5$: 223.0582 found: 223.0565.

6-Hydroxy-2H-pyran-3(6*H*)-one[9] (3b):

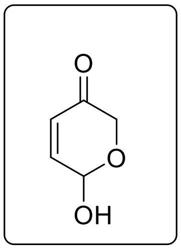

The compound was synthesized by following general procedure, purified by column chromatography using hexane and ethyl acetate as mobile phase (hexane/EtOAC, 2:1), to afford **3b** as light yellow oil (93 mg, 80%) R_f = 0.5 (hexane/EtOAc,2:1). IR (neat): υ_{max} 3281.81, 2938.02, 2887.97, 1668.72, 1268.27, 1089.54, 1040.45, 1008.65, 923.93, 843.75 cm^{-1}. ^{1}H NMR (400 MHz, $CDCl_3$) δ : 6.95 (dd, *J* = 10.4, 3.0 Hz, 1H), 6.16 (d, *J* = 10.4 Hz, 1H), 5.63 (dd, *J* = 4.8, 3.1 Hz, 1H), 4.57 (d, *J* = 16.9 Hz, 1H), 4.13 (d, *J* = 16.9 Hz, 1H), 3.42 (d, *J* = 1.5 Hz, 1H). ^{13}C NMR (100 MHz, $CDCl_3$) δ: 194.66, 145.83, 128.08, 88.37, 66.76 ppm. HRMS (ESI) *m/z* $[M-H]^+$ calcd for $C_5H_5O_3$: 113.0244 found : 113.0252

2-Butyl-6-hydroxy-2*H*-pyran-3(6*H*)-one[2] (3c):

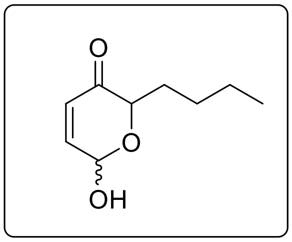

The corresponding compound was prepared following the general procedure, purified by column chromatography using hexane and ethyl acetate as mobile phase (hexane/EtOAC, 1:1), afforded **3c** as colourless oil (93 mg, 85% d.r. 2:1) as a diastereomeric mixture that were not separated. R_f = 0.38 (hexane/EtOAc, 1:1). IR (neat): υ_{max} 3326.11, 2947.56, 2860.00, 1673.96, 1257.01, 1087.49, 1027.38, 975.61 cm^{-1}. Major diastereomer: ^{1}H NMR (400 MHz, $CDCl_3$) δ: 6.89 (dd, *J* = 10.2, 3.4 Hz, 1H), 6.11 (d, *J* =

10.2 Hz, 1H), 5.65 (s, 1H), 4.56 (dd, *J* = 8.1, 3.9 Hz, 1H), 3.09 (brs, 1H), 2.01 – 1.89 (m, 2H), 1.83 – 1.67 (m, 2H), 1.51 – 1.29 (m, 2H), 0.91 (t, *J* = 7.0 Hz, 3H).^{13}C NMR (101 MHz, $CDCl_3$) δ : 196.72, 144.31, 127.71, 87.68, 74.27, 29.37, 27.17, 22.53, 13.98.ppm. Minor diastereomer : ^{1}H NMR (400 MHz, $CDCl_3$) δ: 6.93 (dd, *J* = 10.3, 1.5 Hz, 1H), 6.15 (dd, *J* = 10.3, 1.5 Hz, 1H), 5.67 (s, 1H), 4.08 (ddd, *J* = 8.3, 3.9, 0.9 Hz, 1H), 3.33 (brs, 1H), signals below 2 ppm were indecipherable from major diastereomer. ^{13}C NMR (100 MHz, $CDCl_3$) δ: 196.35, 147.66, 128.82, 90.92, 79.00, 30.38, 27.31, 22.50, 13.98 ppm. HRMS (ESI) *m/z* $[M-H]^+$ calcd for $C_9H_{14}O_3$: 169.0870 found: 169.0873

6-Hydroxy-2,2-dimethyl-2*H*-pyran-3(6*H*)-one[2] (3d):

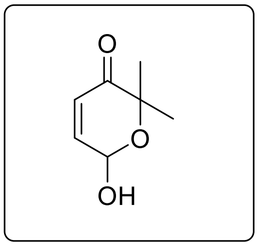

The product **3d** was prepared following the general procedure, purified by column chromatography using hexane and ethyl acetate as mobile phase (hexane/EtOAC, 5:1), to afford **3d** as colourless oil (73 mg, 65%) R_f = 0.3 (hexane/EtOAc, 5:1). IR (neat): $υ_{max}$ 3425.98, 2983.52, 2935.43, 1722.58, 1689.11, 1377.57, 1084.63, 1042.06, 913.97 cm^{-1}. ^{1}H NMR (400 MHz, $CDCl_3$) δ 6.88 (dd, *J* = 10.3, 2.2 Hz, 1H), 6.07 (dd, *J* = 10.3, 1.3 Hz, 1H), 5.69 (d, *J* = 6.3 Hz, 1H), 3.38 (brs, 1H), 1.49 (s, 3H), 1.39 (s, 3H).^{13}C NMR (100 MHz, $CDCl_3$) δ: 199.05, 145.93, 126.56, 87.99, 79.54, 26.67, 23.90 ppm. HRMS (ESI) *m/z* $[M-OH]^+$ calcd for $C_7H_9O_2$: 125.0595 found : 125.0597.

2-Hydroxy-1-oxaspiro[5.5]undec-3-en-5-one[2] (3e):

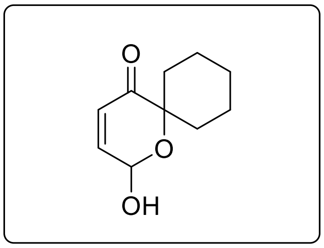

The corresponding compound was prepared following the general procedure, purified by column chromatography using hexane and ethyl acetate as mobile phase (hexane/EtOAC, 5:3), to afford **3e** as a colourless oil (83 mg, 76%) R_f = 0.3 (hexane/EtOAc, 5:3). IR (neat): υ_{max} 3432.98 2932.50, 2858.73, 1719.27, 1684.48, 1446.99, 1262.19, 1116.23, 1025.67, 750.43 cm^{-1}. ^{1}H NMR (400 MHz, $CDCl_3$) δ : 6.84 (dd, *J* = 10.3, 2.0 Hz, 1H), 6.05 (d, *J* = 10.3 Hz, 1H), 5.69 (d, *J* = 5.6 Hz, 1H), 3.45 (brs, 1H), 1.91 (d, *J* = 11.9 Hz, 1H), 1.85 – 1.72 (m, 2H), 1.71 – 1.54 (m, 6H), 1.34 – 1.22 (m, 1H). ^{13}C NMR (100 MHz, $CDCl_3$) δ: 199.29, 145.41, 126.86, 87.59, 80.72, 33.49, 31.06, 25.17, 21.02, 20.59 ppm. HRMS (ESI) *m/z* $[M-OH]^+$ calcd for $C_{10}H_{13}O_2$: 165.0916 found: 165.0942.

6-Hydroxy-2,2-diphenyl-2*H*-pyran-3(6*H*)-one[2] (3f):

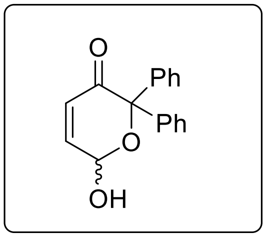

The corresponding compound was prepared following the general procedure, purified by column chromatography using hexane and ethyl acetate as mobile phase (hexane/EtOAC, 5:1), afforded **3f** as colourless oil (79 mg, 75%) R_f = 0.4 (hexane/EtOAc, 5:1). IR (neat): υ_{max} 3625.23, 3003.11, 2944.95, 2252.35, 1442.16, 1376.71, 1038.73, 918.74, 748.87 cm^{-1}. ^{1}H NMR (500 MHz, $CDCl_3$) δ: 7.40 – 7.32 (m, 5H), 7.30 – 7.26 (m,

5H), 6.90 (dd, J = 10.3, 1.4 Hz, 1H), 6.31 (dd, J = 10.3, 1.6 Hz, 1H), 5.50 (d, J = 8.3 Hz, 1H), 3.07 (d, J = 8.4 Hz, 1H). ^{13}C NMR (100 MHz, $CDCl_3$) δ: 194.44, 147.53, 141.40, 137.50, 129.14, 128.76, 128.72, 128.02, 127.89, 127.73, 127.64, 88.99, 87.08 ppm. HRMS (ESI) *m/z* $[M\text{-}OH]^+$ calcd for $C_{17}H_{13}O_2$: 249.0910found: 249.0910

6-Hydroxy-2-(4-isopropylphenyl)-2*H*-pyran-3(6*H*)-one (3g)[3]:

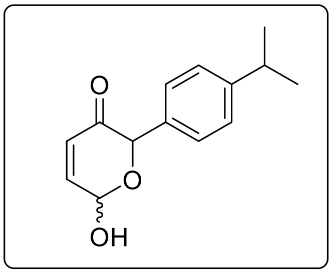

The corresponding compound was prepared following the general procedure, purified by column chromatography using hexane and ethyl acetate as mobile phase (hexane/EtOAC, 7:3), afforded **3g** as a white solid (83 mg, 78%; d.r. 3:1) as a diastereomeric mixture that were not separated. R_f = 0.5 (hexane/EtOAc, 7:3). IR (neat): $υ_{max}$ 3428.58 2961.47, 2927.87, 2874.26, 1722.25, 1178.61, 1100.25, 1053.62, 1014.72, 828.18,755.20 cm^{-1}. Major diastereomer: ^{1}H NMR (400 MHz, $CDCl_3$) δ: 7.30 – 7.22 (m, 4H), 6.93 (dd, J = 10.3, 3.3 Hz, 1H), 6.20 (dd, J = 10.3, 0.7 Hz, 1H), 5.68 (d, J = 2.4 Hz, 1H), 5.55 (s, 1H), 3.66 (s, 1H), 2.94 – 2.87 (m, 1H), 1.25 (s, 3H), 1.23 (s, 3H). ^{13}C NMR (101 MHz, $CDCl_3$) δ: 194.98, 149.47, 145.00, 132.72, 129.47, 128.12, 126.76, 88.15, 77.12, 34.02, 24.02 ppm. Minor diastereomer: ^{1}H NMR (400 MHz, $CDCl_3$) δ: 7.30 – 7.22 (m, 4H), 6.98 (dd, J = 10.3, 1.3 Hz, 1H), 6.25 (dd, J = 10.3, 1.6 Hz, 1H), 5.74 (brs, 1H), 5.05 (brs, 1H), 3.85 (brs, 1H), 2.94 – 2.87 (m, 1H), 1.25 (s, 3H), 1.23 (s, 3H). ^{13}C NMR (100 MHz, $CDCl_3$) δ: 194.64, 149.54, 148.39, 132.97, 129.47, 128.05, 127.98, 91.58,

81.20, 34.02, 24.04 ppm. HRMS (ESI) *m/z* $[M+OH]^+$ calcd for $C_{14}H_{15}O_2$: 215.1064 found: 215.1066.

Methyl 4-(6-hydroxy-3-oxo-3,6-dihydro-2*H*-pyran-2-yl)benzoate[4] (3h):

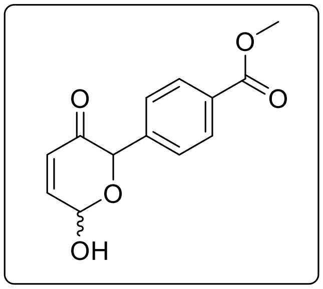

The corresponding compound was prepared following the general procedure, purified by column chromatography using hexane and ethyl acetate as mobile phase (hexane/EtOAC, 7:3), afforded **3h** as a white solid (80 mg, 75% d.r 3:1) as a diastereomeric mixture that were not separated. MP = 144-147 ^{o}C, R_f = 0.39 (hexane/EtOAc, 7:3). IR (neat): υ_{max} 3410.75, 2920.99, 1692.25, 1287.21, 1092.71, 1027.17, 759.22 cm^{-1}. Major diosteriomer:^{1}H NMR (400 MHz, $CDCl_3$) δ: 8.04 (d, *J* = 8.3 Hz, 2H), 7.44 (d, *J* = 8.3 Hz, 2H), 7.01 (dd, *J* = 10.3, 3.4 Hz, 1H), 6.22 (d, *J* = 10.3 Hz, 1H), 5.81 (s, 1H), 5.66 (s, 1H), 3.92 (s, 3H), 3.42 (s, 1H). ^{13}C NMR (100 MHz, $CDCl_3$) δ: 193.63, 167.11, 144.87, 140.26, 130.21, 129.72, 129.34, 127.92, 88.23, 76.29, 52.37. ^{1}H NMR (400 MHz, $CDCl_3$) δ: 8.04 (d, *J* = 8.3 Hz, 2H), 7.47 d, *J* = 8.3 Hz, 2H), 7.06 (dd, *J* = 10.3, 1.3 Hz, 1H), 6.28 (dd, *J* = 10.3, 1.4 Hz, 1H), 5.86 (brs, 1H), 5.17 (brs, 1H), 3.96 (s, 3H), 3.65 (s, 1H). ^{13}C NMR (100 MHz, $CDCl_3$) δ: 193.22, 167.11, 148.51, 140.26, 130.21, 129.72, 129.34, 127.77, 91.68, 80.45, 52.37 ppm. HRMS (ESI) *m/z* $[M+H]^+$ calcd for $C_{13}H_{13}O_5$: 249.0753 found: 249.0757

2-(3-Fluorophenyl)-6-hydroxy-2*H*-pyran-3(6*H*)-one (3i):

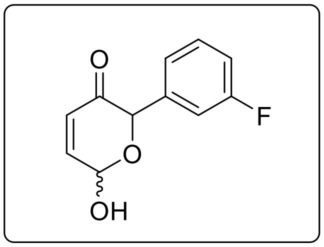

The compound was synthesized by general procedure, purified by column chromatography using hexane and ethyl acetate as mobile phase (hexane/EtOAC, 7:3), to afford **3i** as colourless oil (77 mg, 72% d.r 7:2) as a diastereomeric mixture that were not separated. R_f = 0.3 (hexane/EtOAc, 7:3). IR (neat): υ_{max} 3418.58, 1692.30, 1598.80, 1248.46, 1028.26, 783.79, 689.70 cm^{-1}. Major diastereomer: ^1H NMR (400 MHz, $CDCl_3$) δ: 7.34 (td, *J* = 8.0, 5.9 Hz, 1H), 7.19 – 7.11 (m, 1H), 7.11 – 7.03 (m, 2H), 6.98 (dd, *J* = 10.3, 3.4 Hz, 1H), 6.21 (dd, *J* = 10.3, 0.6 Hz, 1H), 5.77 (s, 1H), 5.59 (s, 1H), 3.36 (brs, 1H). ^{13}C NMR (100 MHz, $CDCl_3$) δ: 193.74, 163.97, 161.52, 144.81, 129.95, 129.87, 127.67, 123.68, 123.65, 115.61, 115.40, 114.92, 114.70, 88.09, 76.11 ppm. ^{19}F NMR (376 MHz, $CDCl_3$) δ: -112.74 (s). Minor diastereomer: ^1H NMR (400 MHz, $CDCl_3$) δ: 7.34 (td, *J* = 8.0, 5.9 Hz, 1H), 7.19 – 7.11 (m, 1H), 7.11 – 7.03 (m, 2H), 6.98 (dd, *J* = 10.3, 3.4 Hz, 1H), 6.27 (dd, *J* = 10.3, 1.6 Hz, 1H), 5.82 (d, *J* = 2.7 Hz, 1H), 5.11 (s, 1H), 3.55 (brs, 1H). ^{13}C NMR (101 MHz, $CDCl_3$) δ: 193.84, 193.45, 162.84 (d, $J_{C\text{-}Fmajor}$ = 246.1 Hz), 148.40, 144.91, 137.62 (d, $J_{C\text{-}Fmajor}$ = 7.4 Hz), 130.01 (d, $J_{C\text{-}Fmajor}$ = 8.09 Hz), 129.33, 127.78, 123.76 (d, $J_{C\text{-}Fmajor}$ = 2.4 Hz), 123.59, 115.64, 115.60 (d, $J_{C\text{-}Fmajor}$ = 21.0 Hz), 114.91 (d, $J_{C\text{-}Fmajor}$ = 22.6 Hz),114.83, 91.56, 88.19, 80.29, 76.21.^{19}F NMR (376 MHz, $CDCl_3$) δ: -112.69 (s). HRMS (ESI) m/z $[M\text{-}H]^+$ calcd for $C_{11}H_8FO_3$: 207.0463 found: 207.0458.

2-(2-Chlorophenyl)-6-hydroxy-2*H*-pyran-3(6*H*)-one (3j)[4]:

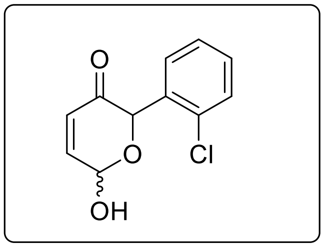

The compound was synthesized by general procedure, purified by column chromatography using hexane and ethyl acetate as mobile phase (hexane/EtOAC, 7:3), afforded **3j** as colourless oil (86 mg, 80%; d.r. 7:3) as a diastereomeric mixture that were not separated. R_f = 0.28 (hexane/EtOAc, 1:1). IR (neat): υ_{max} 3395.33, 1691.12, 1222.18, 1080.47, 1023.12,756.97 cm^{-1}. Major diastereomer: ^1H NMR (400 MHz, $CDCl_3$) δ: 7.39 (dd, *J* = 3.1, 2.1 Hz, 1H), 7.32 (dd, *J* = 11.8, 2.5 Hz, 1H), 7.28 (dd, *J* = 4.7, 2.2 Hz, 2H), 6.90 (dd, *J* = 10.3, 3.5 Hz, 1H), 6.18 (d, *J* = 10.3 Hz, 1H), 5.99 (s, 1H), 5.68 (s, 1H), 4.06 (s, 1H).^{13}C NMR (100 MHz, $CDCl_3$) δ: 193.8, 144.2, 134.5, 133.8, 130.1, 129.94, 129.86, 127.8, 127.1, 88.4, 74.3 ppm. Minor diastereomer: ^1H NMR (400 MHz, $CDCl_3$) δ 7.42 – 7.38 (m, 5H), 7.35 – 7.32 (m, 3H), 7.31 – 7.23 (m, 9H), 6.93 (dd, *J* = 10.2, 1.3 Hz, 2H), 6.22 (dd, *J* = 10.3, 1.7 Hz, 1H), 5.74 (s, 1H), 5.49 (d, *J* = 1.5 Hz, 1H), 4.38 (s, 1H). ^{13}C NMR (100 MHz, $CDCl_3$) δ: 193.30, 148.39, 144.35, 134.04, 133.52, 129.59, 129.50, 129.32, 127.59, 127.10, 92.04, 78.36 ppm. HRMS (ESI) *m/z* $[M-H]^+$ calcd for $C_{11}H_8ClO_3$: 223.0167 found: 223.0166.

2-(2-Bromophenyl)-6-hydroxy-2*H*-pyran-3(6*H*)-one (3k):

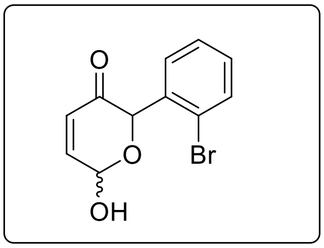

The compound was synthesized by general procedure, purified by column chromatography using hexane and ethyl acetate as mobile phase (hexane/EtOAc, 7:3), afforded **3k** as colourless oil (86 mg,81%; d.r. 7:3) as a diastereomeric mixture that were not separated. R_f = 0.4 (hexane/EtOAc, 7:3). IR (neat): υ_{max} 3393.35, 1689.02, 1220.05, 1081.36, 1022.22,756.86 cm^{-1}. Major diastereomer: ^1H NMR (400 MHz, $CDCl_3$) δ: 7.60 (dd, *J* = 7.9, 0.9 Hz, 1H), 7.34 (td, *J* = 7.8, 1.6 Hz, 2H), 7.22 (dd, *J* = 7.4, 1.6 Hz, 1H), 6.94 (dd, *J* = 10.3, 3.4 Hz, 1H), 6.21 (d, *J* = 10.3 Hz, 1H), 6.02 (s, 1H), 5.74 (d, *J* = 2.8 Hz, 1H), 3.61 (s, 1H). ^{13}C NMR (126 MHz, $CDCl_3$) δ: 193.63, 148.22, 144.17, 135.47, 133.04, 130.28, 130.01, 127.75, 124.66, 88.35, 76.13. Minor diastereomer: ^1H NMR (400 MHz, $CDCl_3$) δ: 7.59 (dd, *J* = 7.9, 0.9 Hz, 8H), 7.43 – 7.29 (m, 17H), 7.24 – 7.18 (m, 8H), 6.99 (d, *J* = 0.6 Hz, 2H), 6.27 (dd, *J* = 10.3, 1.3 Hz, 2H), 5.82 (s, 2H), 5.54 (brs, 2H), 3.87 (brs, 2H). ^{13}C NMR (126 MHz, $CDCl_3$) δ: 193.08, 148.22, 144.17, 135.24, 132.84, 129.65, 129.45, 127.63, 124.30, 92.09, 80.39 ppm. HRMS (ESI) *m/z* $[M-OH]^+$ calcd for $C_{11}H_8BrO_2$: 250.9698 found: 250.9702

6-Hydroxy-2-(2-(trifluoromethyl)phenyl)-2*H*-pyran-3(6*H*)-one (3l):

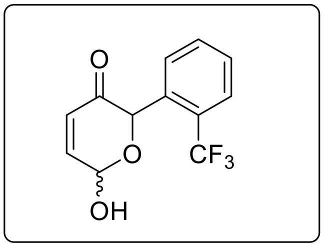

The compound was synthesized by general procedure, purified by column chromatography using hexane and ethyl acetate as mobile phase (hexane/EtOAC, 1:1), afforded **3l** as colourless oil, (83 mg, 78%; d.r. 7:3) as a diastereomeric mixture that were not separated. R_f = 0.28 (hexane/EtOAc, 1:1). IR (neat): υ_{max} 3410.56, 1695.19, 1309.94,

1159.66, 1115.61, 1027.45, 767.11 cm^{-1}. Major diastereomer: ^1H NMR (400 MHz, $CDCl_3$) δ 7.70 (d, *J* = 7.8 Hz, 1H), 7.59 – 7.53 (m, 1H), 7.46 (t, *J* = 7.7 Hz, 2H), 6.93 (dd, *J* = 10.3, 3.5 Hz, 1H), 6.18 (d, *J* = 10.4 Hz, 1H), 6.04 (s, 1H), 5.69 (s, 1H), 3.71 (brs, 1H). ^{13}C NMR (101 MHz, $CDCl_3$) δ 194.36, 144.47, 134.38, 132.20, 130.41, 129.39, 128.95, 127.58, 126.11, 126.06, 126.01, 125.95, 122.78, 88.42, 77.48 ppm. ^{19}F NMR (376 MHz, $CDCl_3$) δ: -58.20 (s). Minor diastereomer: ^1H NMR (400 MHz, $CDCl_3$) δ 7.70 (d, *J* = 7.8 Hz, 1H), 7.59 – 7.53 (m, 1H), 7.46 (t, *J* = 7.7 Hz, 2H), 6.96 (dd, *J* = 10.3, 1.2 Hz, 1H), 6.25 (dd, *J* = 10.3, 1.7 Hz, 1H), 5.80 (s, 1H), 5.47 (d, *J* = 1.3 Hz, 1H), 3.99 (brs, 1H). ^{13}C NMR (100 MHz, $CDCl_3$) δ 194.36, 193.69, 148.54, 144.47, 134.38, 134.09, 132.32, 132.20, 130.41, 129.89, 129.65, 129.39, 129.07, 128.95, 127.58, 126.0 (q, $J_{C\text{-}Fmajor}$ = 5.4 Hz), 124.1 (q, $J_{C\text{-}Fmajor}$ = 273 Hz), 92.20, 88.42, 77.48, 72.85 ppm. ^{19}F NMR (376 MHz, $CDCl_3$) δ -57.90 (s). HRMS (ESI) *m/z* $[M\text{-}OH]^+$ calcd for $C_{12}H_8F_3O_2$: 241.0476 found: 241.0452.

6-Hydroxy-2-(2-nitrophenyl)-2*H*-pyran-3(6*H*)-one (3m):

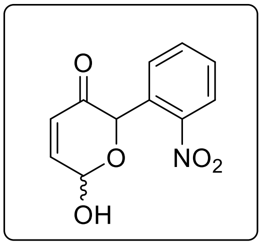

The compound was synthesized by general procedure, purified by column chromatography using hexane and ethyl acetate as mobile phase (hexane/EtOAc, 1:1), afforded **3m** as brown solid (54 mg, 51% d.r 2.5:1) as a diastereomeric mixture that were not separated.MP = 134-137 ^oC, R_f = 0.27 (hexane/EtOAc, 1:1). IR (neat): $υ_{max}$ 3357.09, 3102.98, 2921.96, 2852.73, 1682.95, 1522.16, 1338.68, 1211.26, 1082.12, 1020.90, 731.63 cm^{-1}. Major diastereomer: ^1H NMR (400 MHz, $CDCl_3$) δ 8.05 (d, *J* = 8.1 Hz, 1H), 7.68 – 7.63 (m, 2H), 7.52 (ddd, *J* = 6.0, 4.3, 2.3 Hz, 1H), 6.95 (dd, *J* = 10.3, 3.5 Hz, 1H),

6.36 (s, 1H), 6.24 (d, *J* = 10.3 Hz, 1H), 5.81 (d, *J* = 3.3 Hz, 1H), 3.18 (brs, 1H). ^{13}C NMR (126 MHz, $CDCl_3$) δ: 191.86, 147.79, 143.56, 133.29, 130.05, 129.45, 128.63, 127.50, 125.15, 92.64, 88.65 ppm. Minor diastereomer: ^{1}H NMR (400 MHz, $CDCl_3$) δ 8.10 (dd, *J* = 8.2, 1.1 Hz, 1H), 7.76 (m, 2H), 7.52 (ddd, *J* = 6.0, 4.3, 2.3 Hz, 1H), 7.01 (dd, *J* = 10.3, 1.1 Hz, 1H), 6.29 (dd, *J* = 10.3, 1.7 Hz, 1H), 6.09 (s, 1H), 5.93 (s, 1H), 3.46 (brs, 1H). ^{13}C NMR (126 MHz, $CDCl_3$) δ 191.86, 148.67, 143.56, 133.55, 130.38, 129.50, 129.31, 128.63, 125.03,92.64, 77.29 ppm. HRMS (ESI) *m/z* $[M-OH]^+$ calcd for $C_{11}H_8NO_4$: 218.0448 found: 218.0420

2-(3,4-Dimethoxyphenyl)-6-hydroxy-2*H*-pyran-3(6*H*)-one[10] (3n):

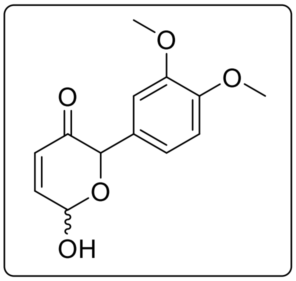

The compound was synthesized by general procedure, purified by column chromatography using hexane and ethyl acetate as mobile phase (hexane/EtOAc, 1:1), afforded **3n** as colourless oil (79 mg, 74% d.r. 3:1) as a diastereomeric mixture that were not separated. R_f = 0.27 (hexane/EtOAc, 1:1). IR (neat): $υ_{max}$ 3438.78 2936.77, 2845.72, 1690.59, 1515.71, 1258.48, 1143.76, 1023.54, 757.44 cm^{-1}. Major diastereomer: ^{1}H NMR (400 MHz, $CDCl_3$) δ 6.98 (dd, *J* = 10.3, 3.3 Hz, 1H), 6.86 (m, 2H), 6.22 (dd, *J* = 10.3, 0.6 Hz, 1H), 6.18 (d, *J* = 5.8 Hz, 1H), 5.74 (d, *J* = 3.3 Hz, 1H), 5.54 (s, 1H), 3.87 (s, 3H), 3.87 (s, 3H). ^{13}C NMR (126 MHz, $CDCl_3$) δ 195.05, 149.36, 149.08, 145.09, 129.44, 127.89, 120.94, 111.05, 111.00, 98.64, 91.63, 88.19, 56.02 ppm. Minor diastereomer: ^{1}H NMR (400 MHz, $CDCl_3$) δ 7.02 (dd, *J* = 10.3, 1.4 Hz, 1H), 6.91 (m, 2H), 6.28 (dd, *J* = 10.3, 1.6

Hz, 1H), 6.15 (s, 1H), 5.81 (d, J = 1.4 Hz, 1H), 5.05 (s, 1H), 3.94 (s, 3H), 3.93 (s, 3H). ^{13}C NMR (126 MHz, $CDCl_3$) δ 194.58, 169.53, 151.78, 149.28, 148.40, 129.37, 128.05, 124.60, 112.33, 98.56, 91.55, 81.08, 55.92 ppm. HRMS (ESI) m/z $[M\text{-}OH]^+$ calcd for $C_{13}H_{13}O_4$: 233.0808 found : 233.0779.

6-Hydroxy-6-methyl-2*H*-pyran-3(6*H*)-one[2] (3o):

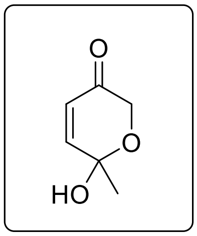

The compound was synthesized by general procedure, purified by column chromatography using hexane and ethyl acetate as mobile phase (hexane/EtOAc, 1:1), afforded **3o** as colorless oil (90 mg, 79%), R_f = 0.27 (hexane/EtOAc, 1:1). IR (neat): $υ_{max}$ 3374.49, 2990.46, 2887.76. 1682.56, 1376.17, 1270.57, 1126.77, 1079.21, 918.02, 861.03, 781.80 cm^{-1}. ^{1}H NMR (400 MHz, $CDCl_3$) δ 6.87 (d, J = 10.3 Hz, 1H), 6.07 (d, J = 10.3 Hz, 1H), 4.57 (d, J = 16.9 Hz, 1H), 4.12 (d, J = 16.9 Hz, 1H), 2.78 (s, 1H), 1.64 (s, 3H). ^{13}C NMR (126 MHz, $CDCl_3$) δ 194.81, 148.79, 126.73, 92.98, 66.74, 28.15 ppm. HRMS (ESI) m/z $[M]^+$ calcd for $C_6H_8O_3$: 128.0435 found: 128.0439.

6-Hydroxy-2,6-dimethyl-2*H*-pyran-3(6*H*)-one[11] (3p):

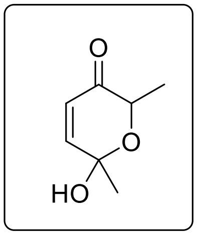

The compound was synthesized by general procedure, purified by column chromatography using hexane and ethyl acetate as mobile phase (hexane/EtOAC, 2:1), afforded **3p** as light yellow oil (93 mg, 80% d.r. 8:2) as a diastereomeric mixture that were not separated. IR (neat): υ_{max} 3394.04, 2990.16, 2938.93, 2867.44, 1688.66, 1371.63, 1241.70, 1122.79, 1085.73, 1036.97, 926.44, 846.55 cm^{-1}. Major diastereomer: ^{1}H NMR (400 MHz, $CDCl_3$) δ: 6.81 (d, *J* = 10.1 Hz, 1H), 6.02 (d, *J* = 10.1 Hz, 1H), 4.65 (q, *J* = 6.7 Hz, 1H), 2.66 (brs, 1H), 1.64 (d, *J* = 1.1 Hz, 3H), 1.37 (d, *J* = 6.8 Hz, 3H). ^{13}C NMR (100 MHz, $CDCl_3$) δ 197.09, 147.81, 126.39, 93.03, 70.89, 29.21, 15.46 ppm. Minor diastereomer: ^{1}H NMR (400 MHz, $CDCl_3$) δ: 6.87 (d, *J* = 10.3 Hz, 1H), 4.31 (q, *J* = 6.8 Hz, 1H), 2.79 (d, *J* = 105.3 Hz, 1H), 1.62 (d, *J* = 0.8 Hz, 3H), 1.46 (d, *J* = 6.8 Hz, 3H). ^{13}C NMR (100 MHz, $CDCl_3$) δ 197.09, 147.81, 126.39, 93.03, 70.89, 29.21, 15.46 ppm. HRMS (ESI) *m/z* $[M-OH]^{+}$ calcd for $C_7H_9O_2$: 125.0595 found: 125.0597.

References:

1) Srihari, P.; Sridhar, Y. *Eur. J. Org. Chem*. **2011**, *21*, 6690–6697.
2) Xing, Q.; Hao, Z.; Hou, J.; Li, G.; Gao, Z.; Gou, J.; Li, C.; Yu, B. *J. Org. Chem*. **2021**, 9563–9586.
3) Zhao, C.; Wang, J. *Adv. Synth. Catal*. **2019,** *361*, 1668-1672.
4) Plutschack, M.B.; Seeberger, P.H.; Gilmore, K. *Org. Lett.* **2017**, *19*, 30–33.
5) Tang, M.; Kong, Y.; Chu, B.; Feng, D. *Adv. Synth. Catal*. **2016,** *358*, 926–939.
6) Li, H.; Tong, R.; Sun, J. *Angew. Chem. Int Ed*. *2016*, *55,* 15125–15128.
7) Qin, S.; Li, T.; Zhang, M.; Liu, H.; Yang, X.; Rong, N.; Jiang, J.; Wang, Y.; Zhang, H.; Yang, W. *Green Chem,* **2019**, *21*, 6326–6334.
8) Li, Z.; Tong, R. *J. Org. Chem*, **2016**, *81*, 4847-4855.
9) Wei, C.; Zhao, R.; Shen, Z.; Chang, D.; Shi, L. *Org. Biomol. Chem,* **2018**,*16*, 5566–5569.
10) Georgiadis, M. P.; Haroutounian, S. A.; Couladouros, E. A.; Apostolopoulos, C. D.; Chondros, K. P. *J. Heterocycl. Chem*. **1991**, *28* 697–703.
11) Marshall, K. A.; Mapp, A. K.; Heathcock, C. H. *J. Org. Chem.* **1996**, *61*, 9135–9145.
12) Solanke, P. R.; Kumar, P.; Mainkar, P.S.; Nayani, K.; Chandrasekhar, S. *Chem. Commun.* **2024**, *60*, 4234–4237.
13) Dhokale, R. A.; Mhaske, S. B. *Org Lett.* **2016**, *18,* 3010–3013.

NMR-spectra

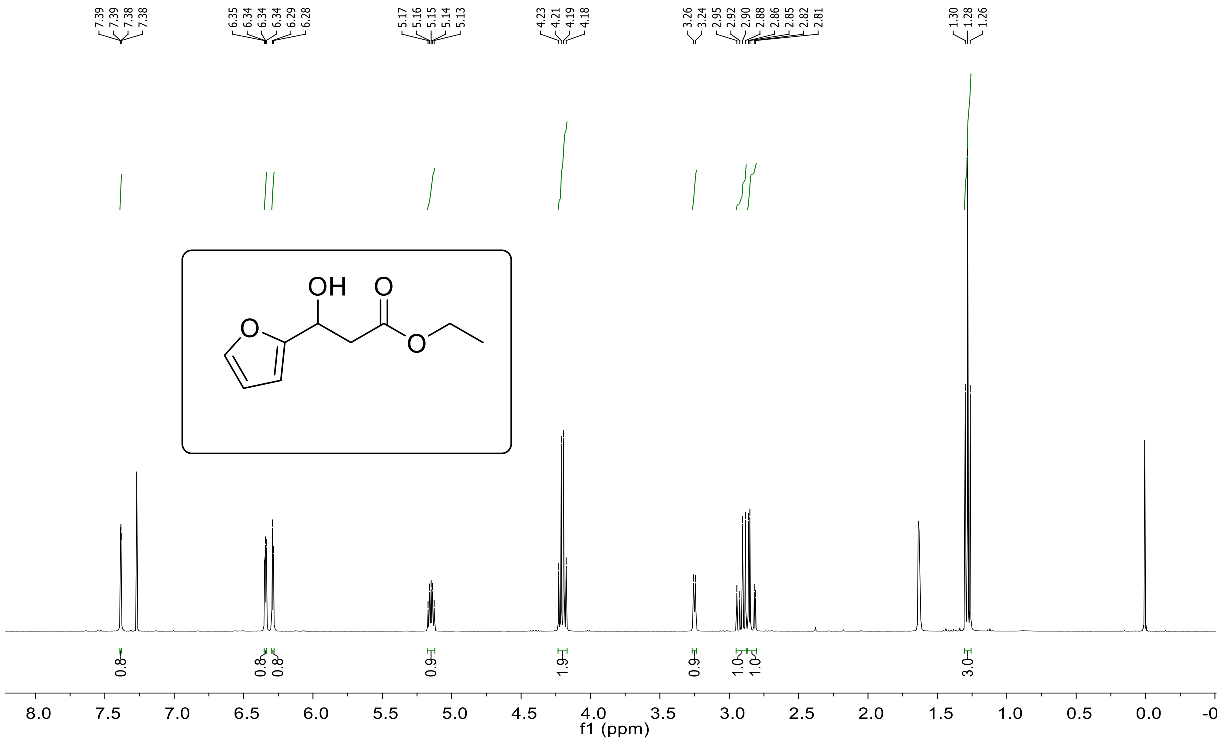

^{1}H NMR (400 MHz, $CDCl_3$) spectrum of ethyl 3-(furan-2-yl)-3-hydroxypropanoate (**2a**)

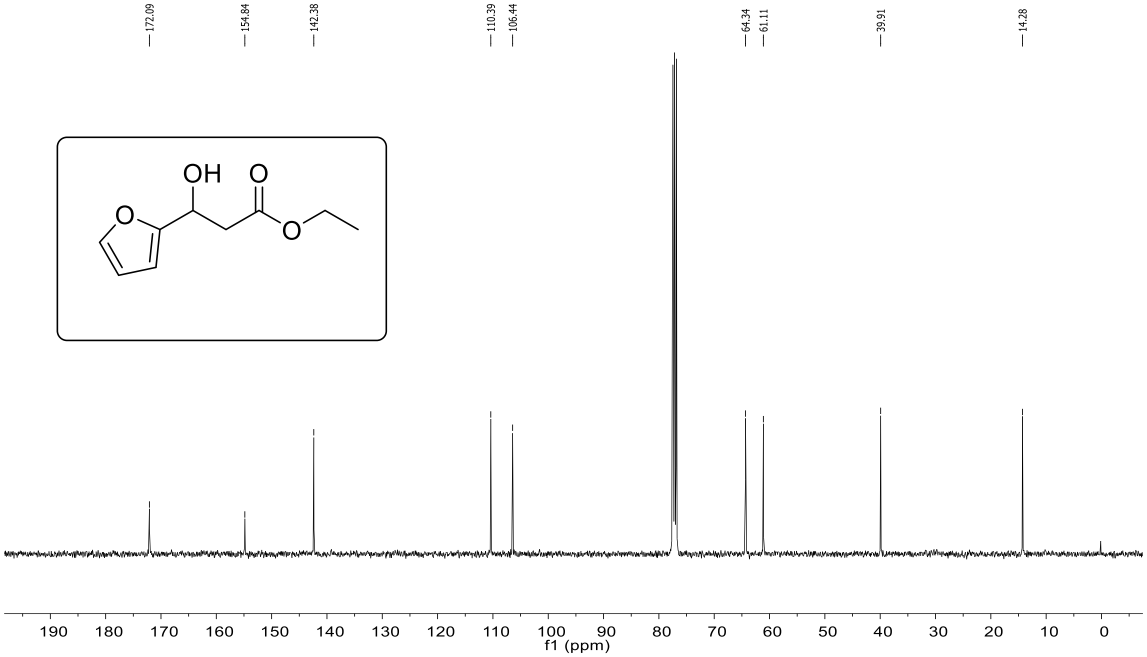

^{13}C NMR (100 MHz, $CDCl_3$) spectrum of ethyl 3-(furan-2-yl)-3-hydroxypropanoate (**2a**)

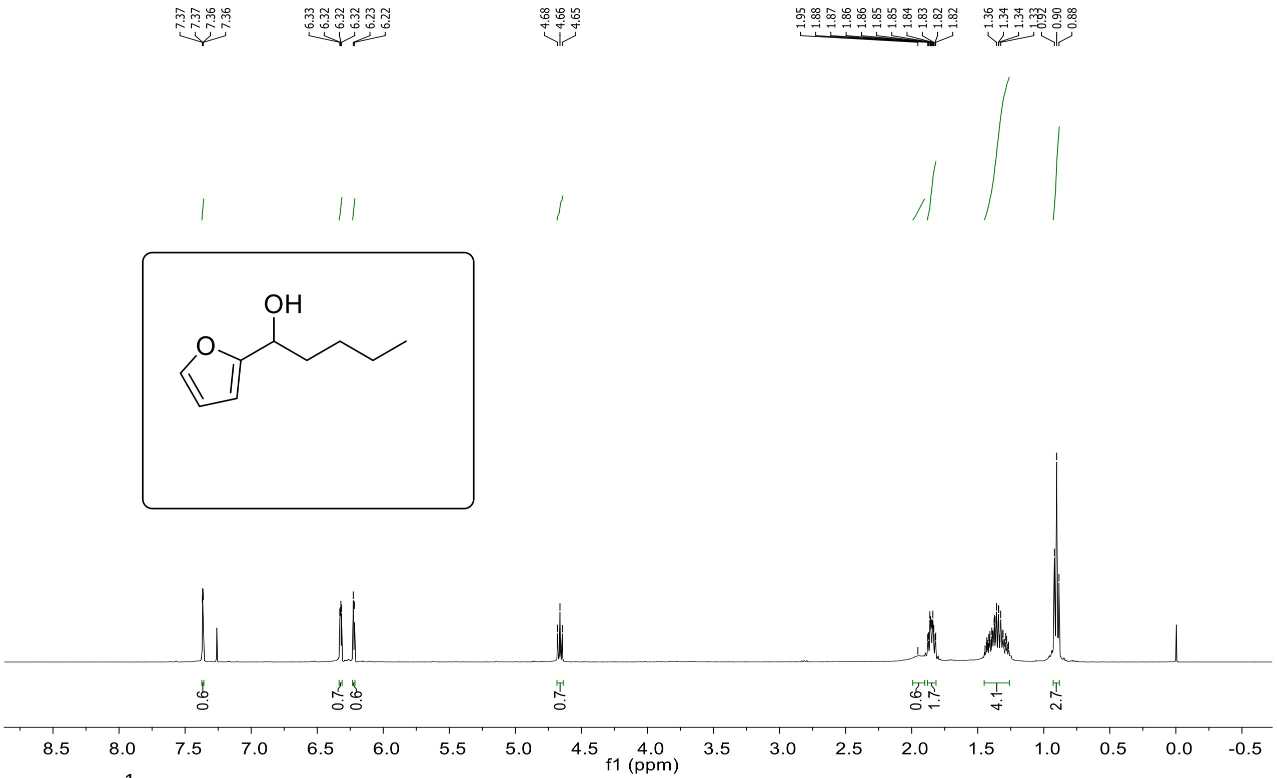

^{1}H NMR (400 MHz, $CDCl_3$) spectrum of 1-(furan-2-yl)pentan-1-ol (**2c**)

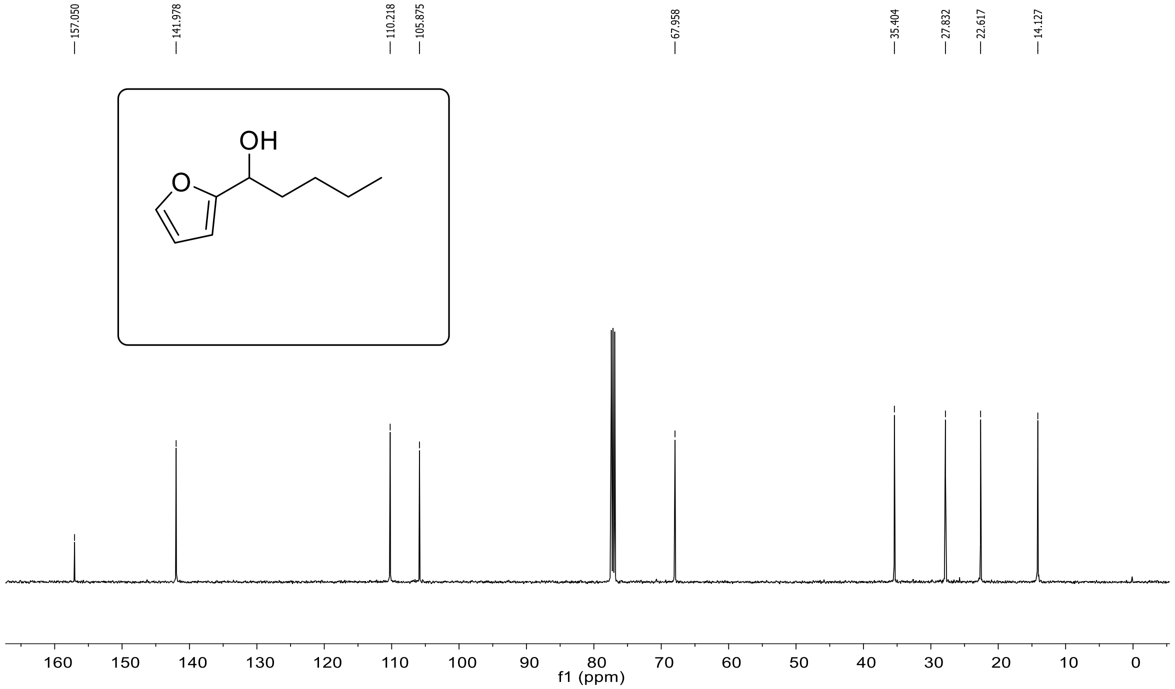

^{13}C NMR (125 MHz, $CDCl_3$) spectrum of 1-(furan-2-yl)pentan-1-ol (**2c**)

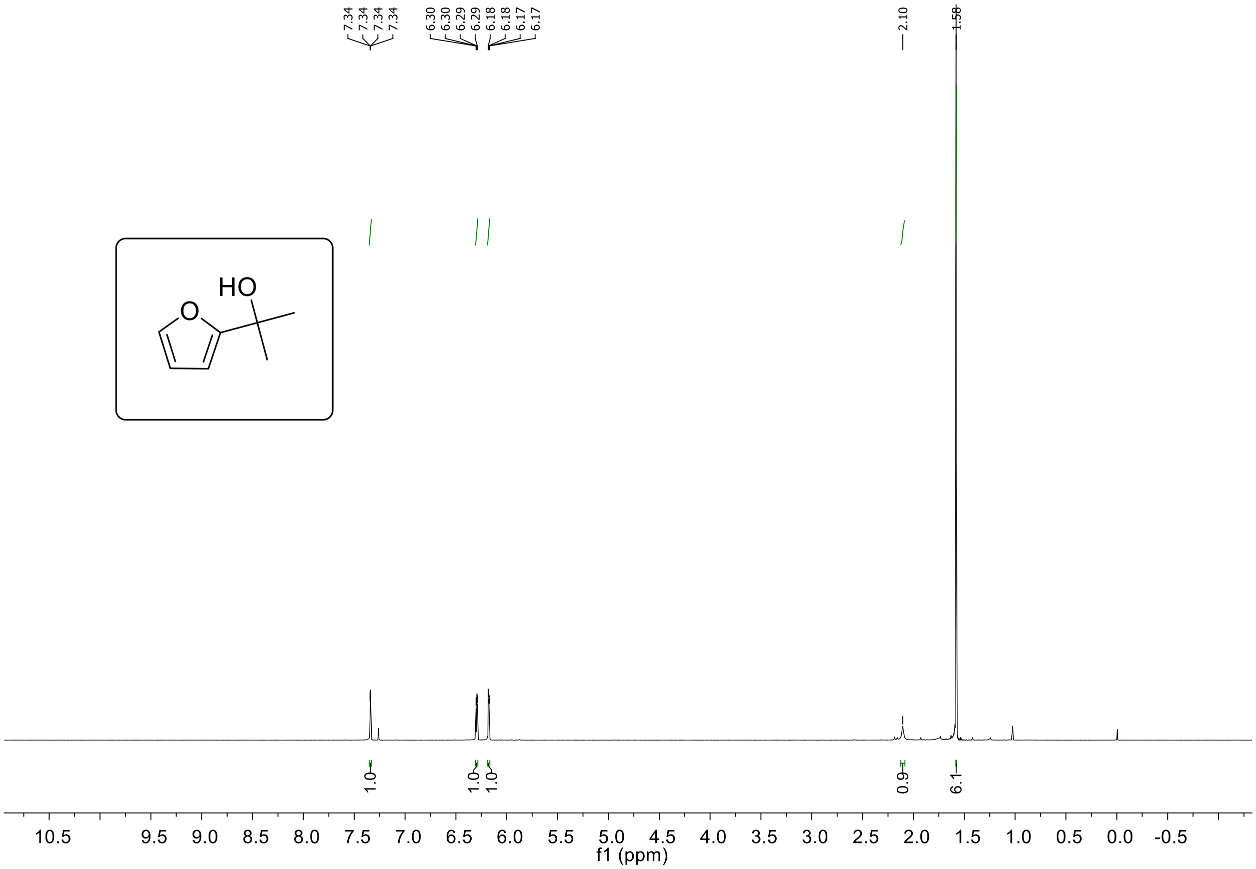

^1H NMR (400 MHz, $CDCl_3$) spectrum of 2-(furan-2-yl)propan-2-ol (**2d**)

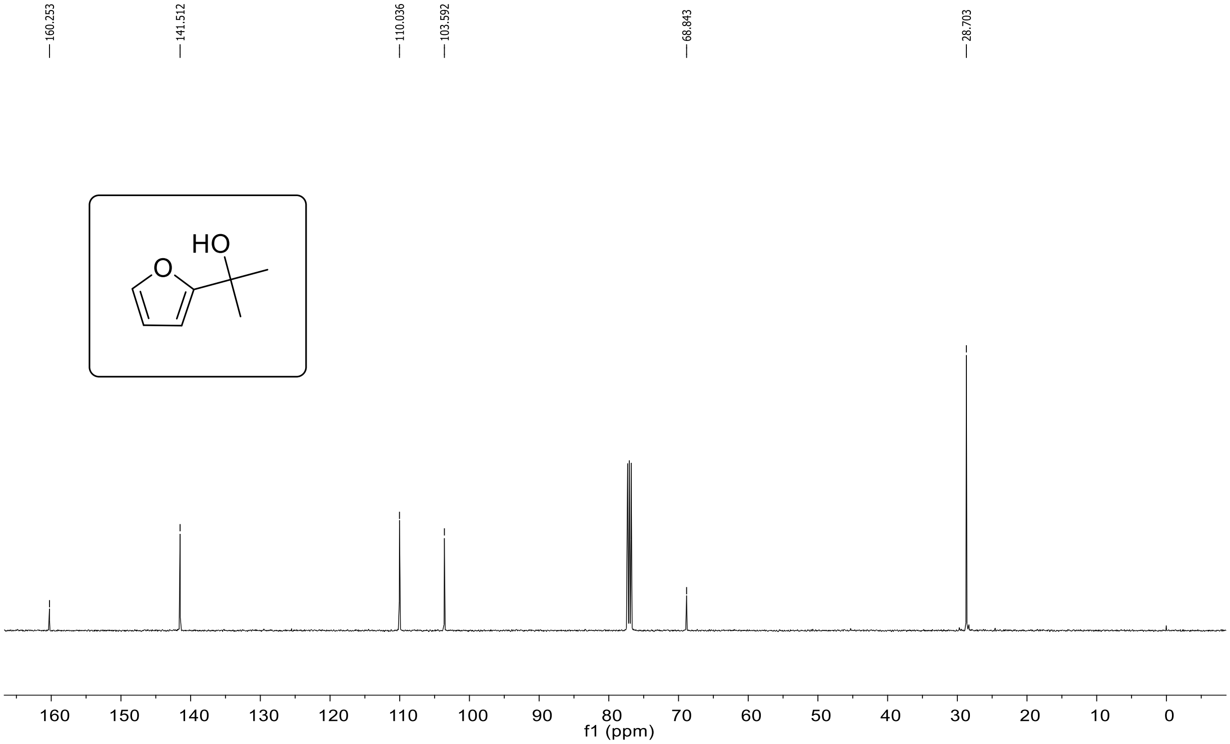

^{13}C NMR (125 MHz, $CDCl_3$) spectrum of 2-(furan-2-yl)propan-2-ol (**2d**)

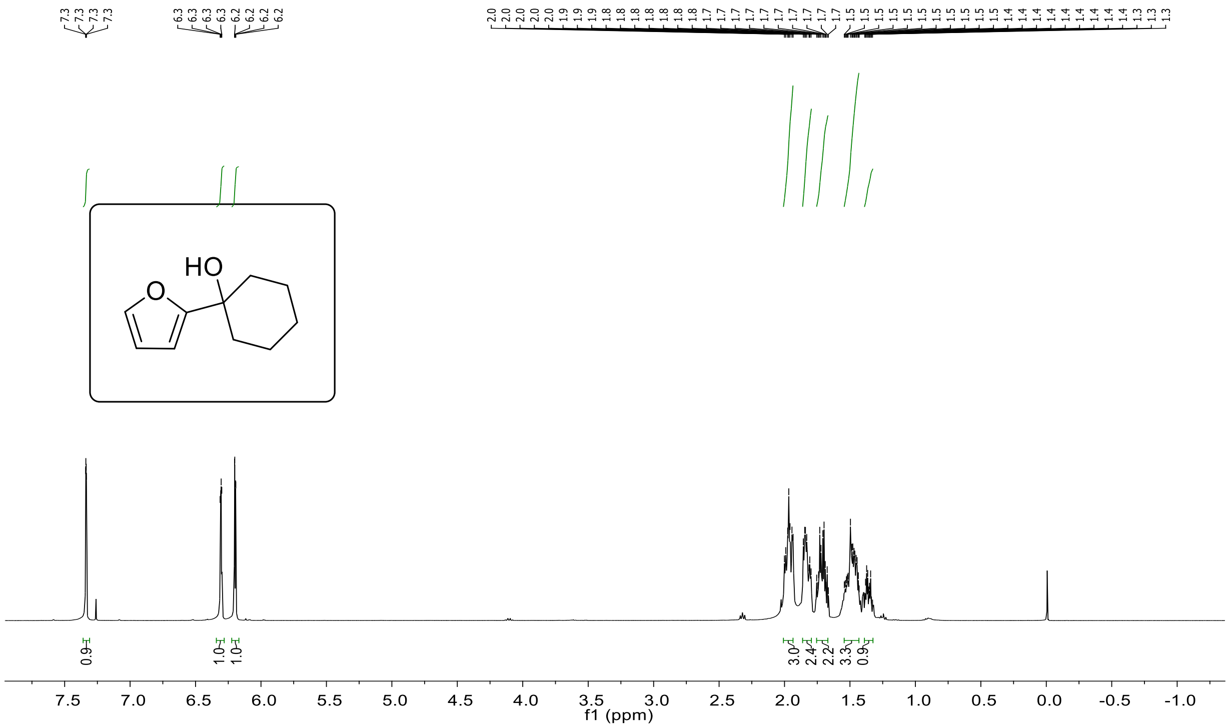

^{1}H NMR (400 MHz, $CDCl_3$) spectrum of 1-(furan-2-yl)cyclohexan-1-ol (**2e**)

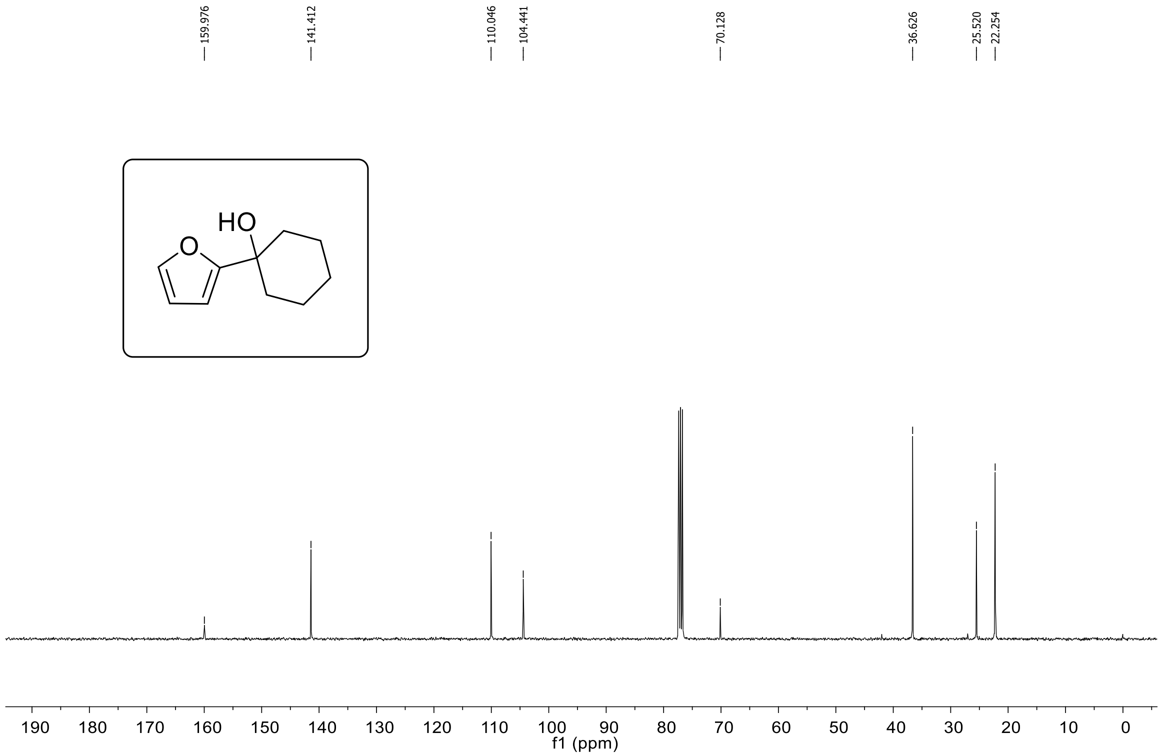

^{13}C NMR (100 MHz, $CDCl_3$) spectrum of 1-(furan-2-yl)cyclohexan-1-ol (**2e**)

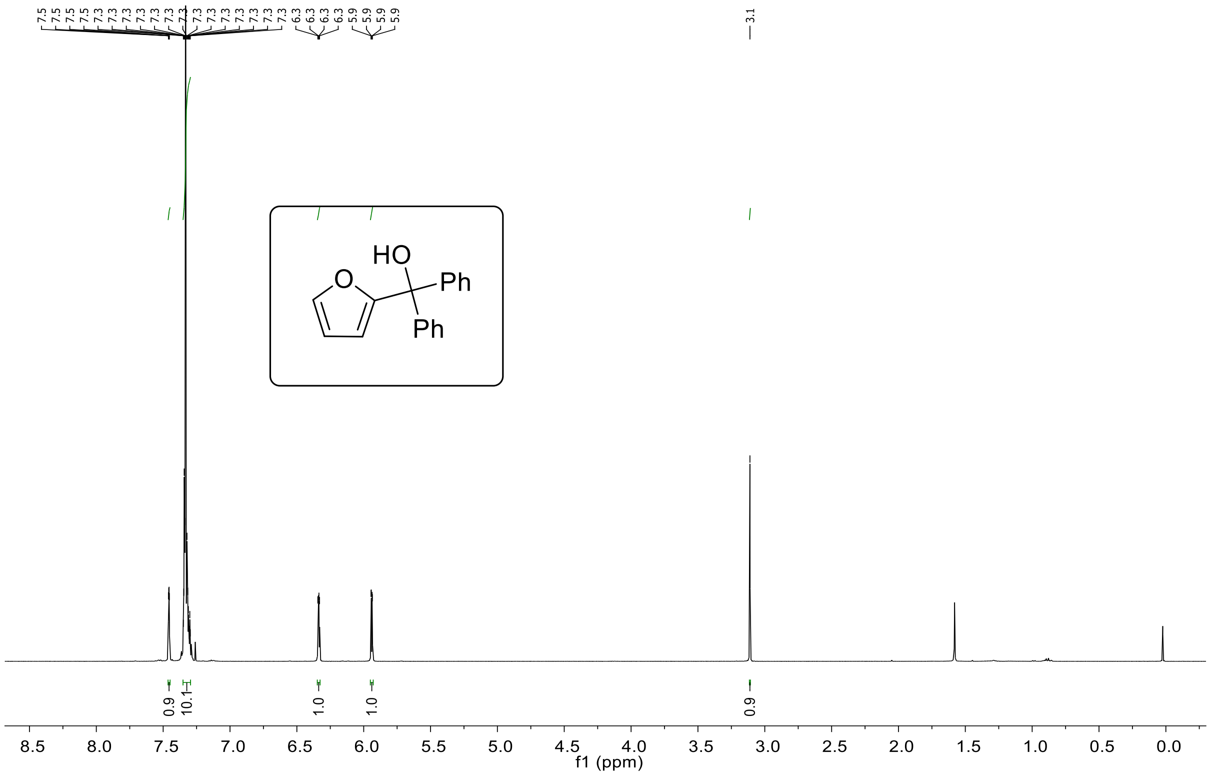

^{1}H NMR (400 MHz, $CDCl_3$) spectrum of furan-2-yldiphenylmethanol (**2f**)

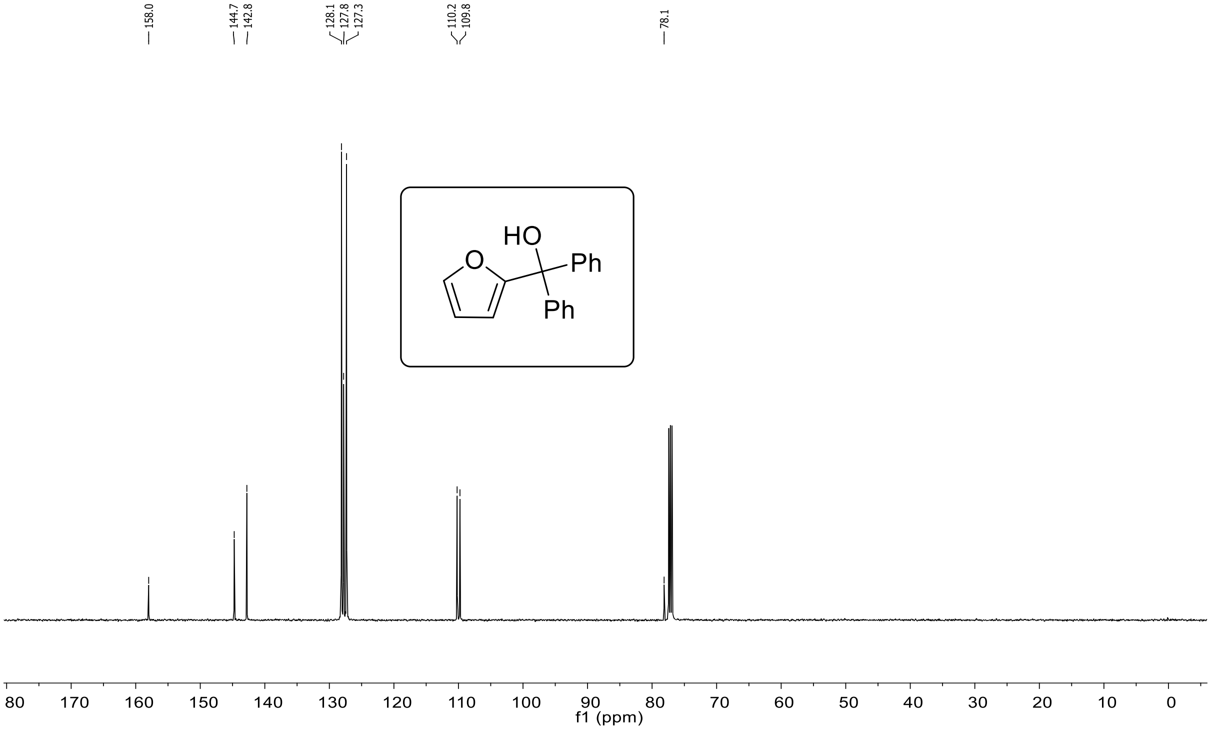

^{13}C NMR (125 MHz, $CDCl_3$) spectrum of furan-2-yldiphenylmethanol (**2f**)

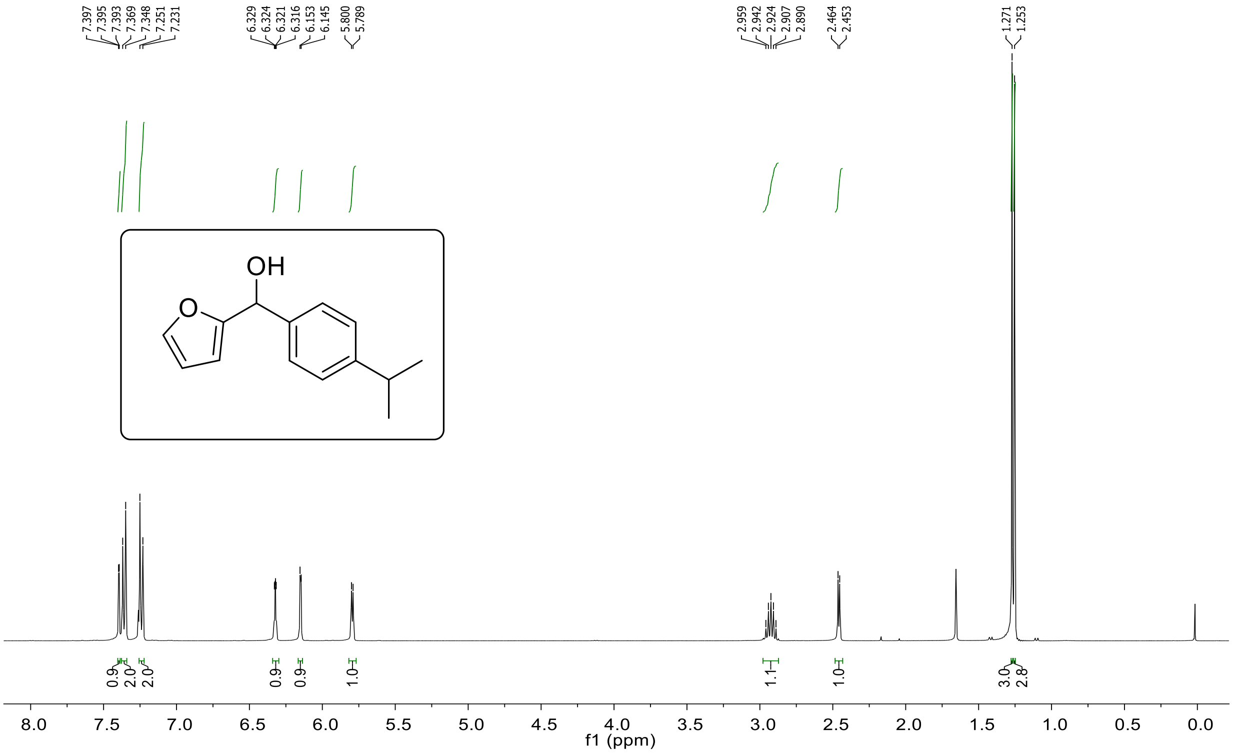

^{1}H NMR (400 MHz, $CDCl_3$) spectrum of furan-2-yl(4-isopropylphenyl)methanol (**2g**)

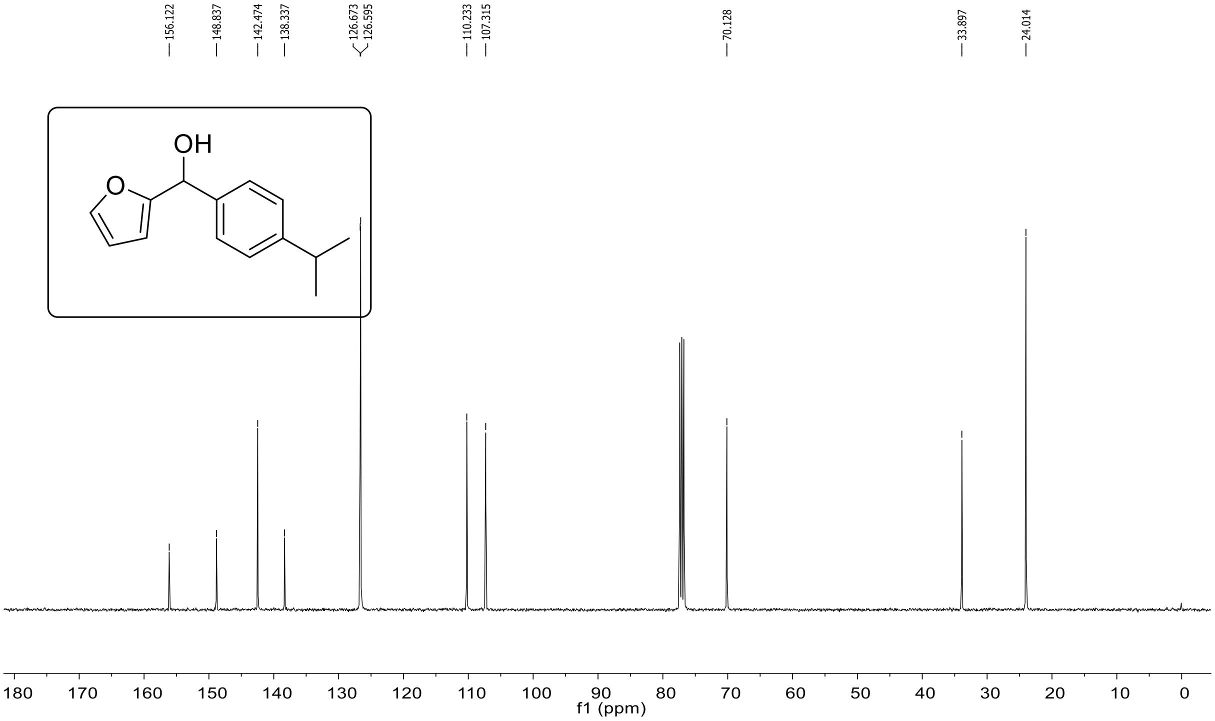

^{13}C NMR (100 MHz, $CDCl_3$) spectrum of furan-2-yl(4-isopropylphenyl)methanol (**2g**)

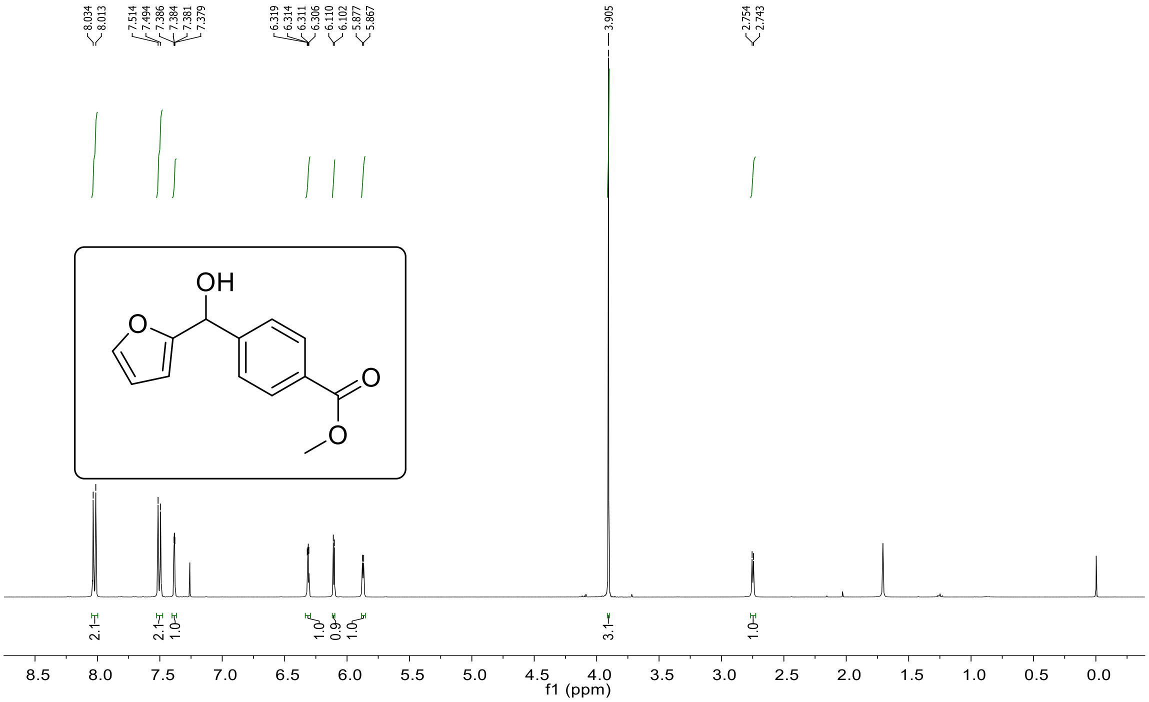

^{1}H NMR (400 MHz, $CDCl_3$) spectrum of methyl 4-(furan-2-yl(hydroxy)methyl)benzoate (**2h**)

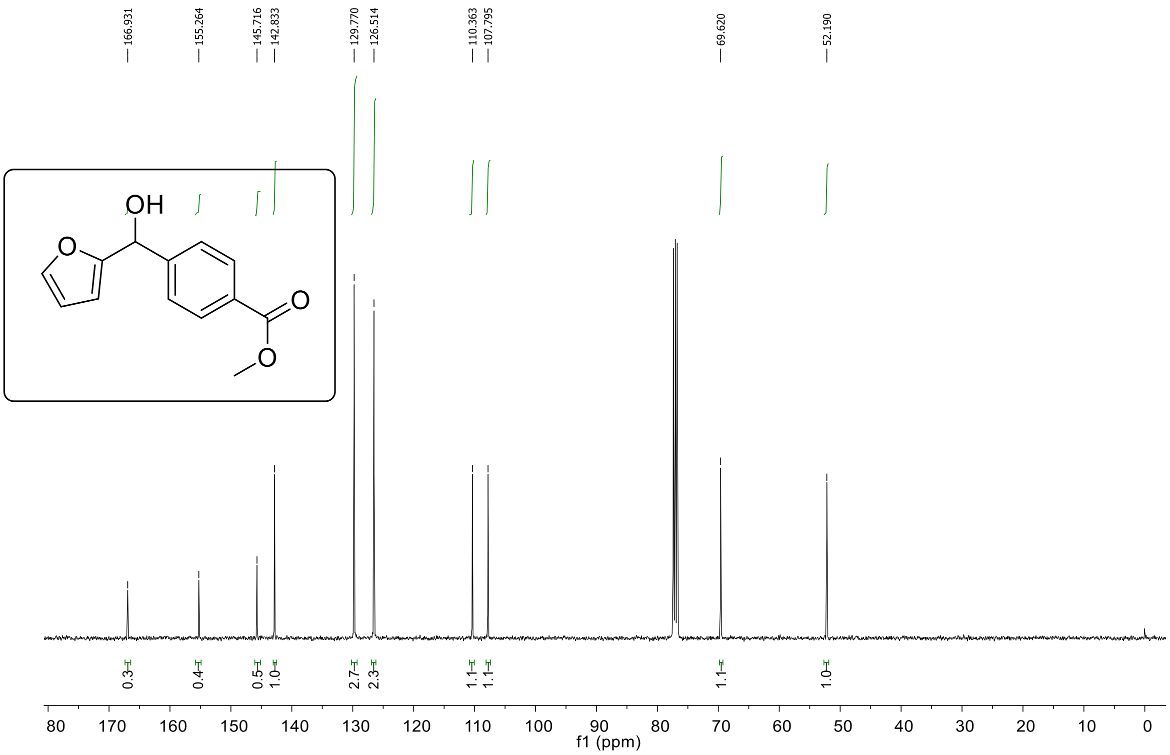

^{13}C NMR (100 MHz, $CDCl_3$) spectrum of methyl 4-(furan-2-yl(hydroxy)methyl)benzoate (**2h**)

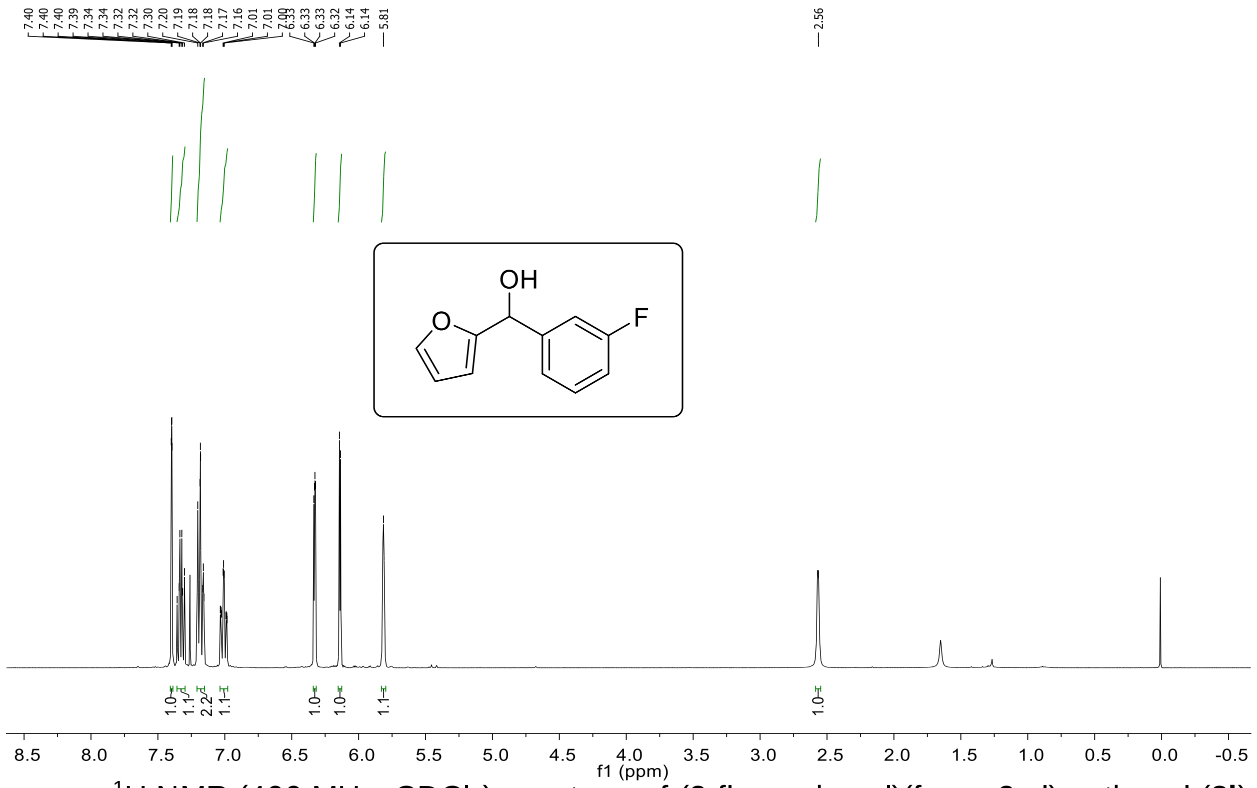

^1H NMR (400 MHz, $CDCl_3$) spectrum of (3-fluorophenyl)(furan-2-yl)methanol (**2i**)

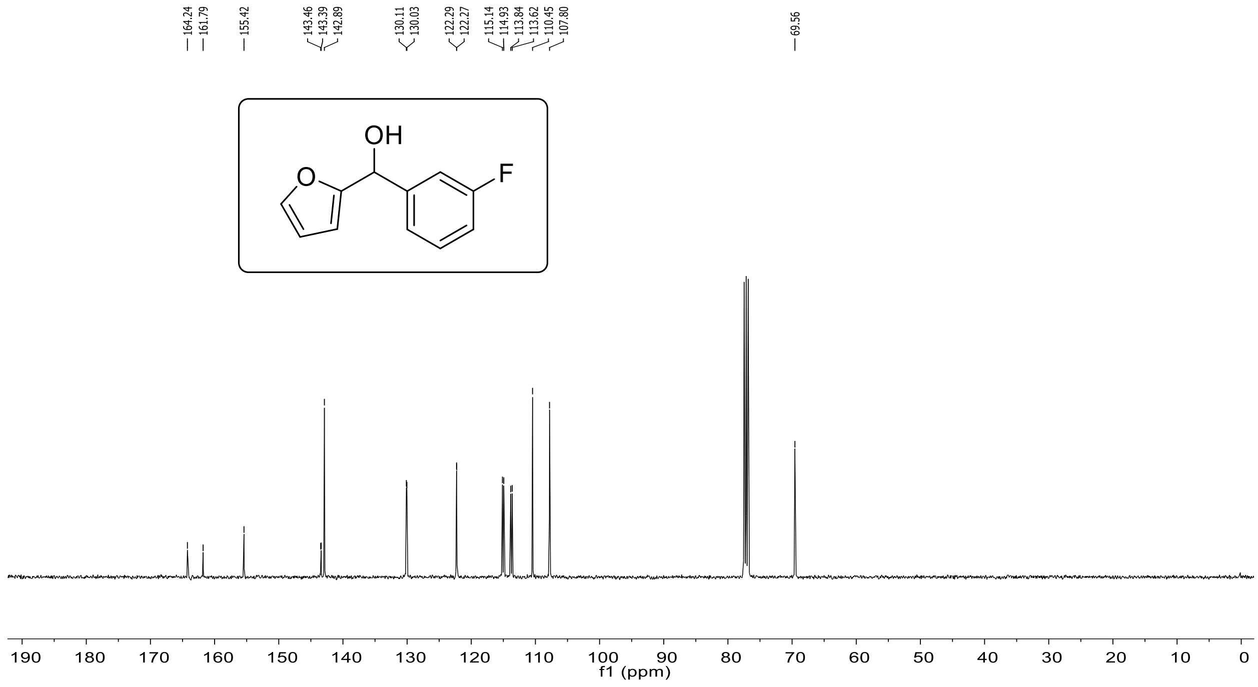

^{13}C NMR (100 MHz, $CDCl_3$) spectrum of (3-fluorophenyl)(furan-2-yl)methanol (**2i**)

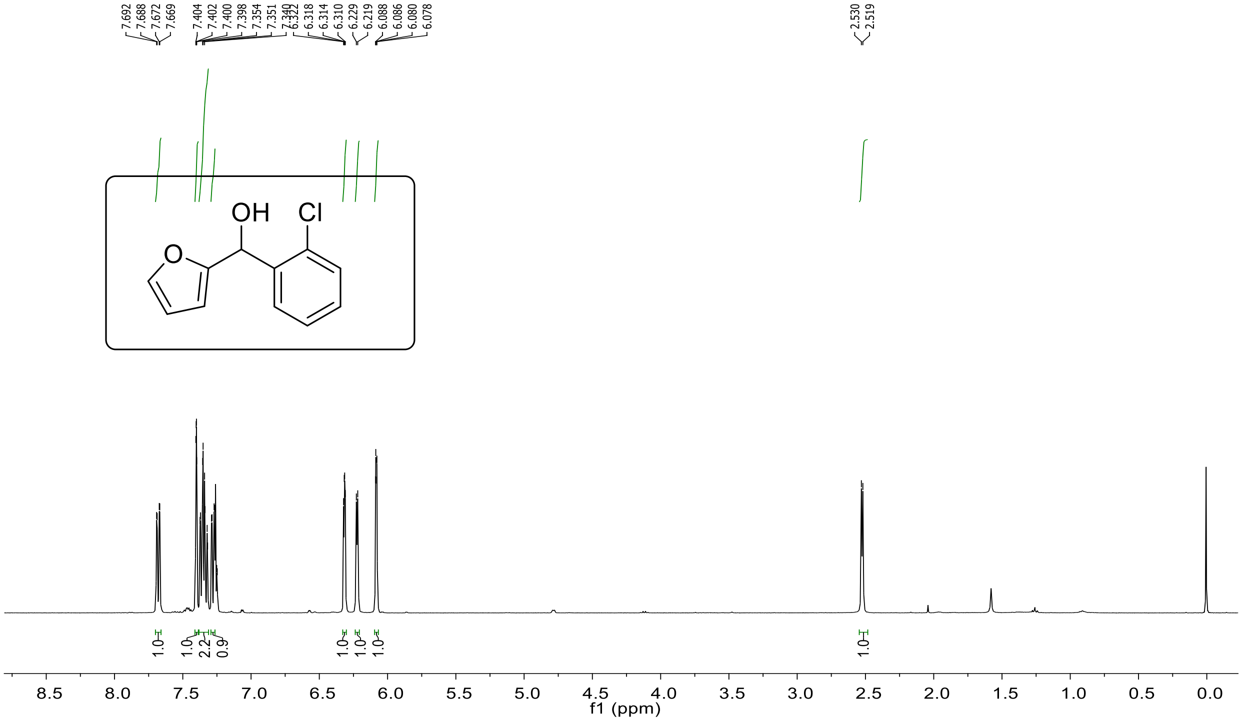

^1H NMR (400 MHz, $CDCl_3$) spectrum of (2-chlorophenyl)(furan-2-yl)methanol (**2j**)

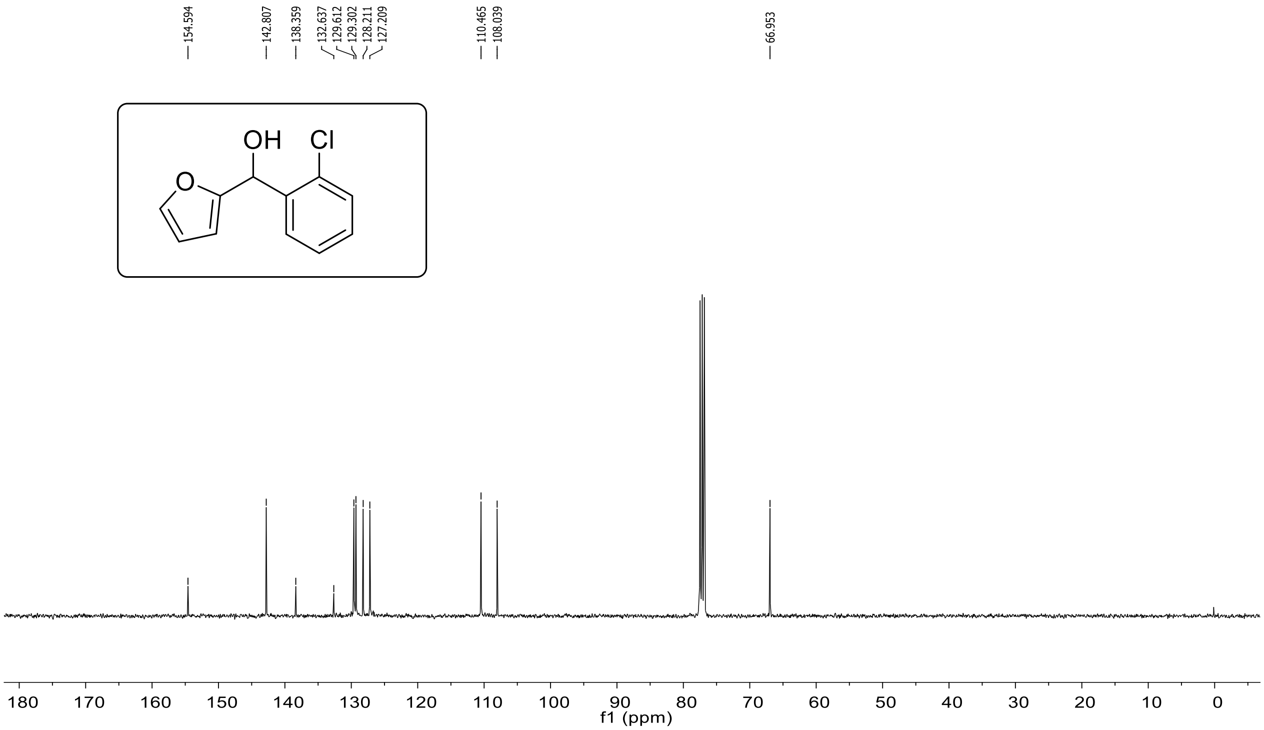

^{13}C NMR (100 MHz, $CDCl_3$) spectrum of (2-chlorophenyl)(furan-2-yl)methanol (**2j**)

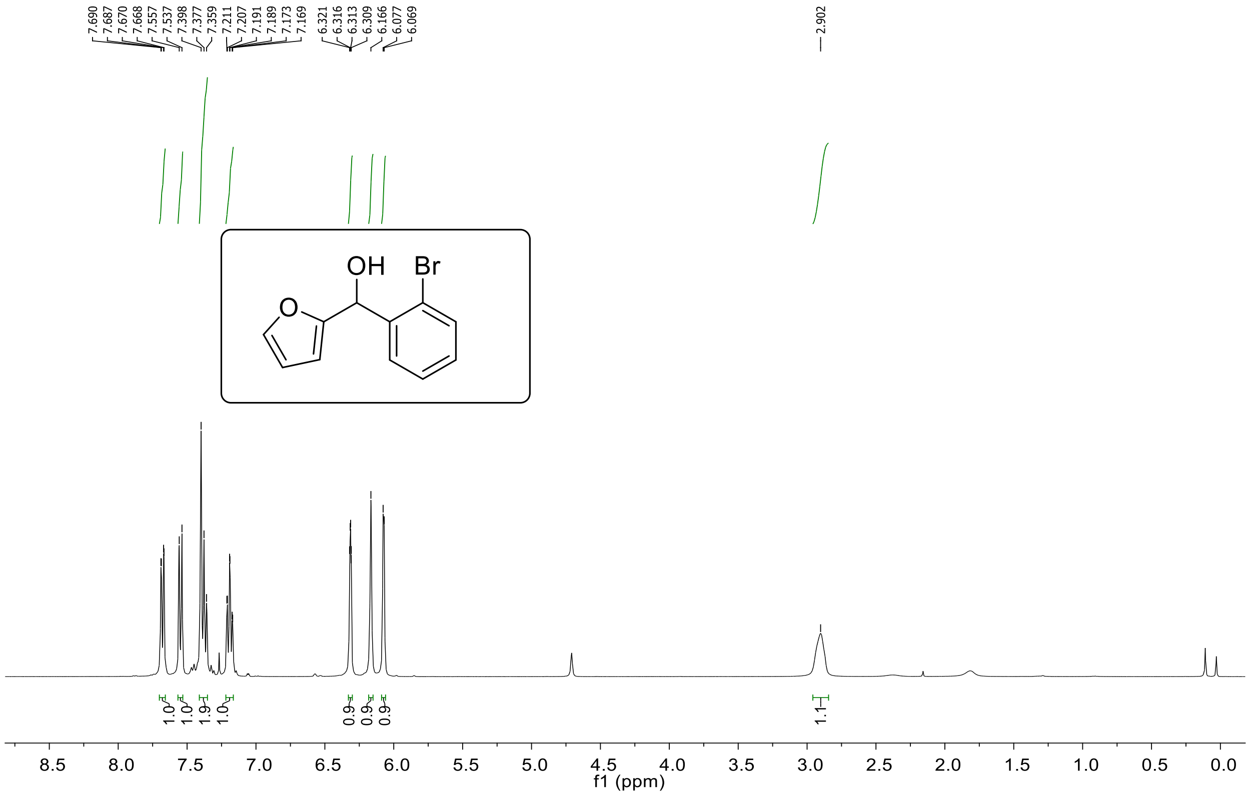

^{1}H NMR (400 MHz, $CDCl_3$) spectrum of (2-bromophenyl)(furan-2-yl)methanol (**2k**)

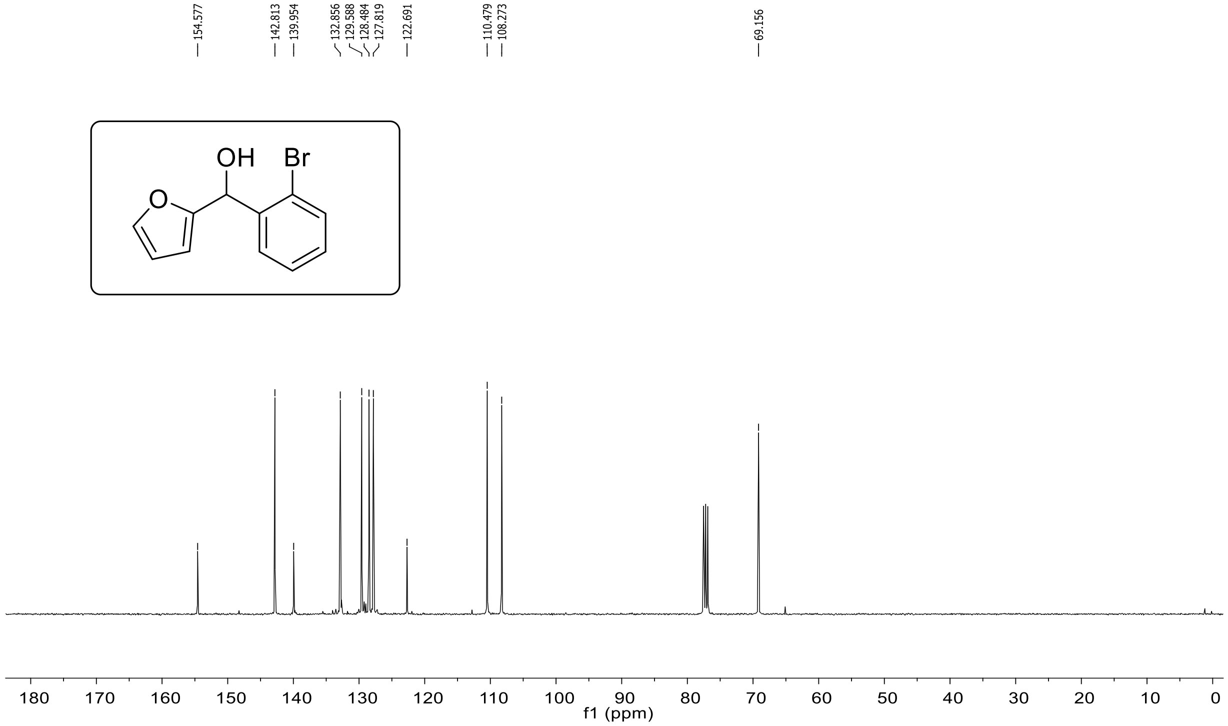

^{13}C NMR (100 MHz, $CDCl_3$) spectrum of (2-bromophenyl)(furan-2-yl)methanol (**2k**)

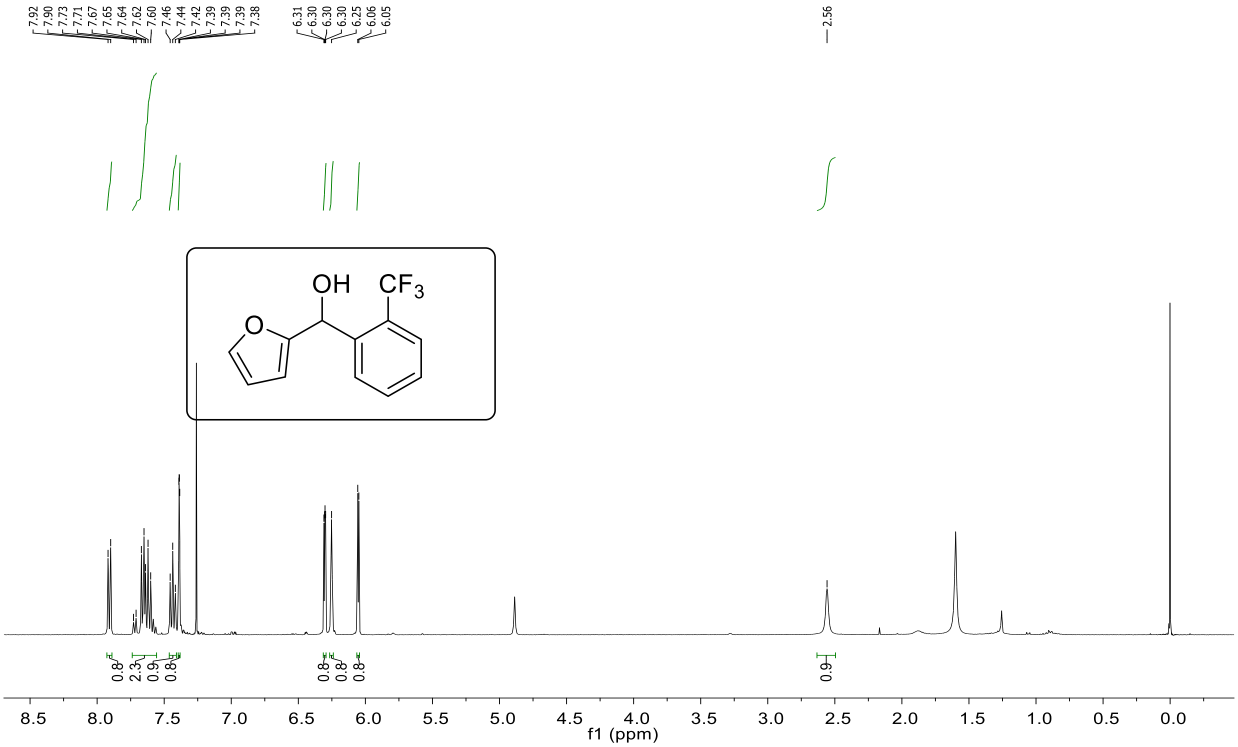

^{1}H NMR (400 MHz, $CDCl_3$) spectrum of furan-2-yl(2-(trifluoromethyl)phenyl)methanol (**2l**)

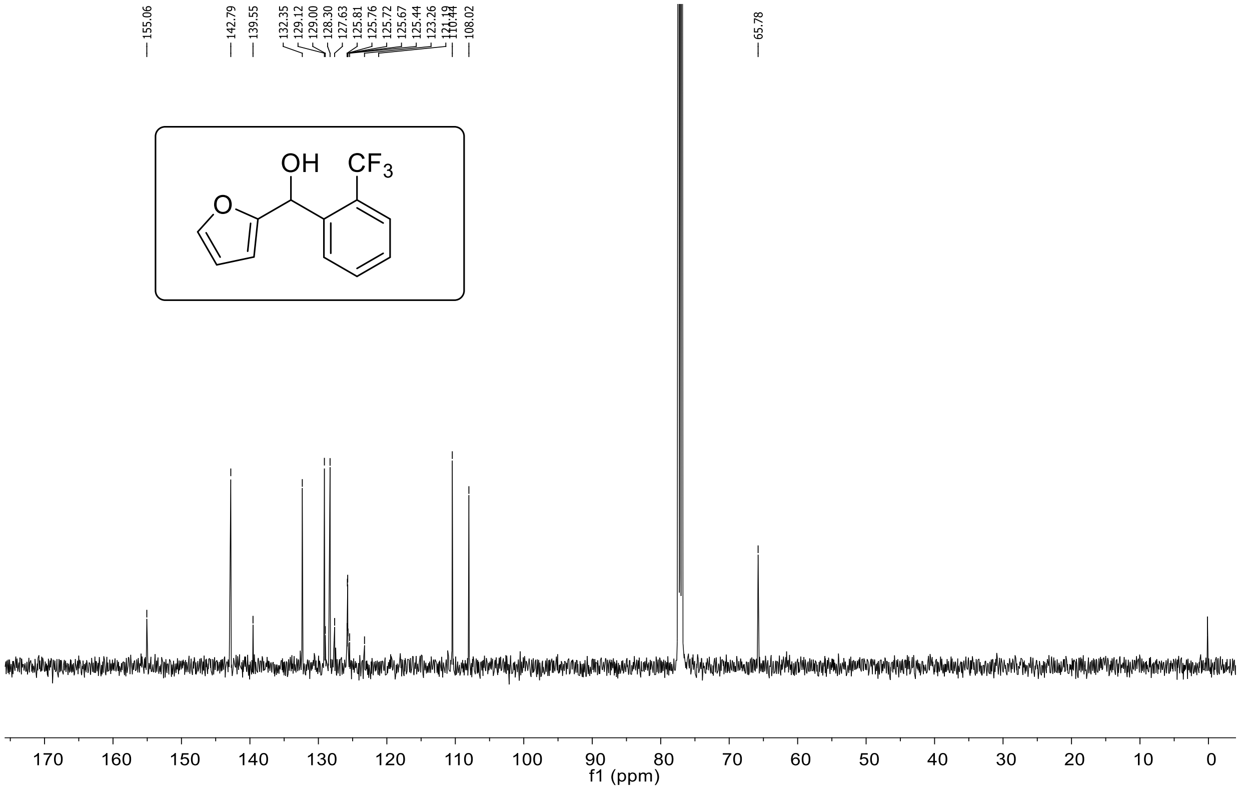

^{13}C NMR (125 MHz, $CDCl_3$) spectrum of furan-2-yl(2-(trifluoromethyl)phenyl)methanol (**2l**)

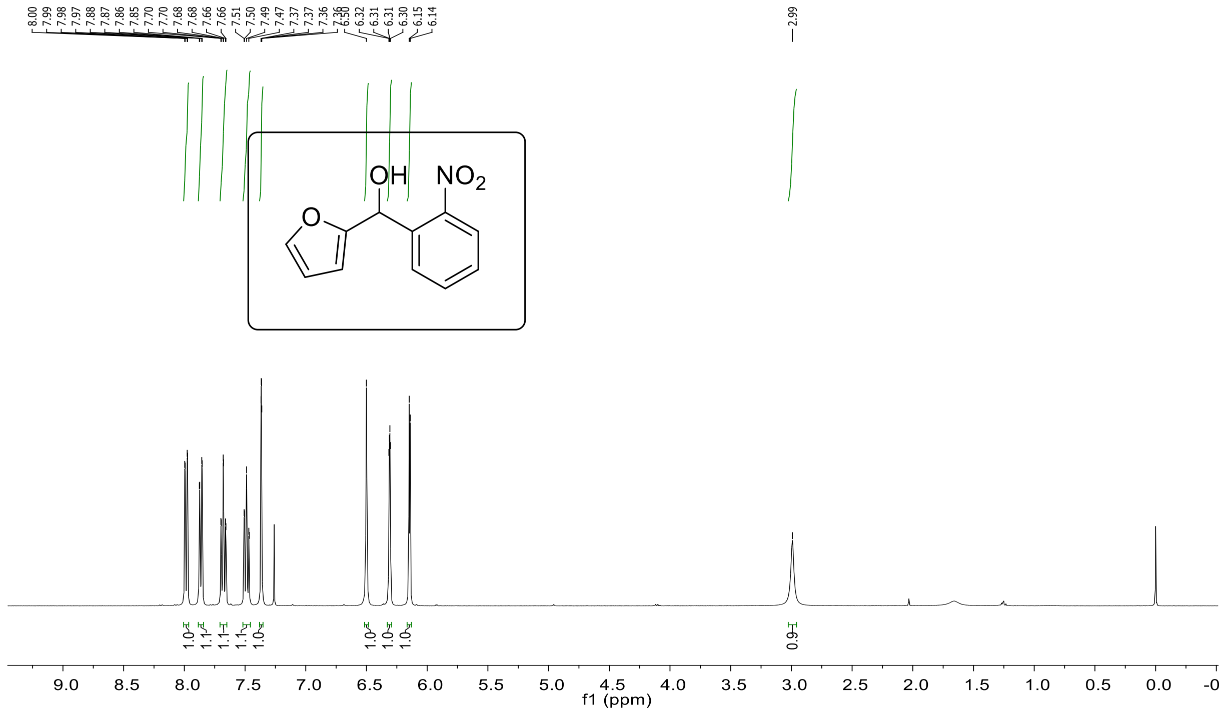

^{1}H NMR (400 MHz, $CDCl_3$) spectrum of furan-2-yl(2-nitrophenyl)methanol (**2m**)

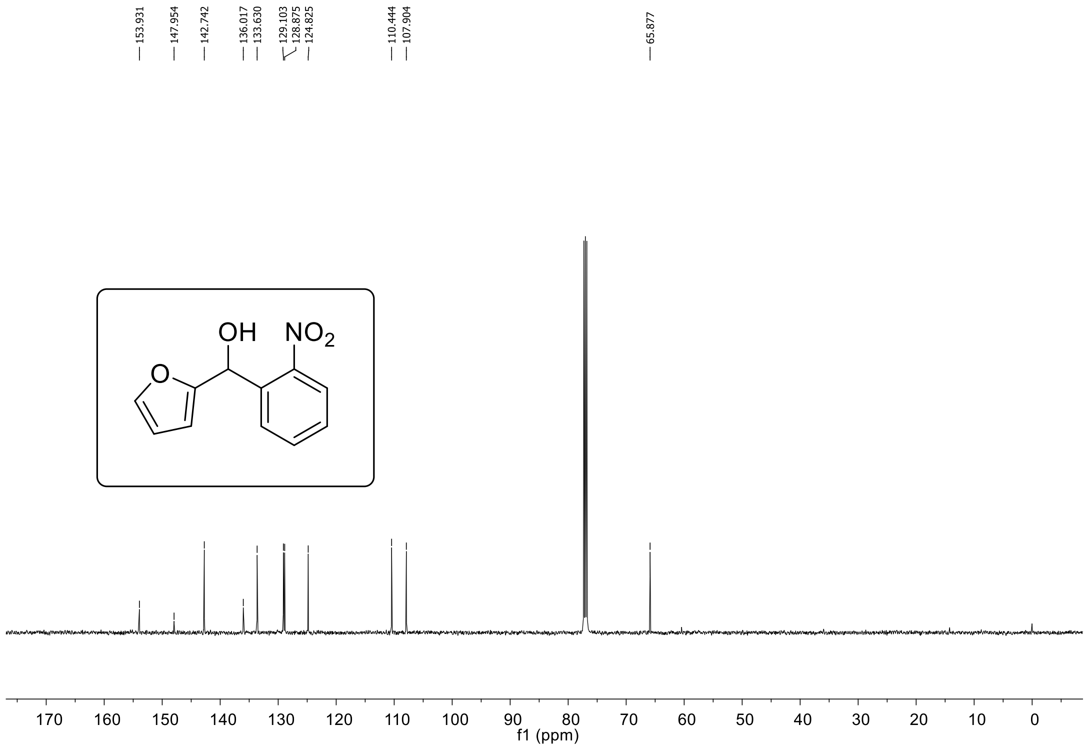

^{13}C NMR (126 MHz, $CDCl_3$) spectrum of furan-2-yl(2-nitrophenyl)methanol (**2m**)

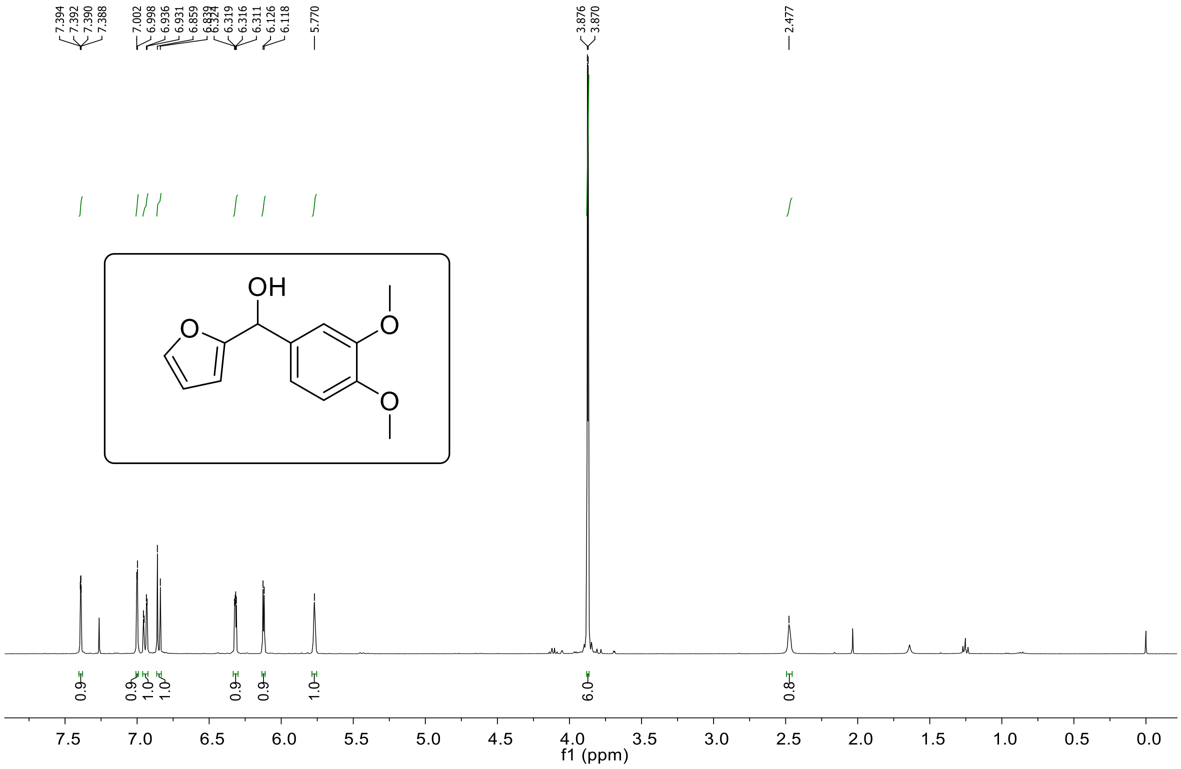

^{1}H NMR (400 MHz, $CDCl_3$) spectrum of (3,4-dimethoxyphenyl)(furan-2-yl)methanol **(2n)**

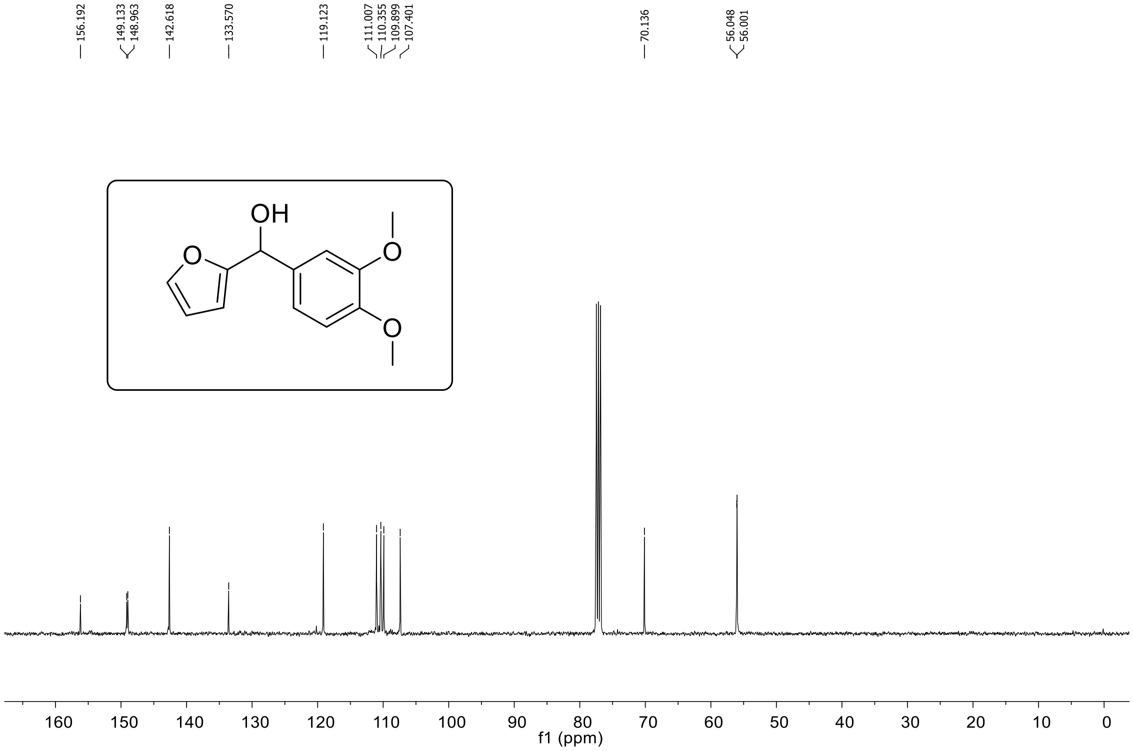

^{13}C NMR (100 MHz, $CDCl_3$) spectrum of (3,4-dimethoxyphenyl)(furan-2-yl)methanol(**2n**)

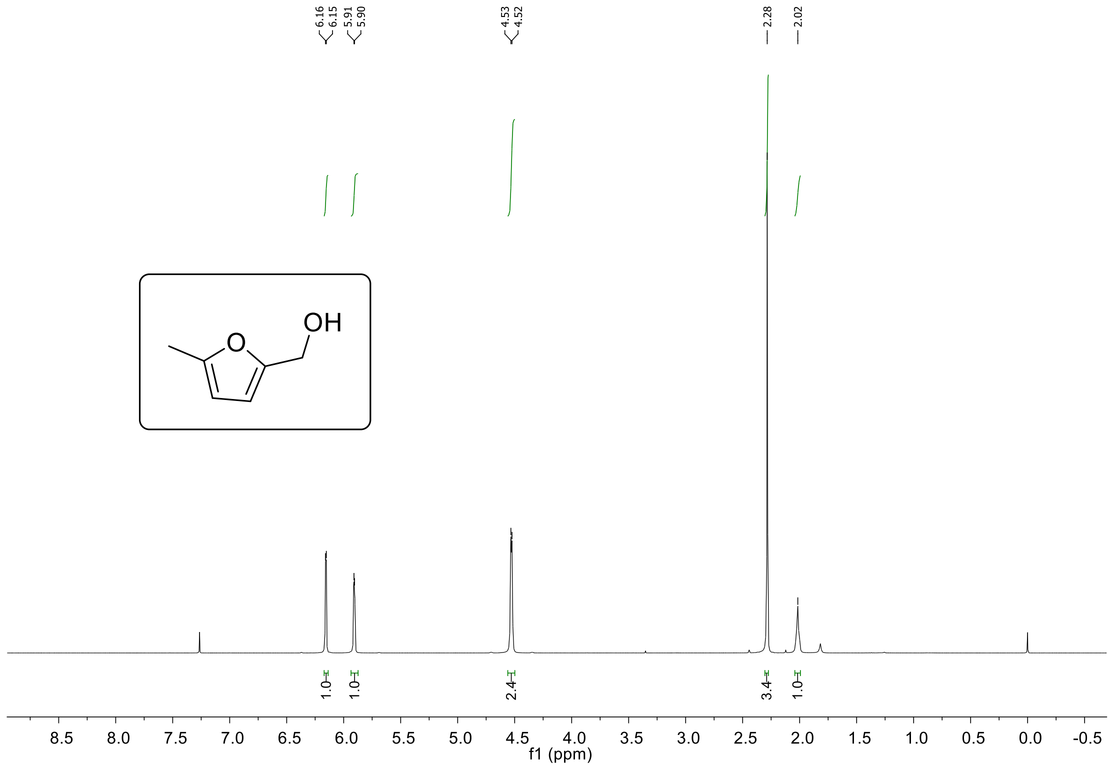

^{1}H NMR (400 MHz, $CDCl_3$) spectrum of (5-methylfuran-2-yl)methanol (**2o**)

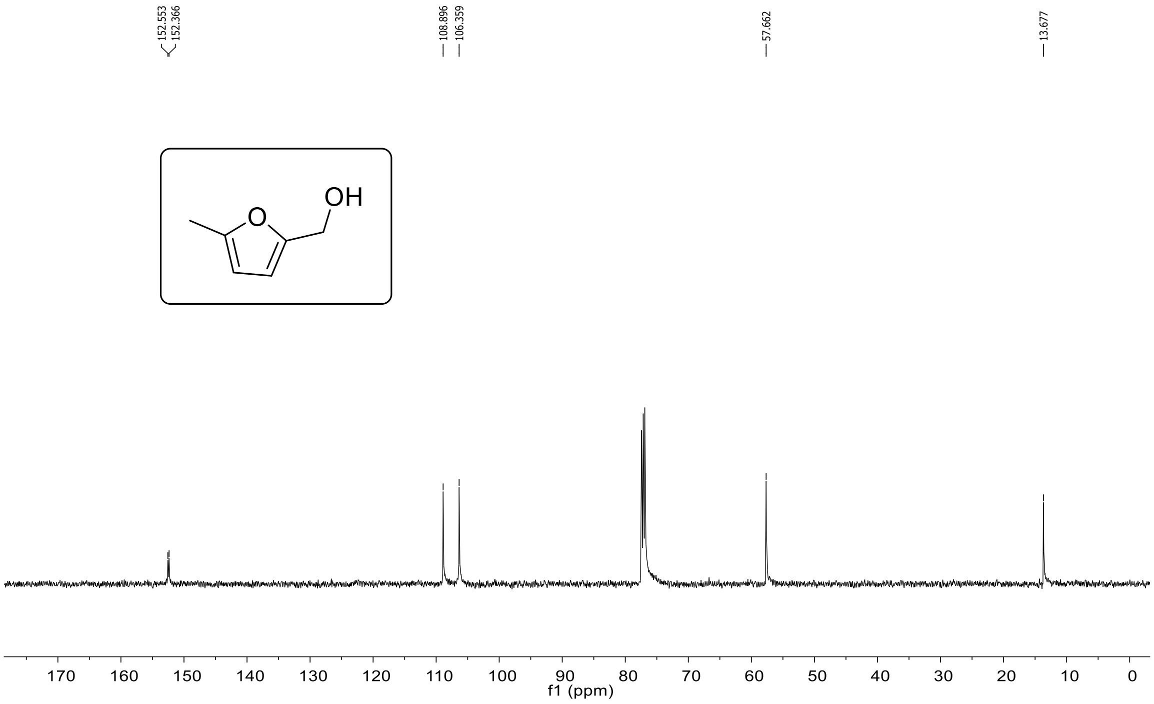

^{13}C NMR (125 MHz, $CDCl_3$) spectrum of (5-methylfuran-2-yl)methanol (**2o**)

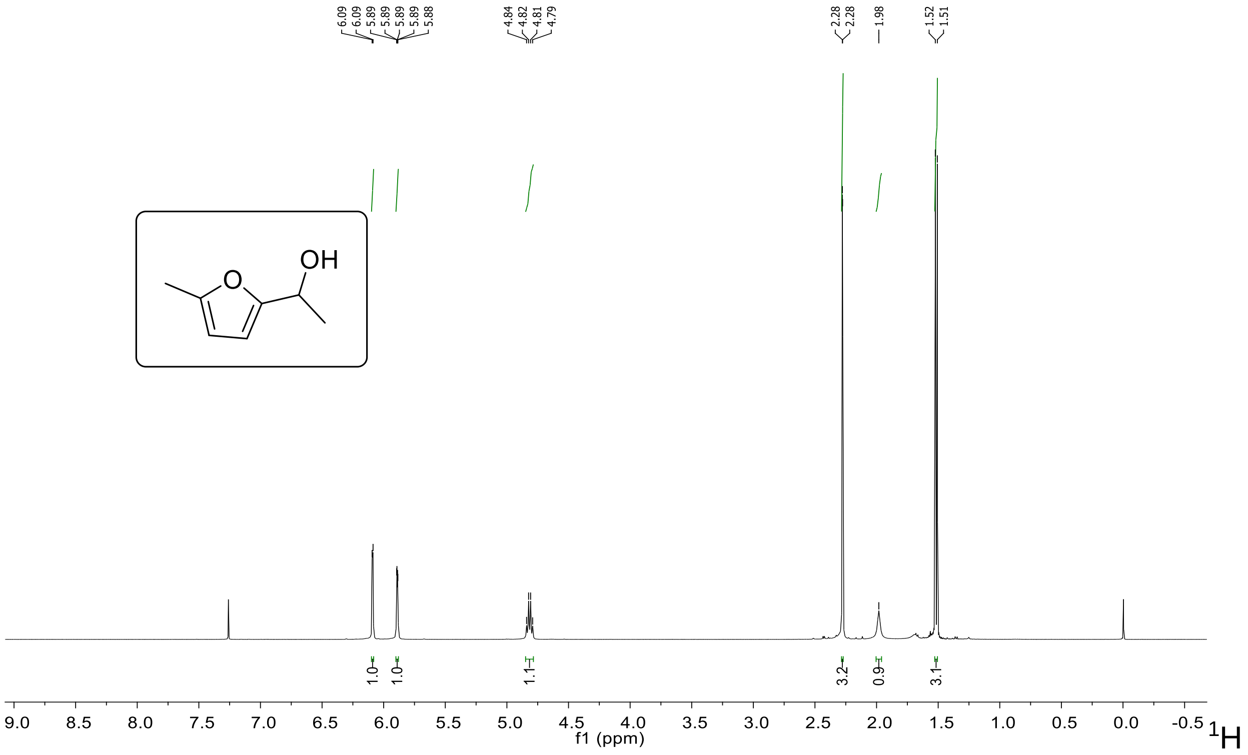

NMR (400 MHz, CDCl3) spectrum of 1-(5-methylfuran-2-yl)ethan-1-ol **(2p)**

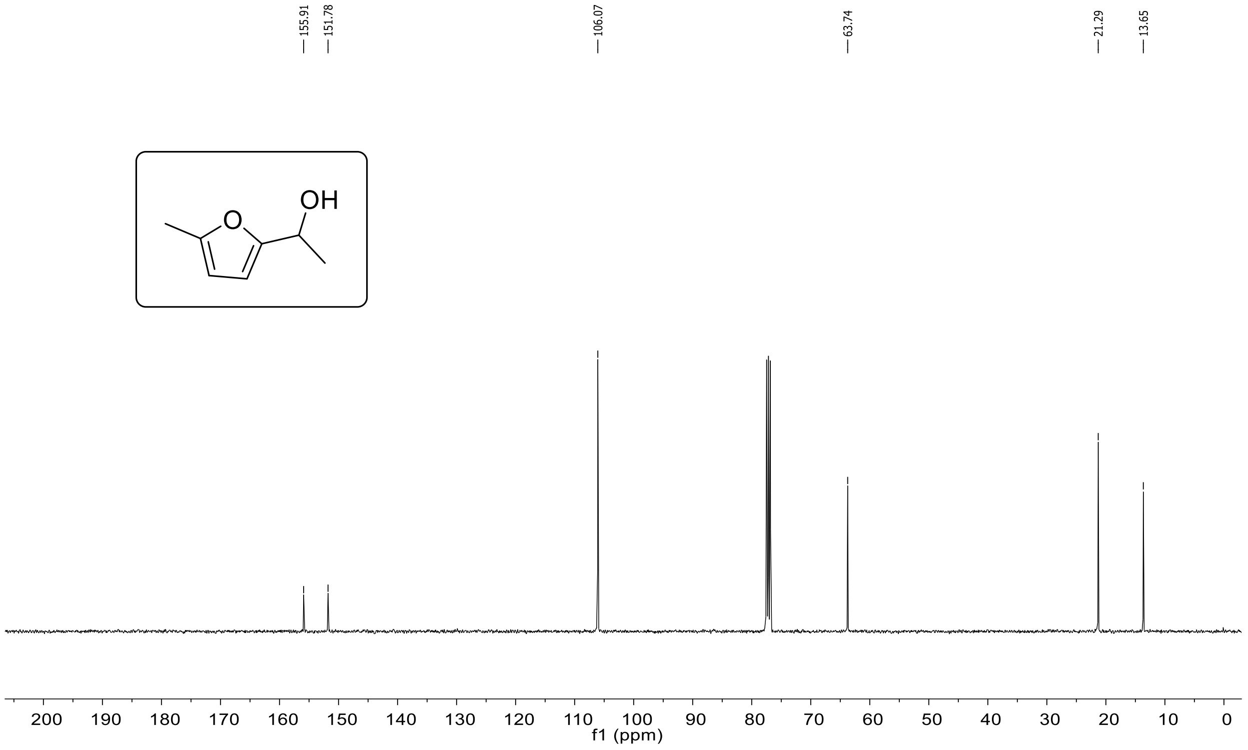

^{13}C NMR (101 MHz, CDCl3) spectrum of 1-(5-methylfuran-2-yl)ethan-1-ol (**2p**)

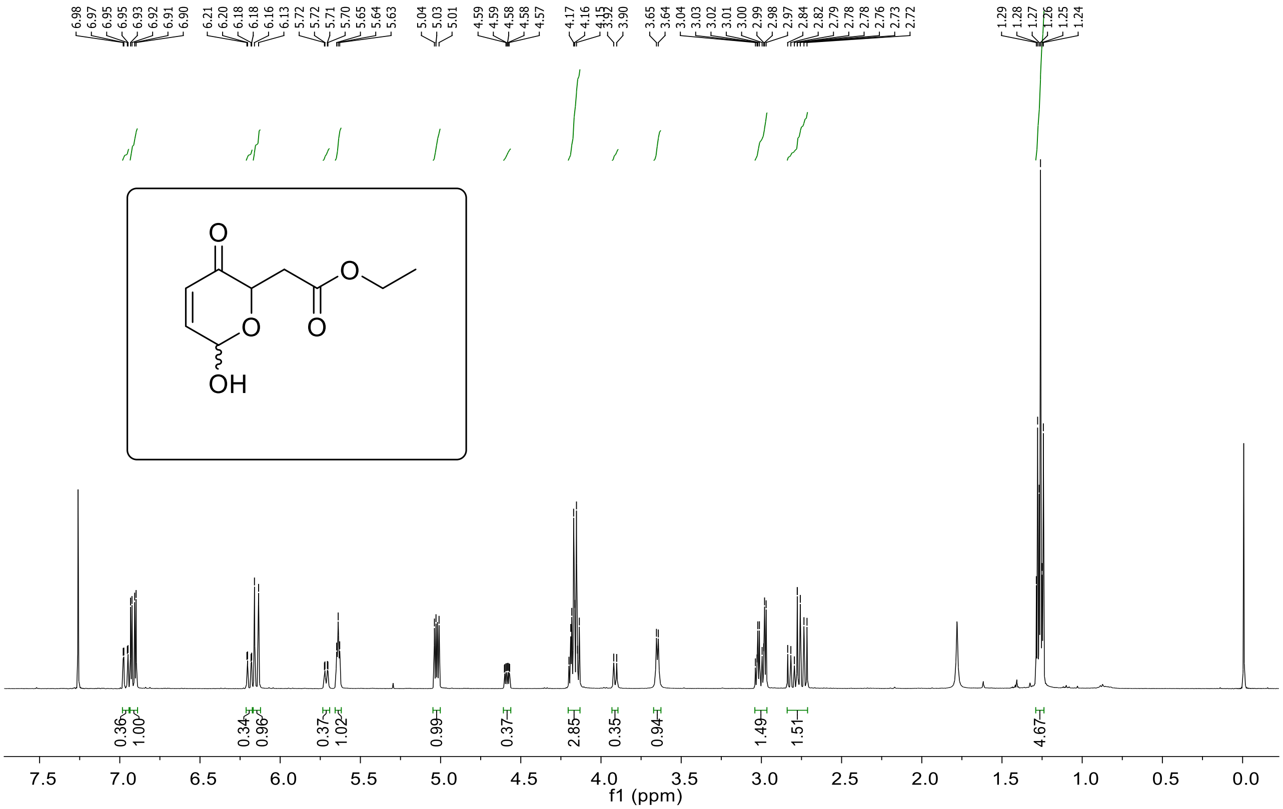

^{1}H NMR (400 MHz, $CDCl_3$) spectrum of ethyl 2-(6-hydroxy-3-oxo-3,6-dihydro-2*H*-pyran-2-yl)acetate (**3a**)

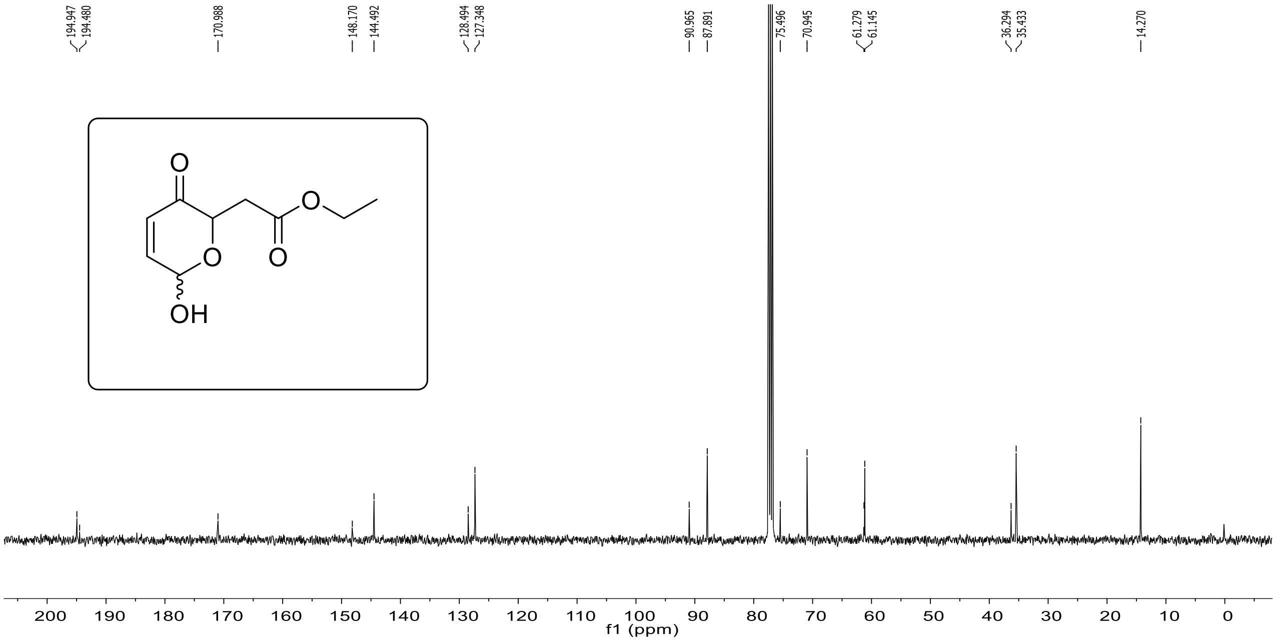

^{13}C NMR (100 MHz, $CDCl_3$) spectrum of ethyl 2-(6-hydroxy-3-oxo-3,6-dihydro-2*H*-pyran-2-yl)acetate (**3a**)

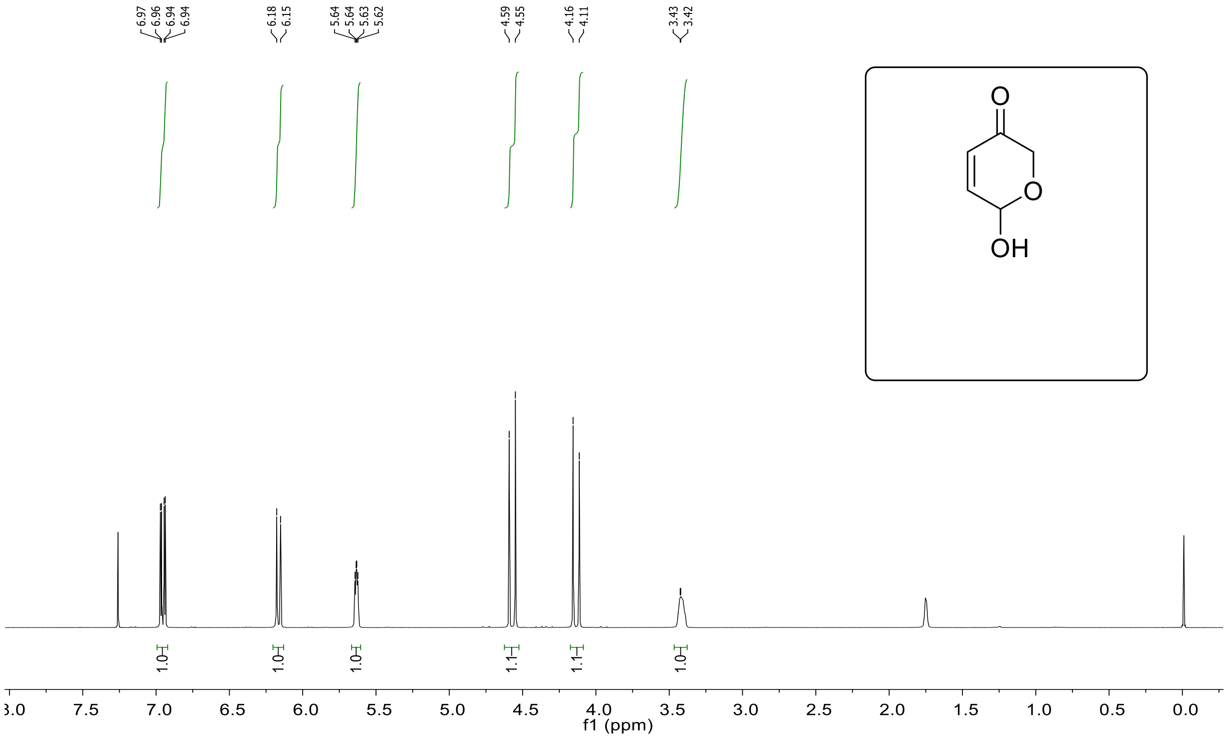

^{1}H NMR (400 MHz, $CDCl_3$) 6-hydroxy-2*H*-pyran-3(6*H*)-one (**3b**)

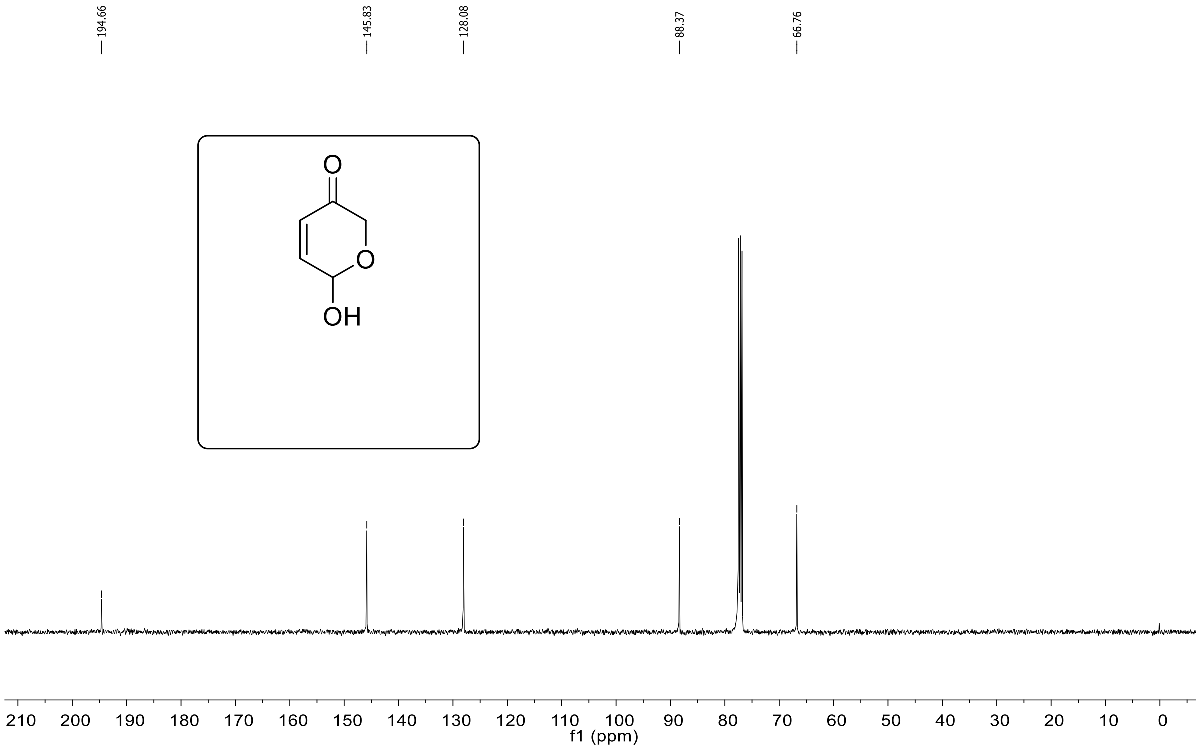

^{13}C NMR(100 MHz, CDCl3) spectrum of 6-hydroxy-2*H*-pyran-3(6*H*)-one (**3b**)

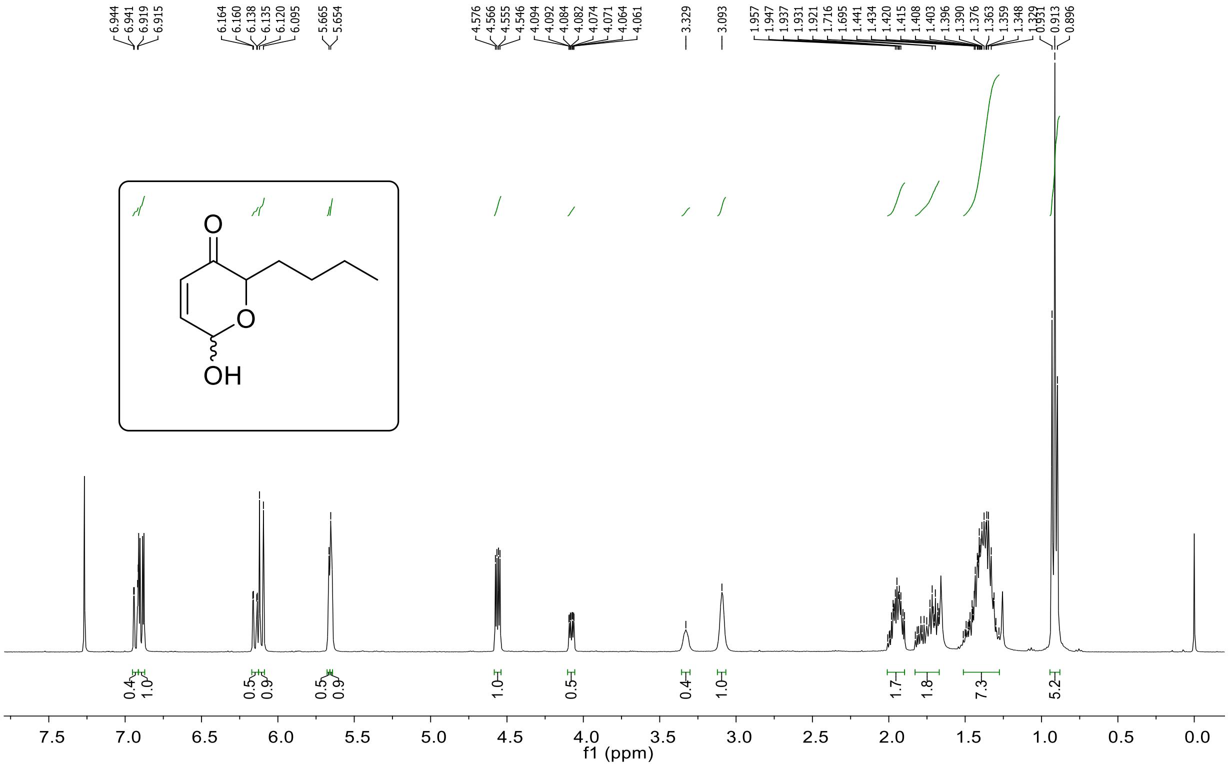

^{1}H NMR (400 MHz, $CDCl_3$) spectrum of 2-butyl-6-hydroxy-2*H*-pyran-3(6*H*)-one (**3c**)

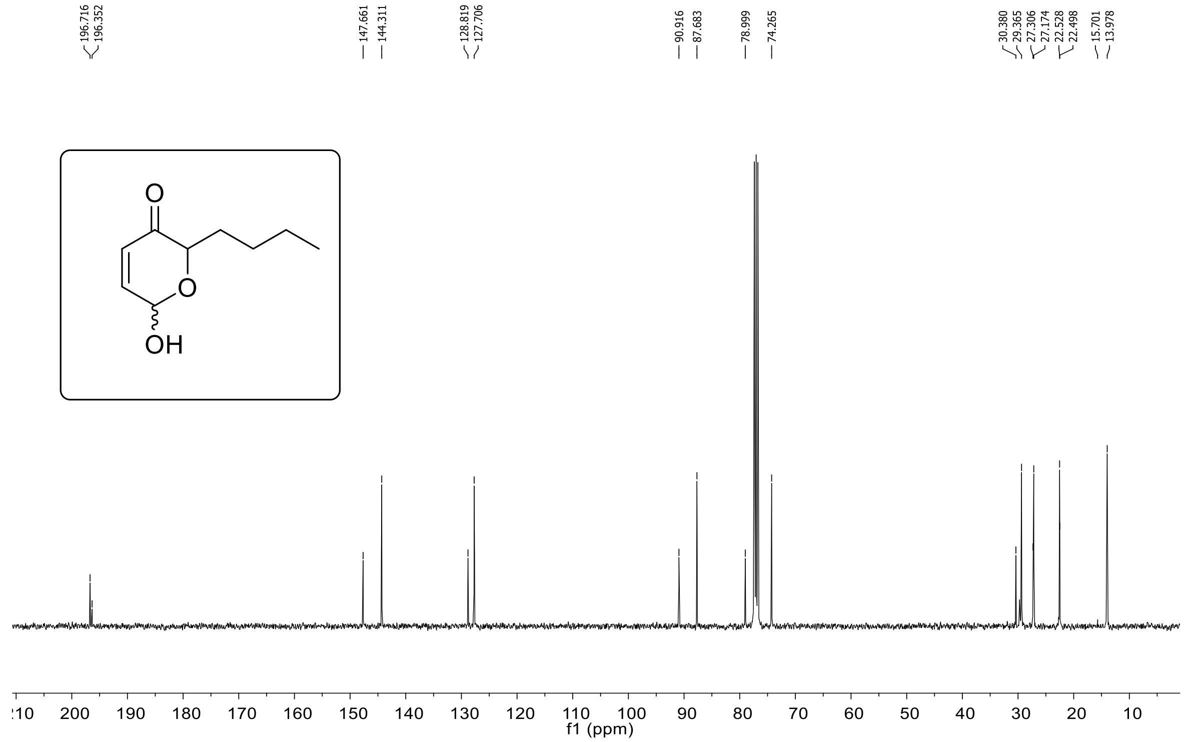

^{13}C NMR (100 MHz, $CDCl_3$) spectrum of 2-butyl-6-hydroxy-2*H*-pyran-3(6*H*)-one (**3c**)

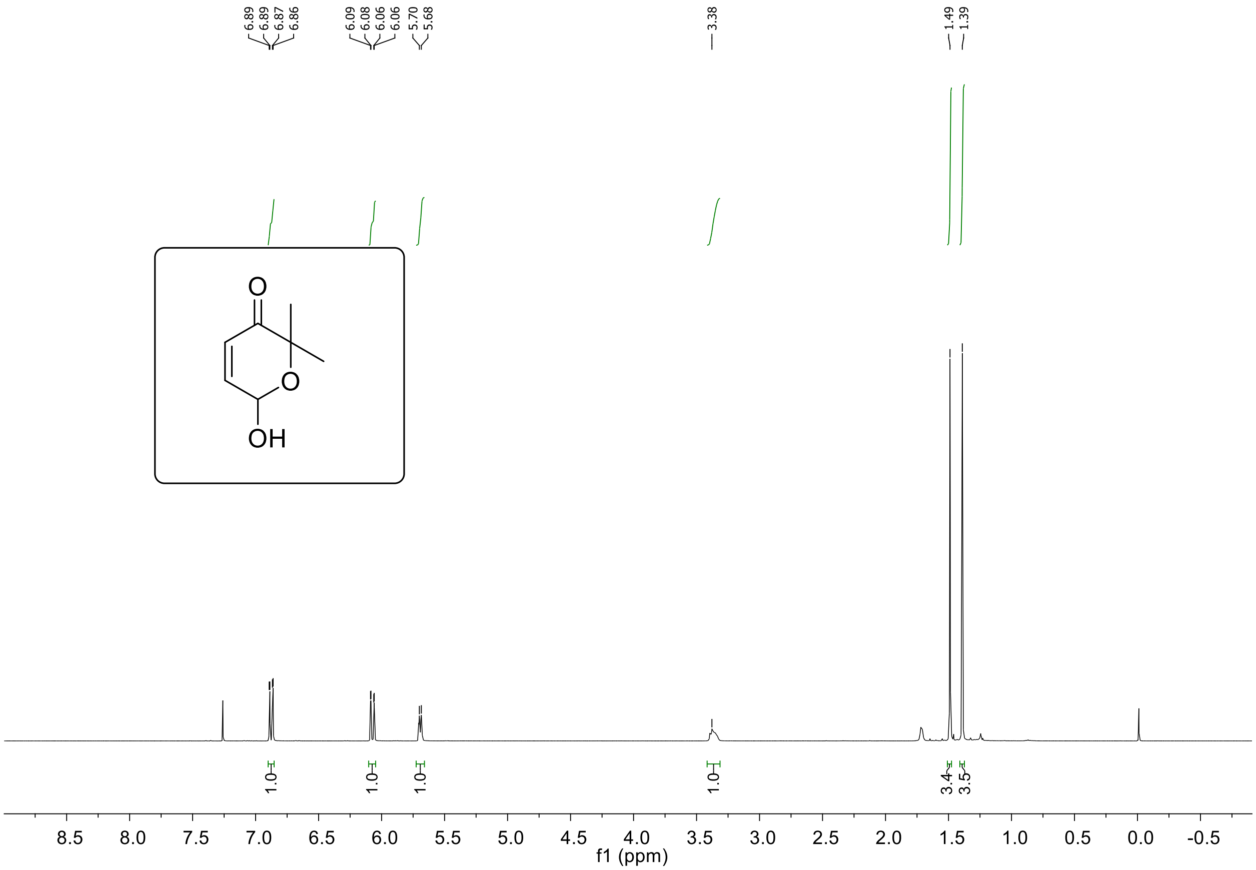

^{1}H NMR (400 MHz, $CDCl_3$) spectrum of 6-hydroxy-2,2-dimethyl-2*H*-pyran-3(6*H*)-one (**3d**)

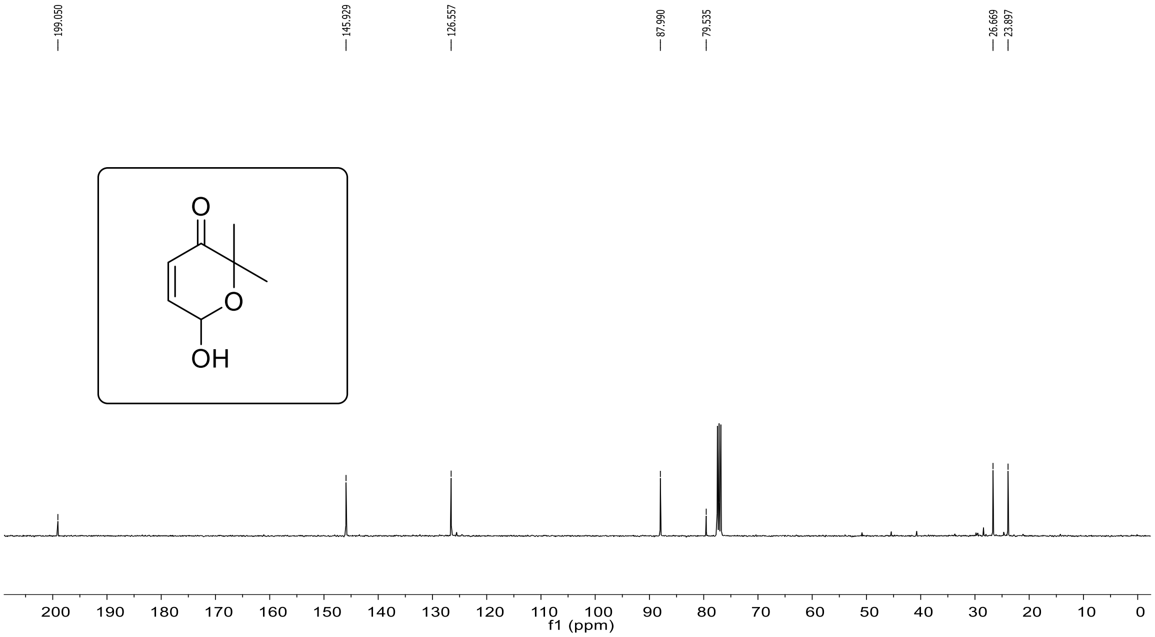

^{13}C NMR (100 MHz, $CDCl_3$) spectrum of 6-hydroxy-2,2-dimethyl-2*H*-pyran-3(6*H*)-one (**3d**)

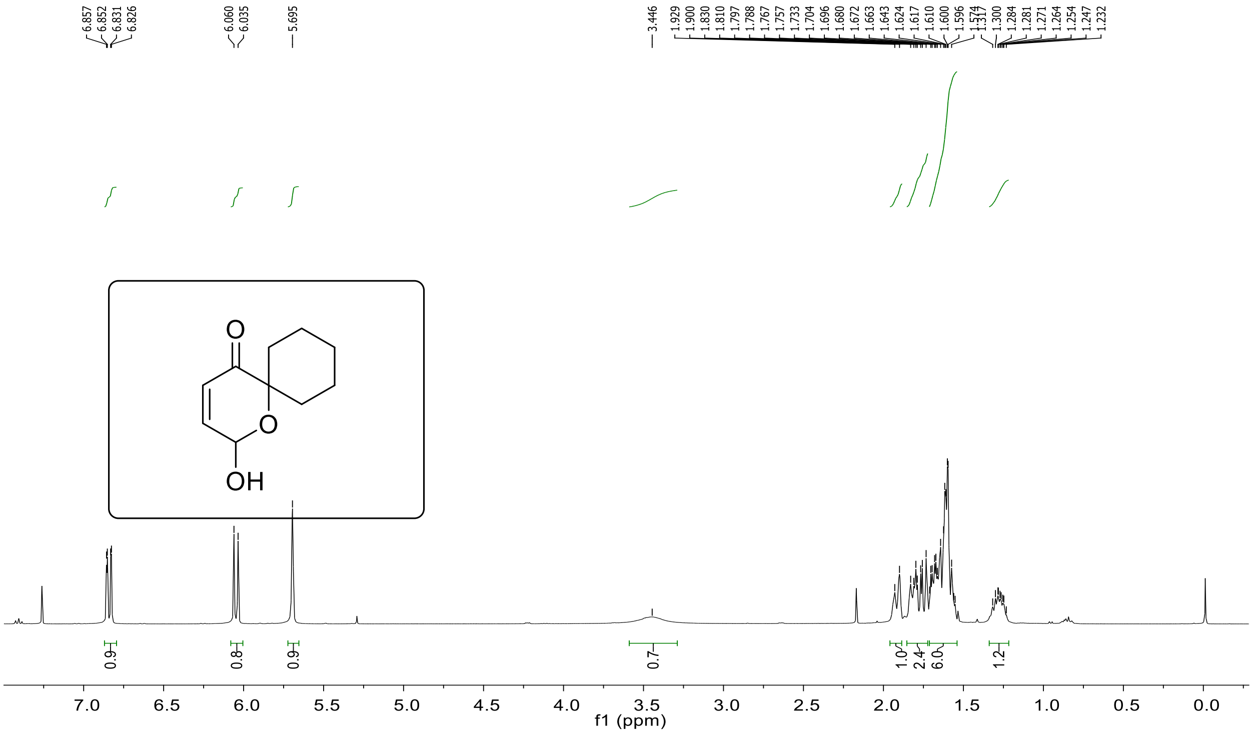

^{1}H NMR (400 MHz, $CDCl_3$) spectrum of 2-hydroxy-1-oxaspiro[5.5]undec-3-en-5-one (**3e**)

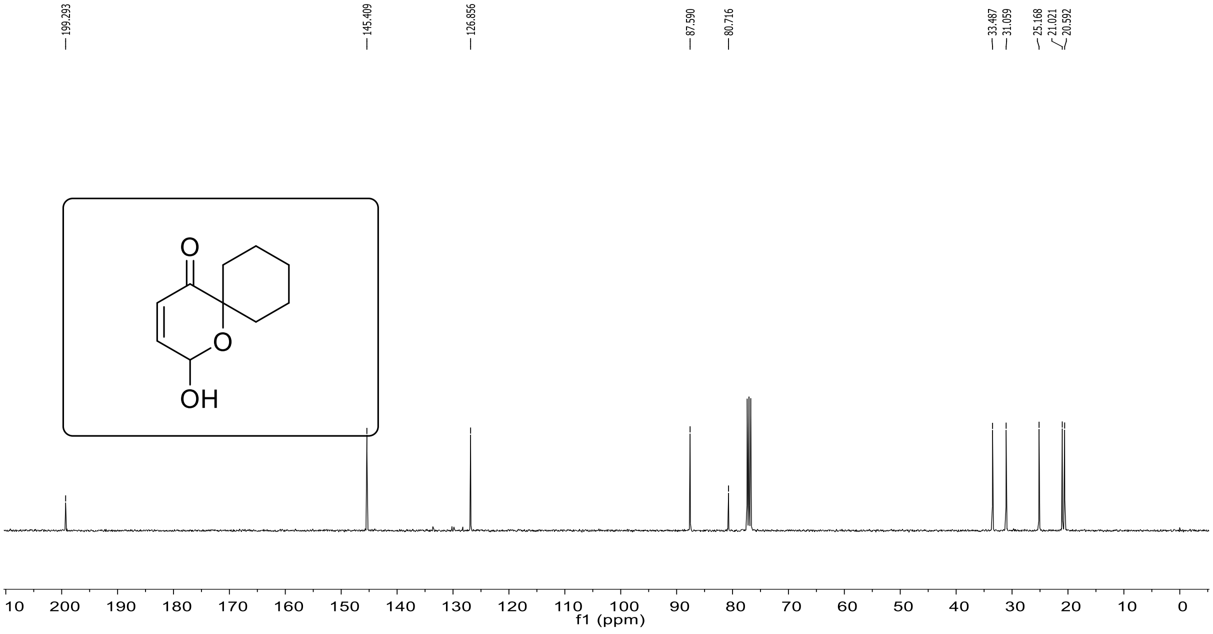

^{13}C NMR (100 MHz, $CDCl_3$) spectrum of 2-hydroxy-1-oxaspiro[5.5]undec-3-en-5-one (**3e**)

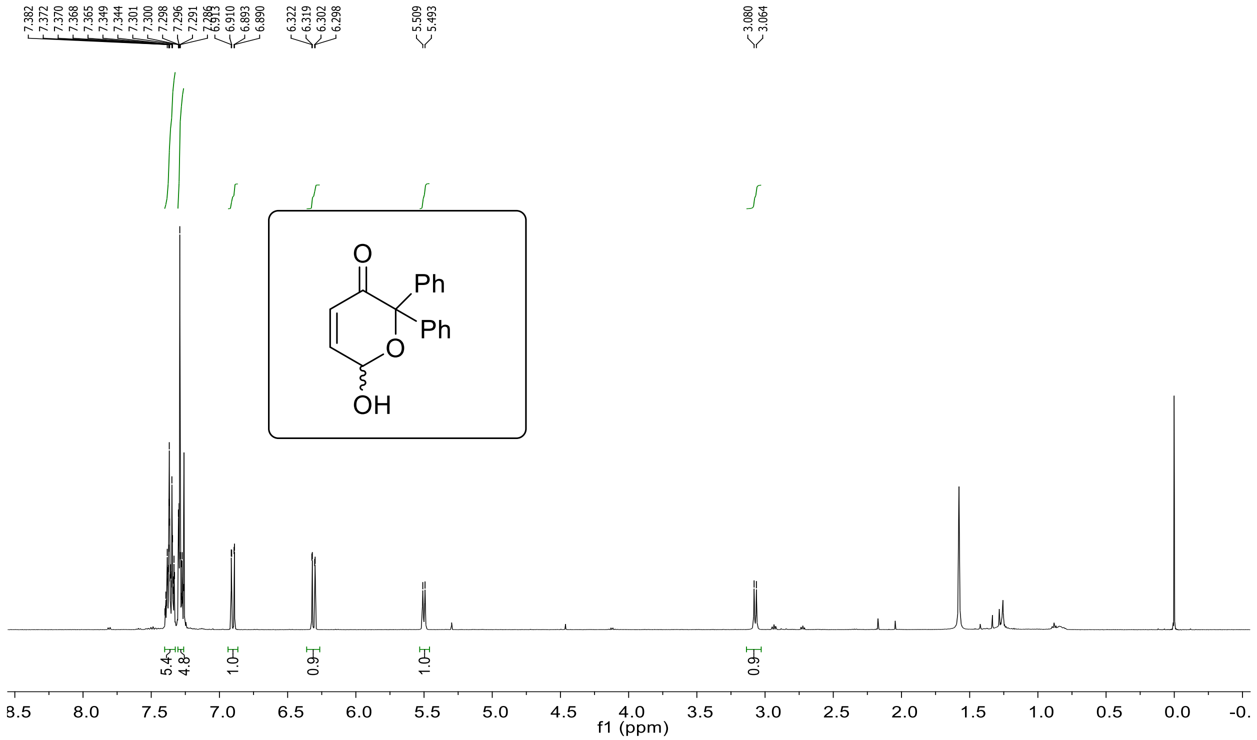

^{1}H NMR (500 MHz, CDCl$_3$)spectrum of 6-hydroxy-2,2-diphenyl-2*H*-pyran-3(6*H*)-one (**3f**)

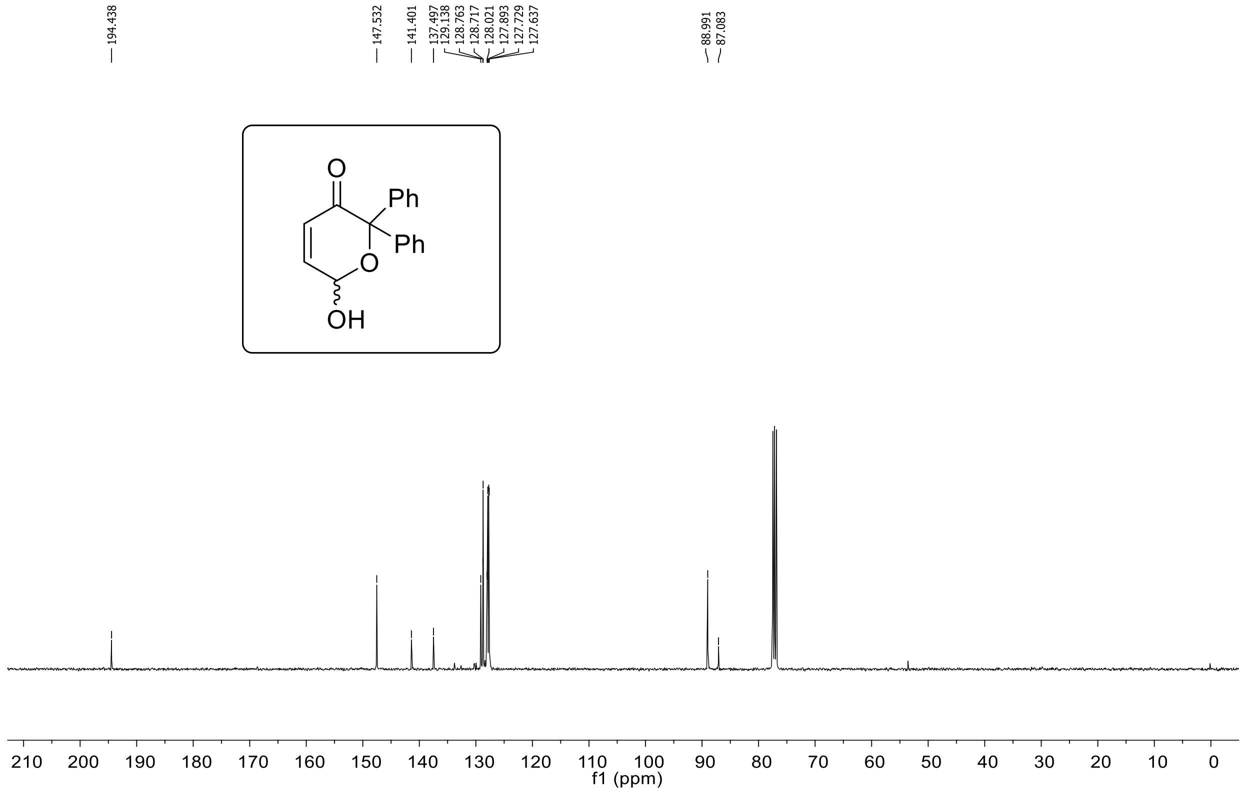

^{13}C NMR (100 MHz, CDCl$_3$) spectrum of 6-hydroxy-2,2-diphenyl-2*H*-pyran-3(6*H*)-one (**3f**)

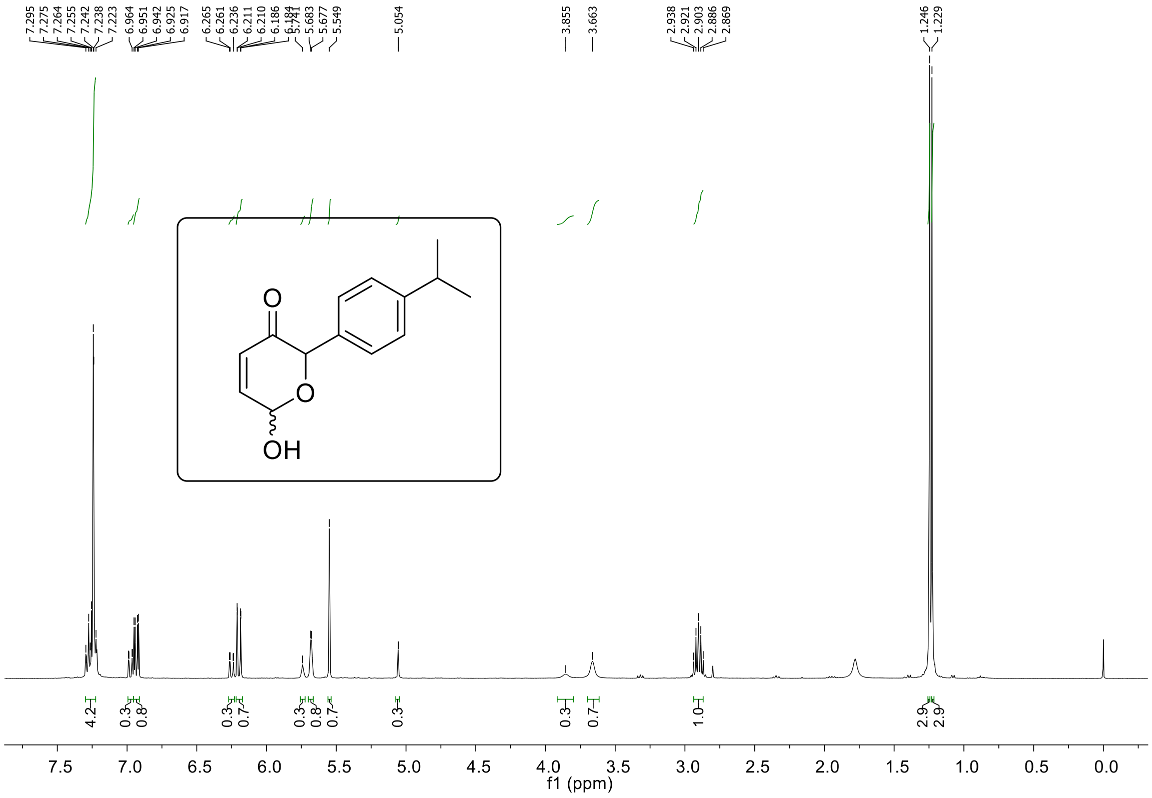

^{1}H NMR (400 MHz, $CDCl_3$) spectrum of 6-hydroxy-2-(4-isopropylphenyl)-2*H*-pyran-3(6*H*)-one (**3g**)

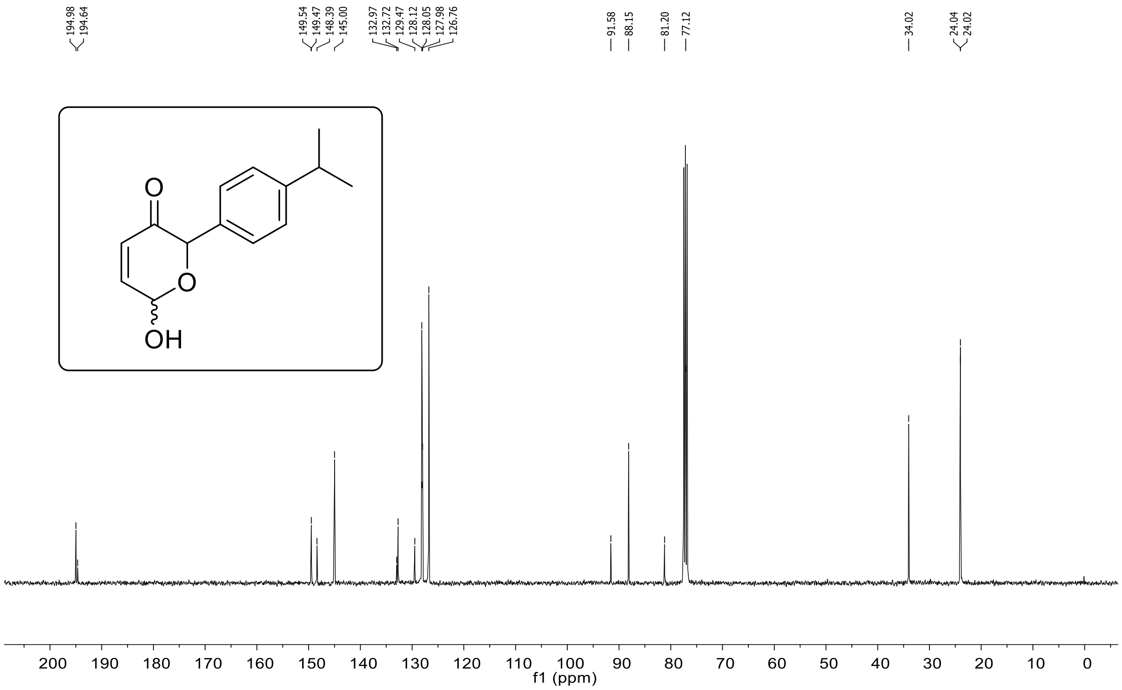

^{13}C NMR (100 MHz, $CDCl_3$) spectrum of 6-hydroxy-2-(4-isopropylphenyl)-2*H*-pyran-3(6*H*)-one (**3g**)

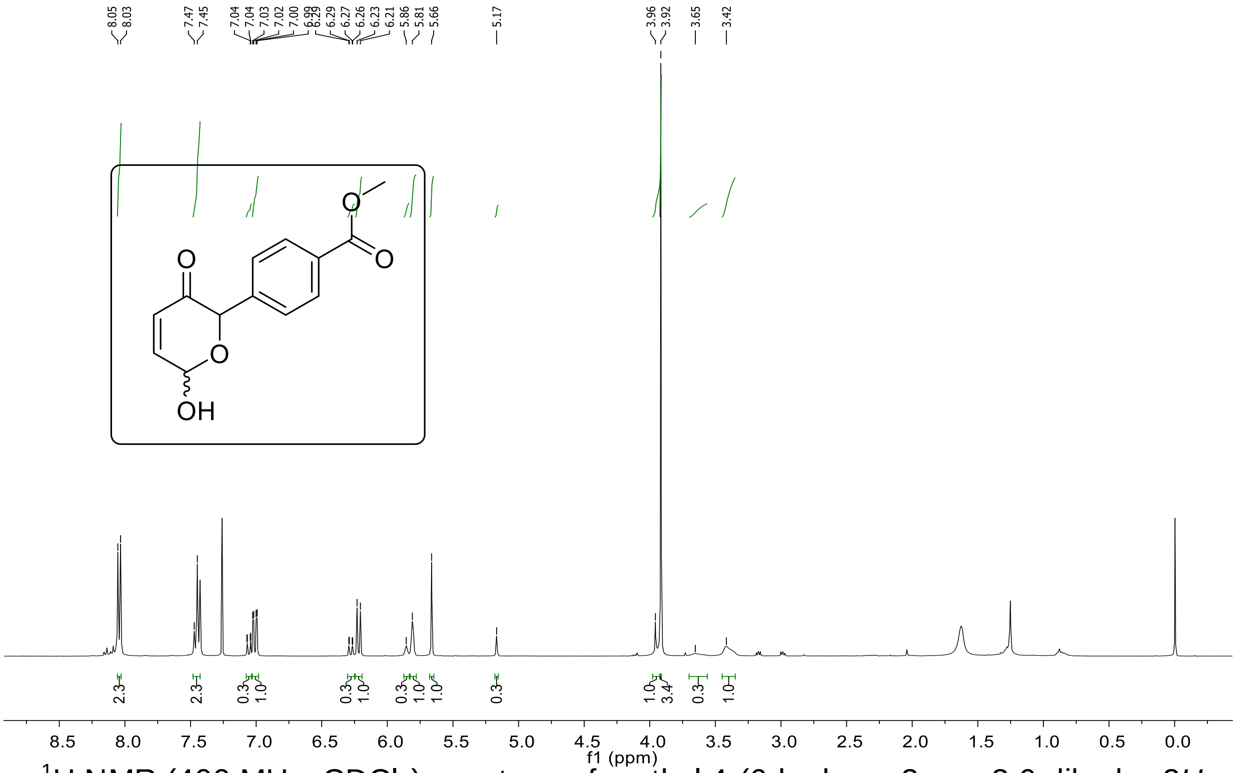

^{1}H NMR (400 MHz, $CDCl_3$) spectrum of methyl 4-(6-hydroxy-3-oxo-3,6-dihydro-2*H*-pyran-2-yl)benzoate (**3h**)

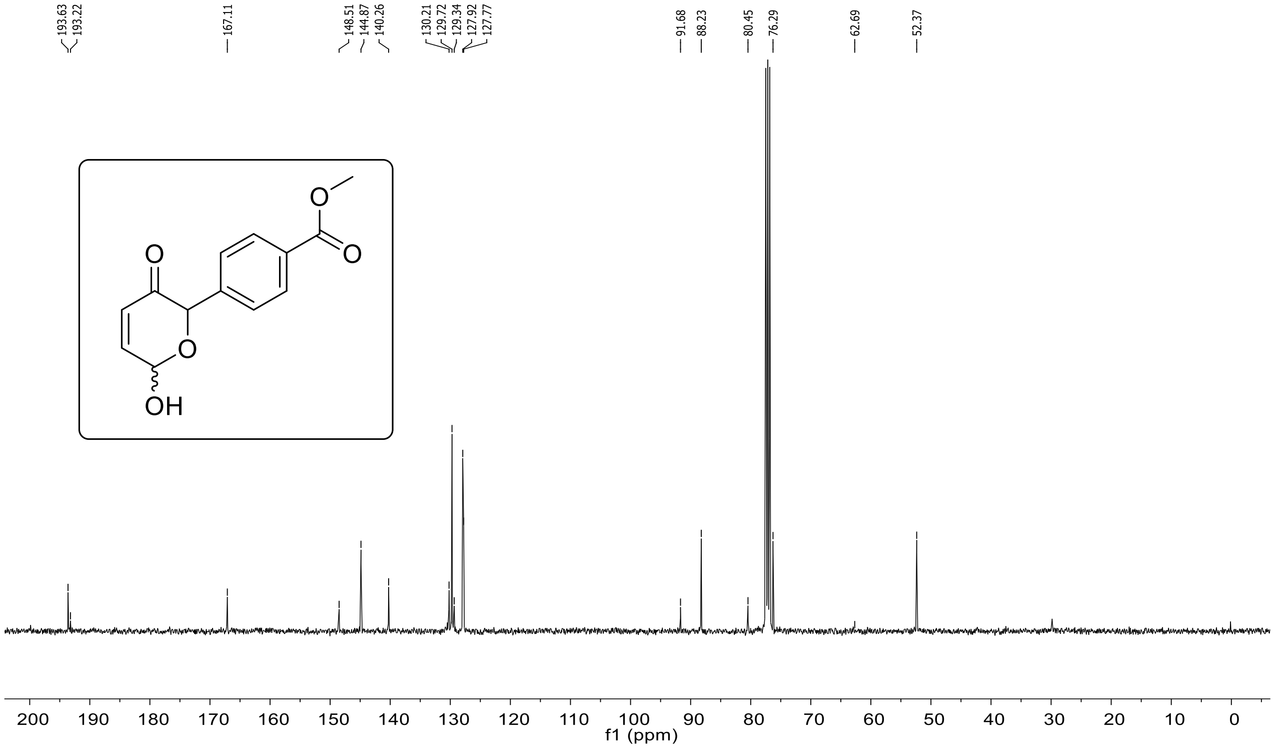

^{13}C NMR (100 MHz, $CDCl_3$) spectrum of methyl 4-(6-hydroxy-3-oxo-3,6-dihydro-2*H*-pyran-2-yl)benzoate (**3h**)

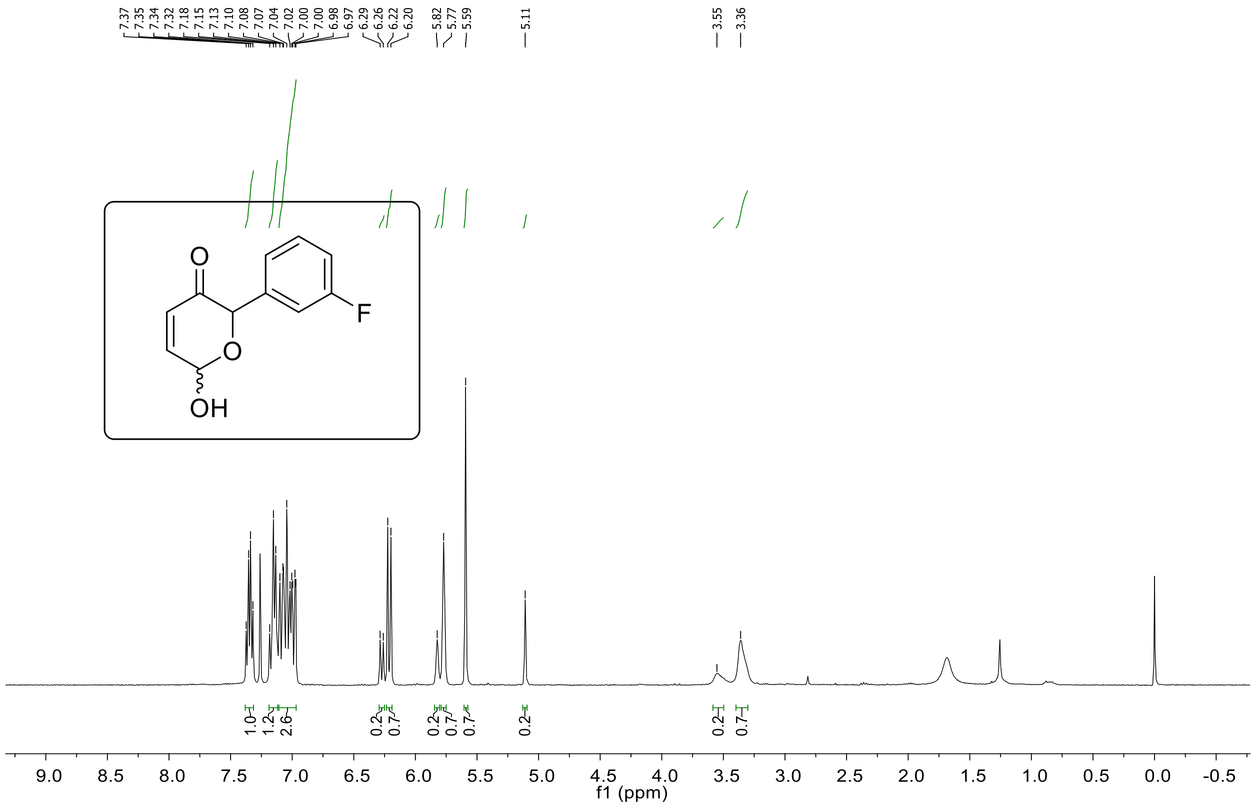

^{1}H NMR (400 MHz, $CDCl_3$) spectrum of 2-(3-fluorophenyl)-6-hydroxy-2*H*-pyran-3(6*H*)-one (**3i**)

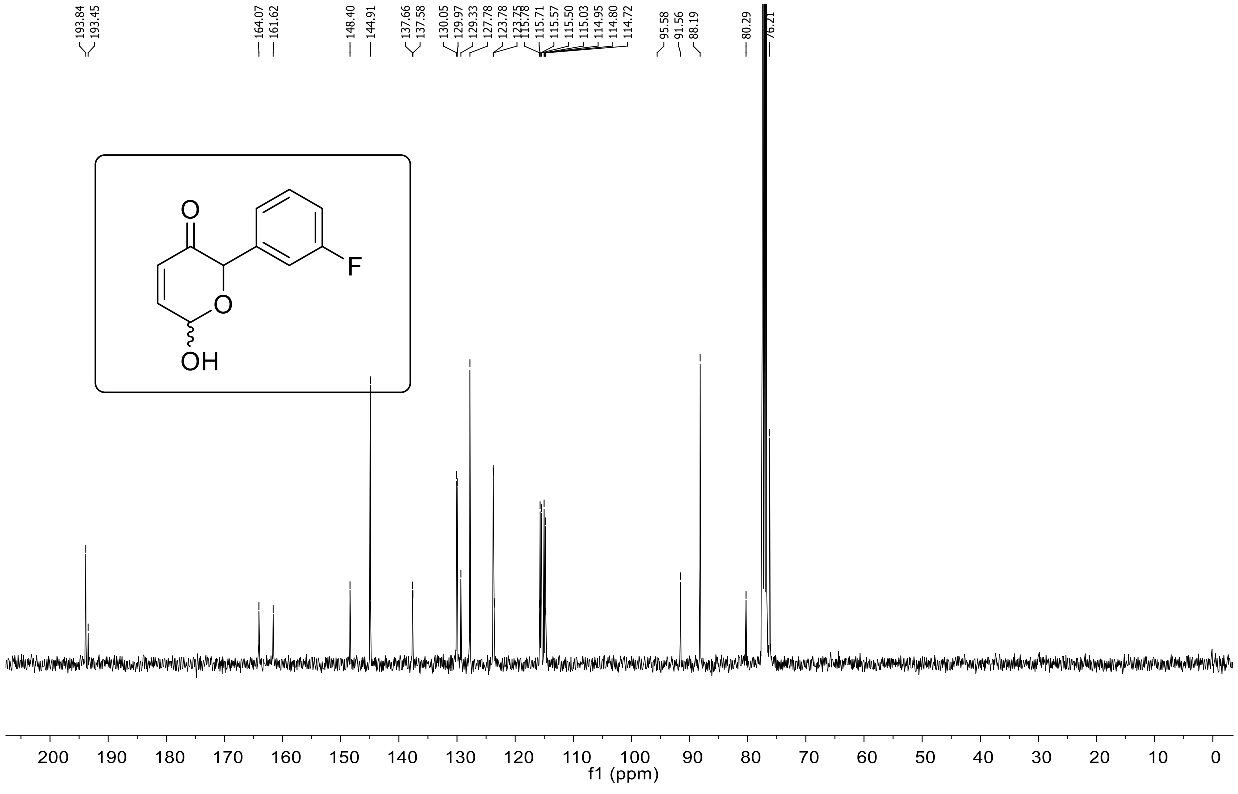

^{13}C NMR (100 MHz, $CDCl_3$) spectrum of 2-(3-fluorophenyl)-6-hydroxy-2*H*-pyran-3(6*H*)-one (**3i**)

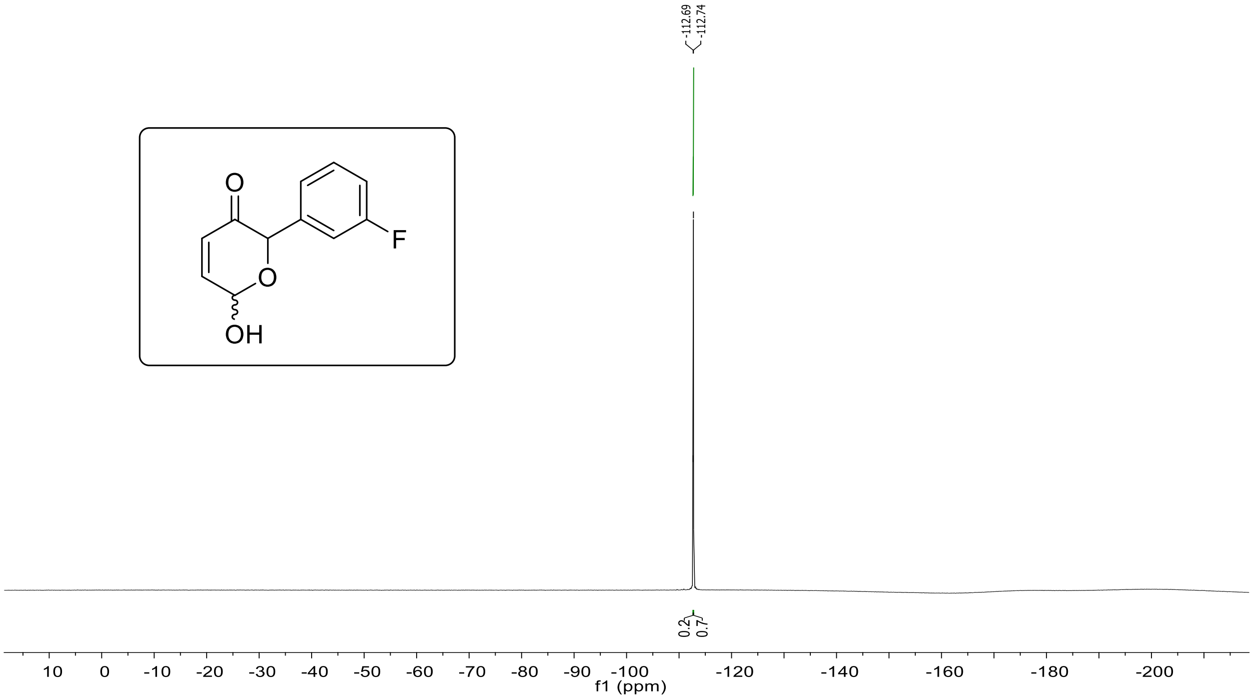

^{19}F NMR (376 MHz, $CDCl_3$) spectrum of 2-(3-fluorophenyl)-6-hydroxy-2*H*-pyran-3(6*H*)-one (**3i**)

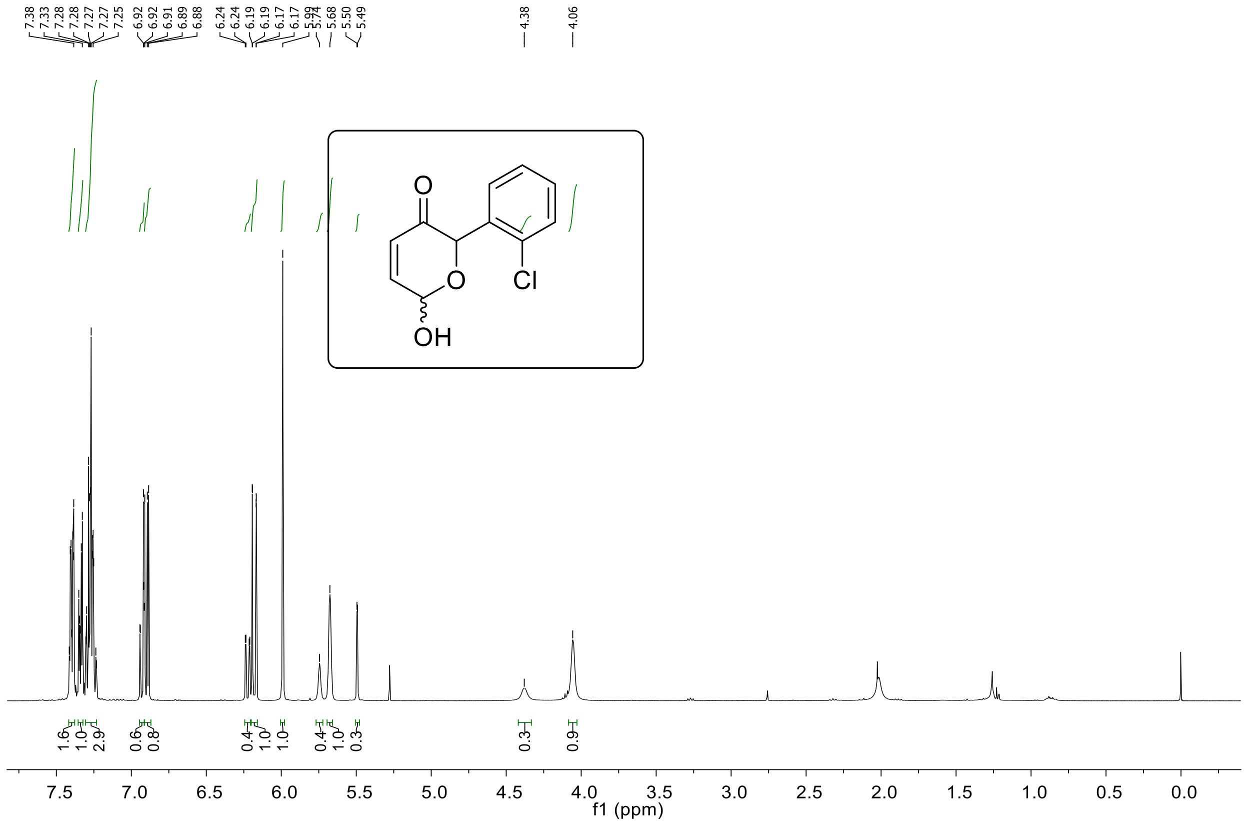

^{1}H NMR (400 MHz, $CDCl_3$) spectrum of 2-(2-chlorophenyl)-6-hydroxy-2*H*-pyran-3(6*H*)-one (**3j**)

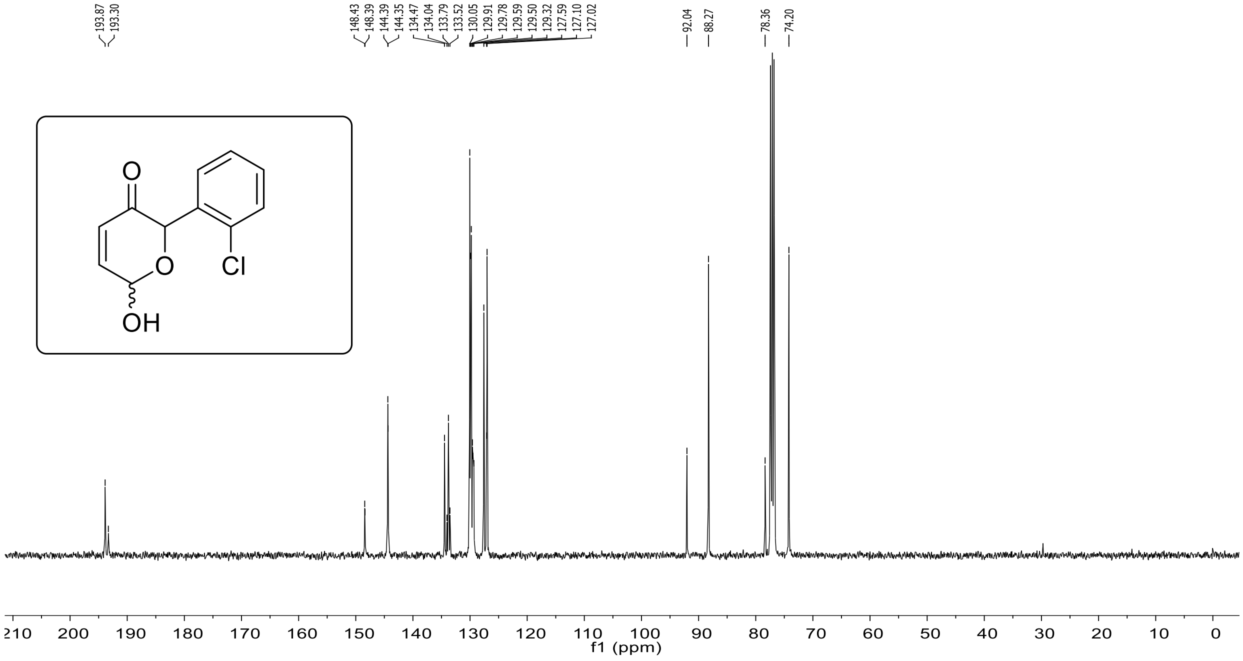

^{13}C NMR (100 MHz, $CDCl_3$) spectrum of 2-(2-chlorophenyl)-6-hydroxy-2*H*-pyran-3(6*H*)-one (**3j**)

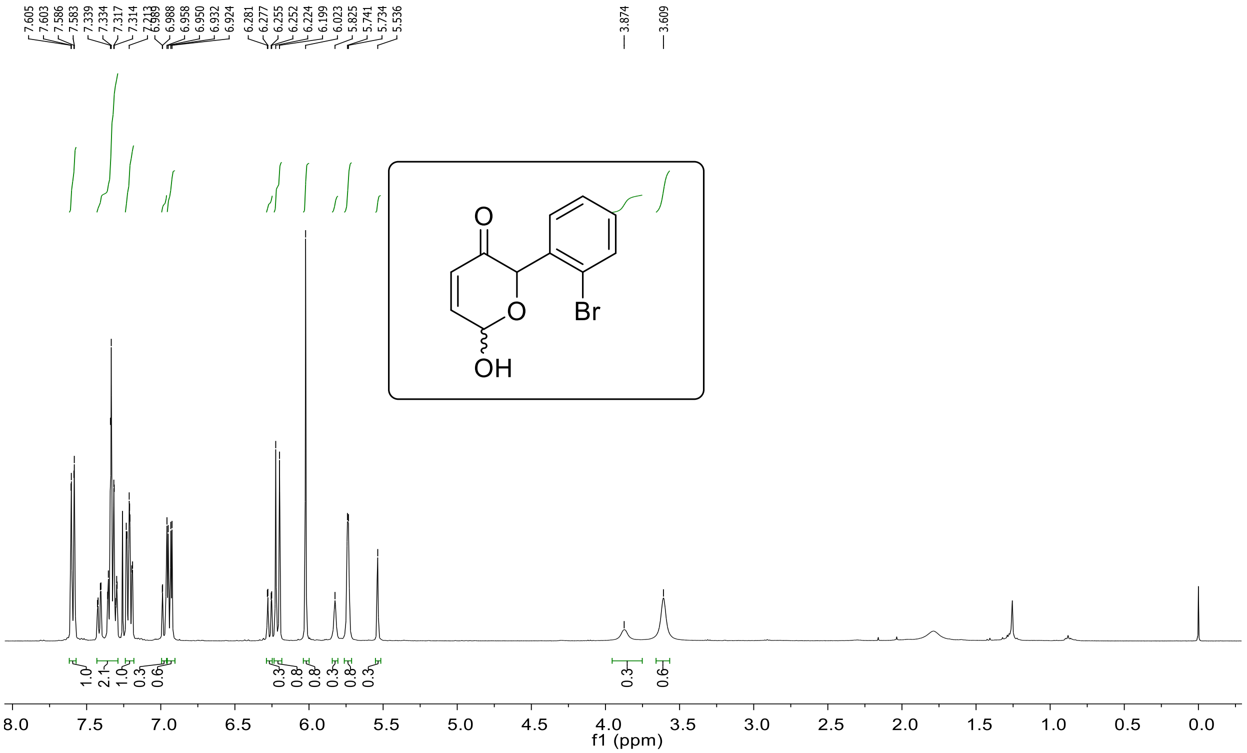

^{1}H NMR (400 MHz, $CDCl_3$ spectrum) of 2-(2-bromophenyl)-6-hydroxy-2*H*-pyran-3(6*H*)-one (**3k**)

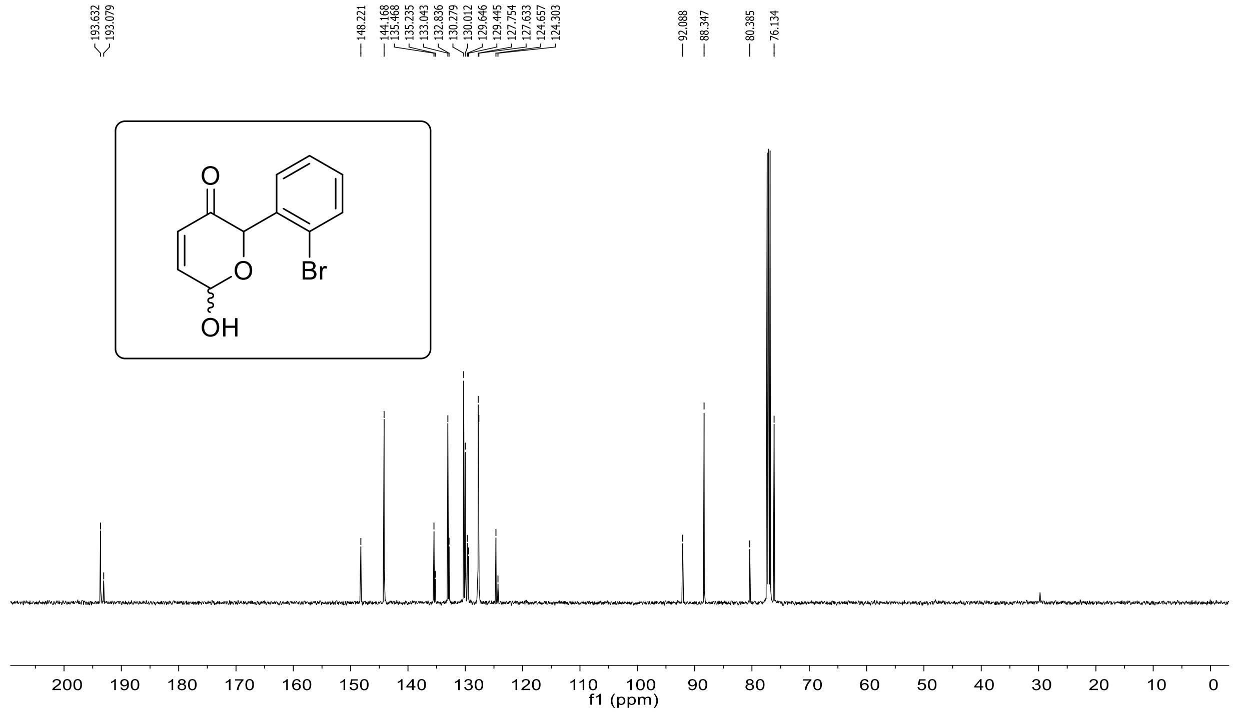

^{13}C NMR (126 MHz, $CDCl_3$) spectrum of 2-(2-bromophenyl)-6-hydroxy-2*H*-pyran-3(6*H*)-one (**3k**)

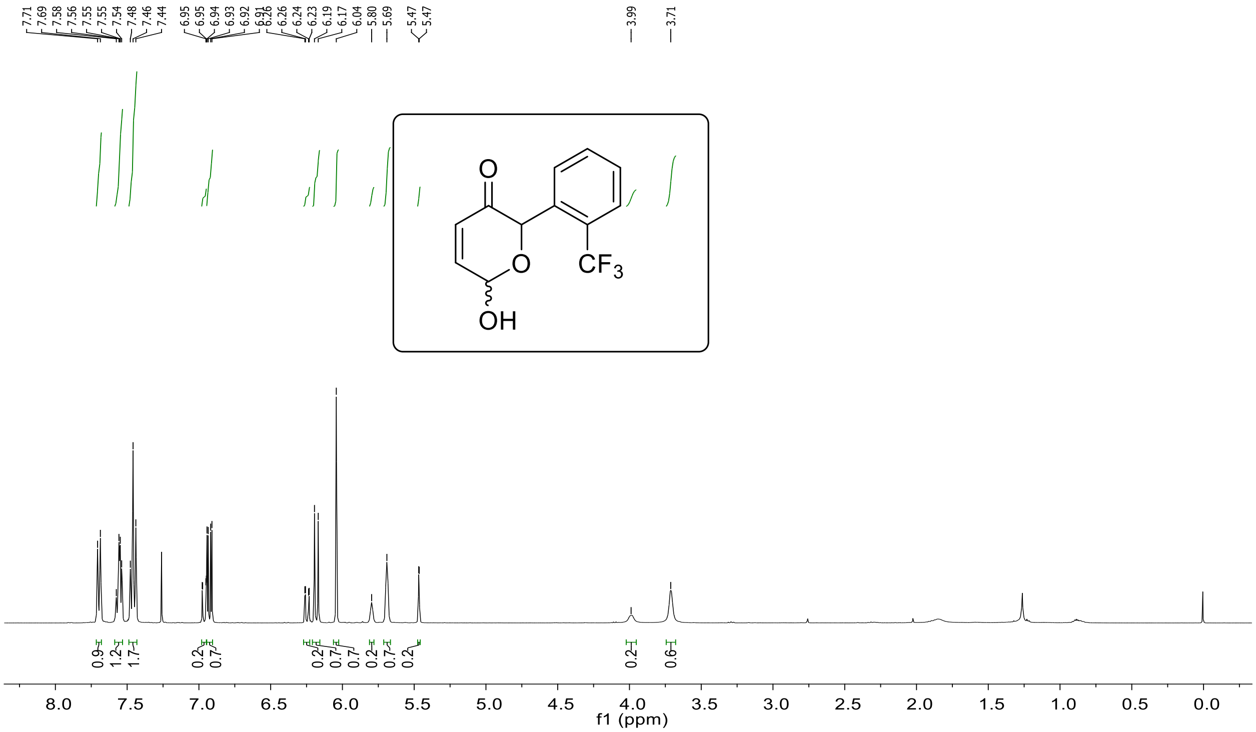

^{1}H NMR (400 MHz, $CDCl_3$) spectrum of 2-(3-fluorophenyl)-6-hydroxy-2*H*-pyran-3(6*H*)-one(**3l**)

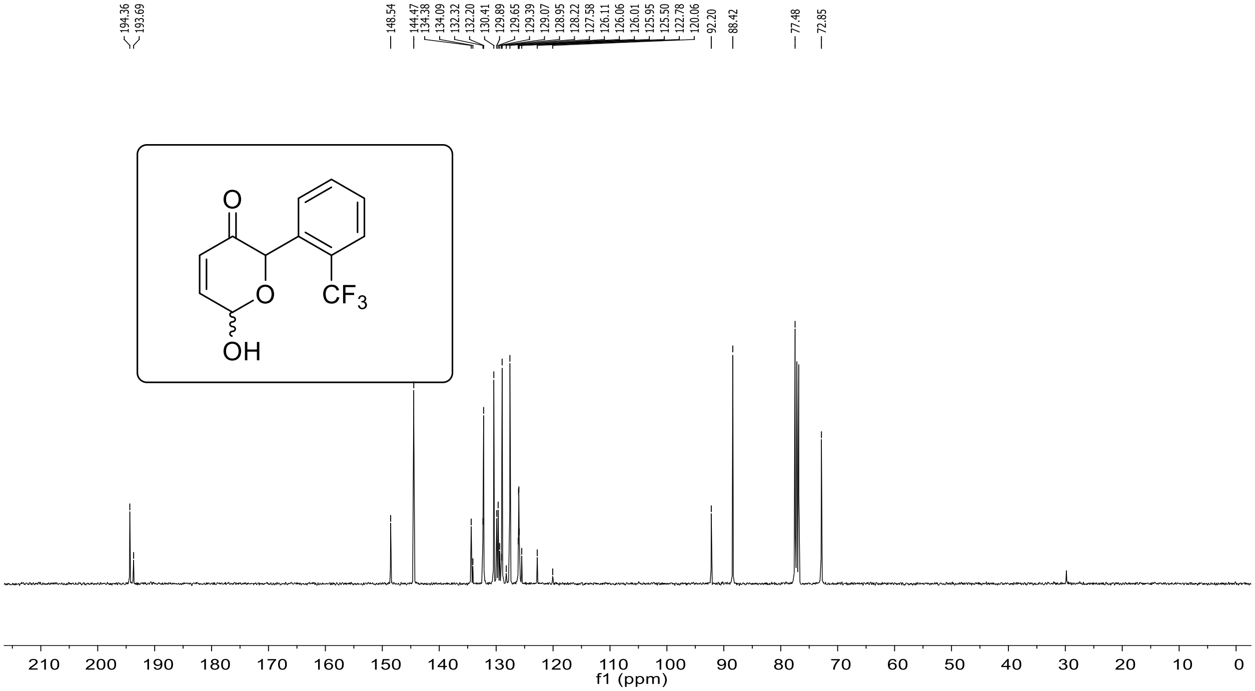

^{13}C NMR (100 MHz, $CDCl_3$) spectrum of 6-hydroxy-2-(2-(trifluoromethyl)phenyl)-2*H*-pyran-3(6*H*)-one (**3l**)

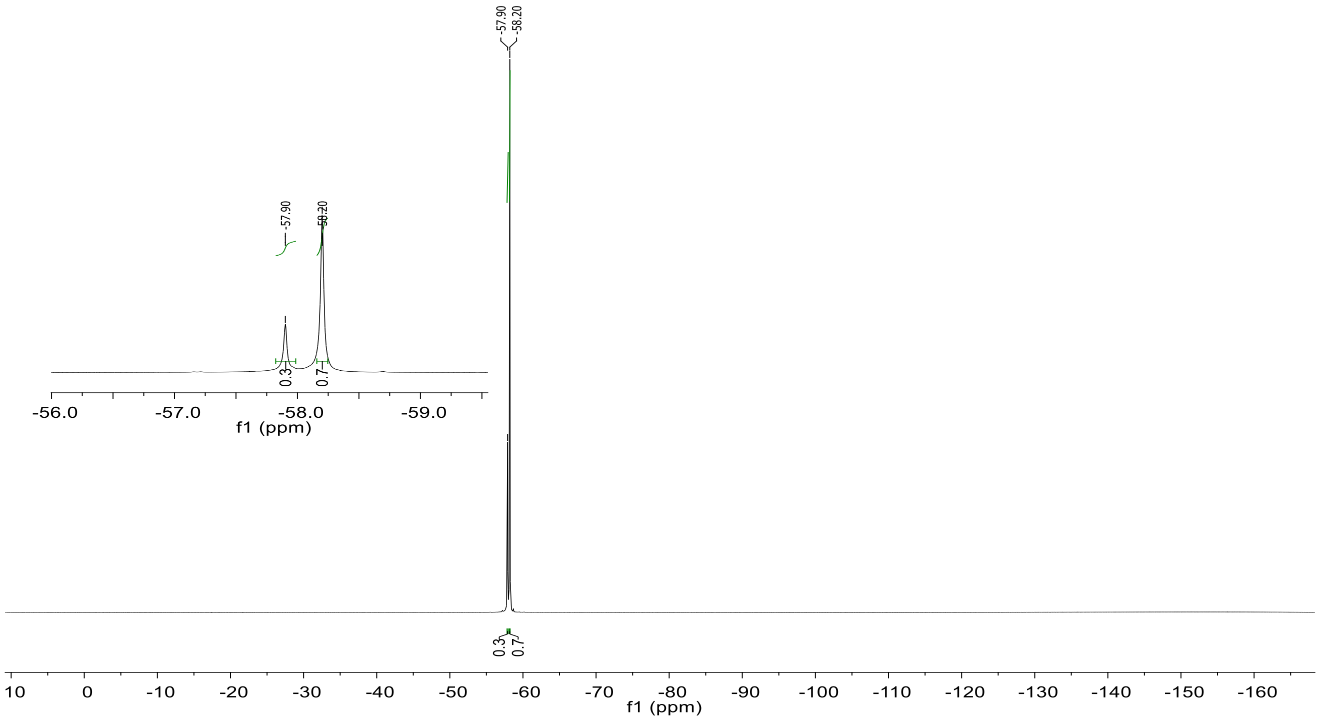

Fluorine spectrum of 6-hydroxy-2-(2-(trifluoromethyl)phenyl)-2*H*-pyran-3(6*H*)-one (**3l)**

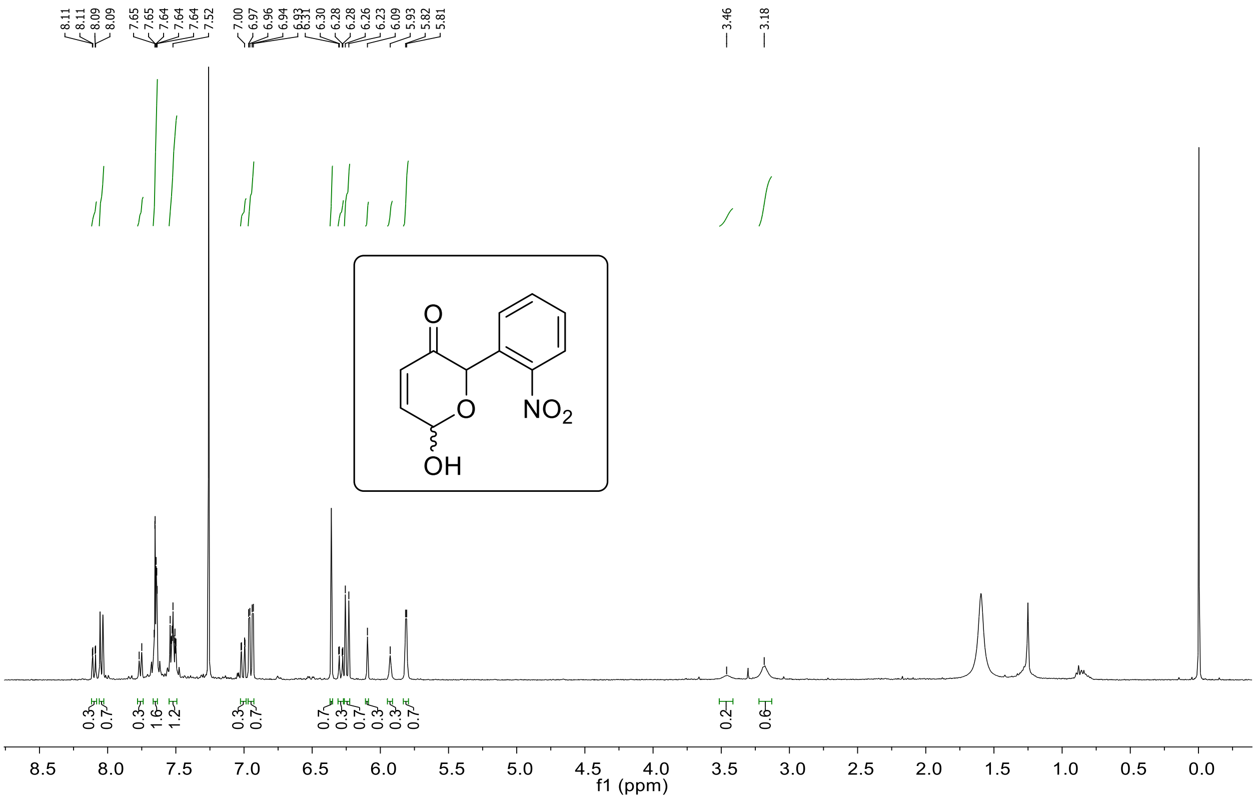

^{1}H NMR (400 MHz, CDCl3) spectrum of 6-hydroxy-2-(2-nitrophenyl)-2*H*-pyran-3(6*H*)-one (**3m**)

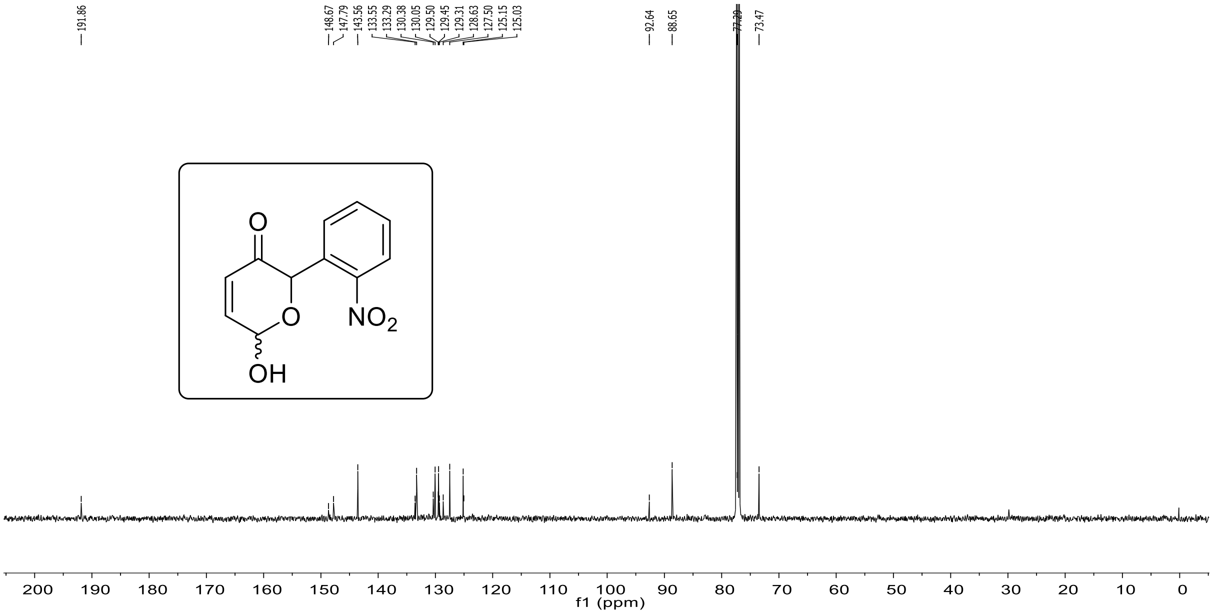

^{13}C NMR (126 MHz, CDCl3) spectrum of 6-hydroxy-2-(2-nitrophenyl)-2*H*-pyran-3(6*H*)-one (**3m**)

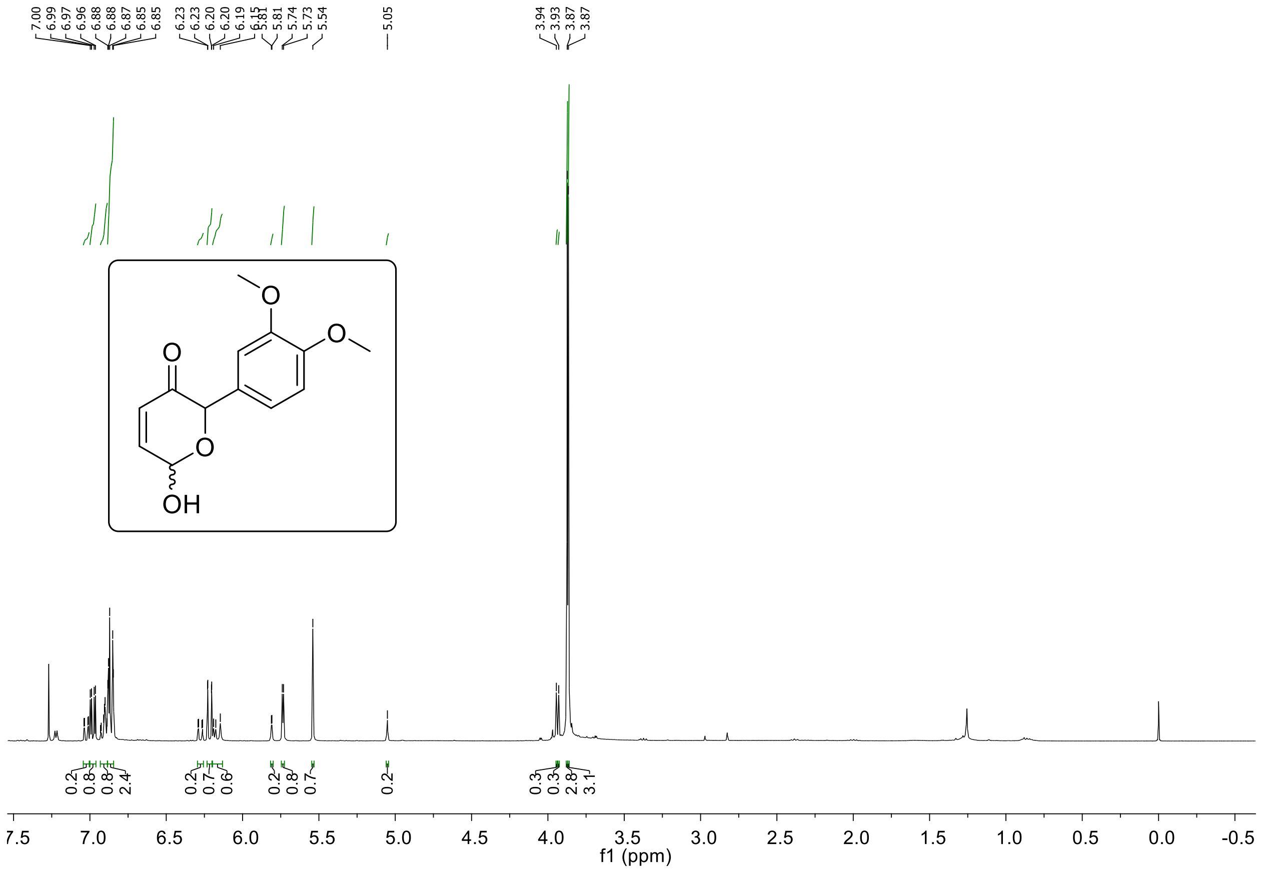

^{1}H NMR (400 MHz, $CDCl_3$) spectrum of 2-(3,4-dimethoxyphenyl)-6-hydroxy-2*H*-pyran-3(6*H*)-one (**3n**)

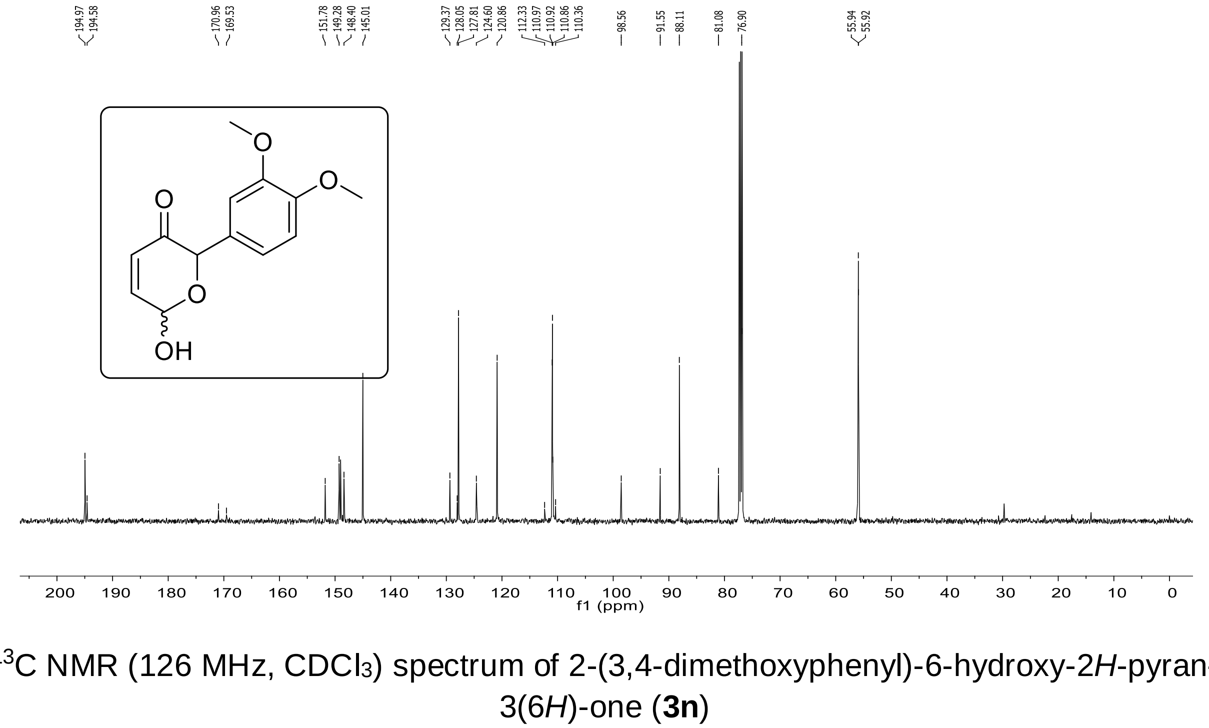

^{13}C NMR (126 MHz, $CDCl_3$) spectrum of 2-(3,4-dimethoxyphenyl)-6-hydroxy-2*H*-pyran-3(6*H*)-one (**3n**)

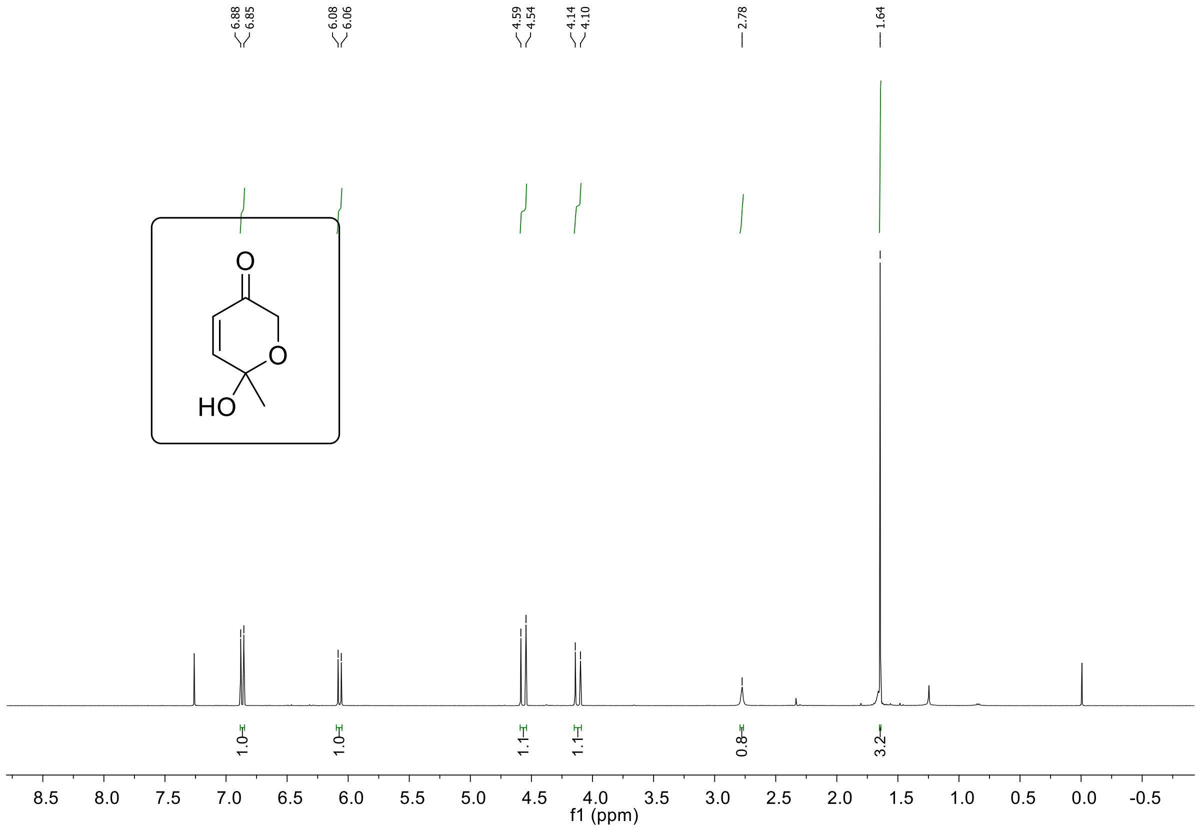

^{1}H NMR (400 MHz, $CDCl_3$) spectrum of 6-hydroxy-6-methyl-2*H*-pyran-3(6*H*)-one (**3o**)

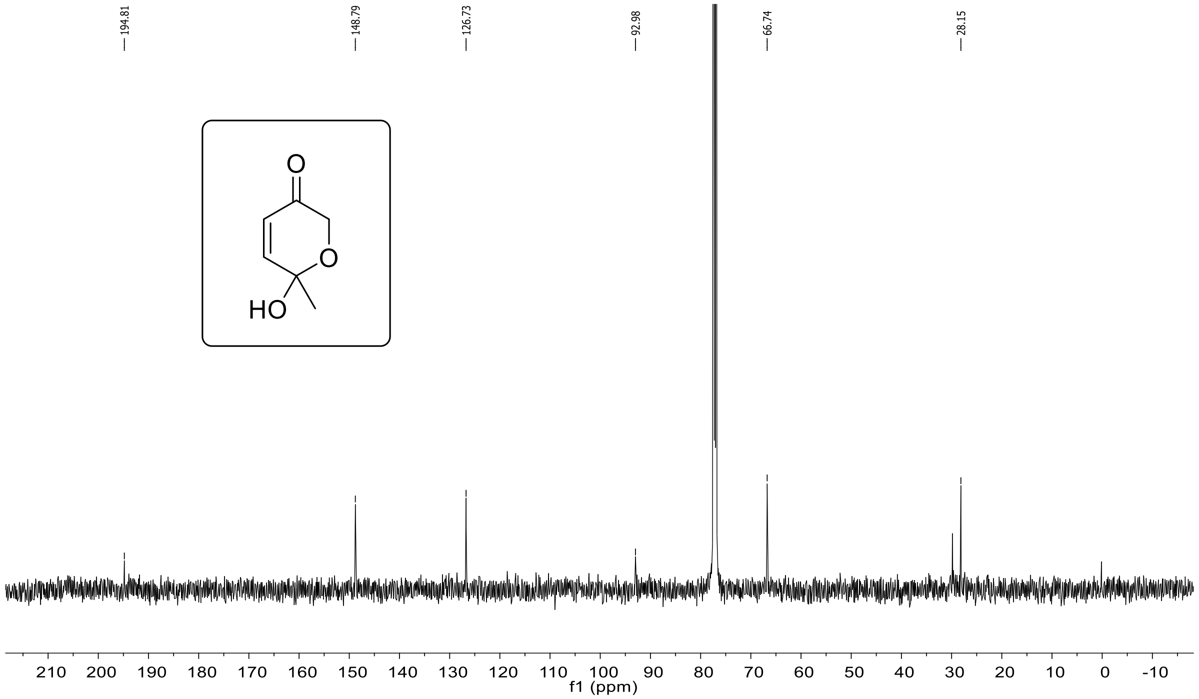

^{13}C NMR (126 MHz, $CDCl_3$) spectrum of 6-hydroxy-6-methyl-2*H*-pyran-3(6*H*)-one **(3o)**

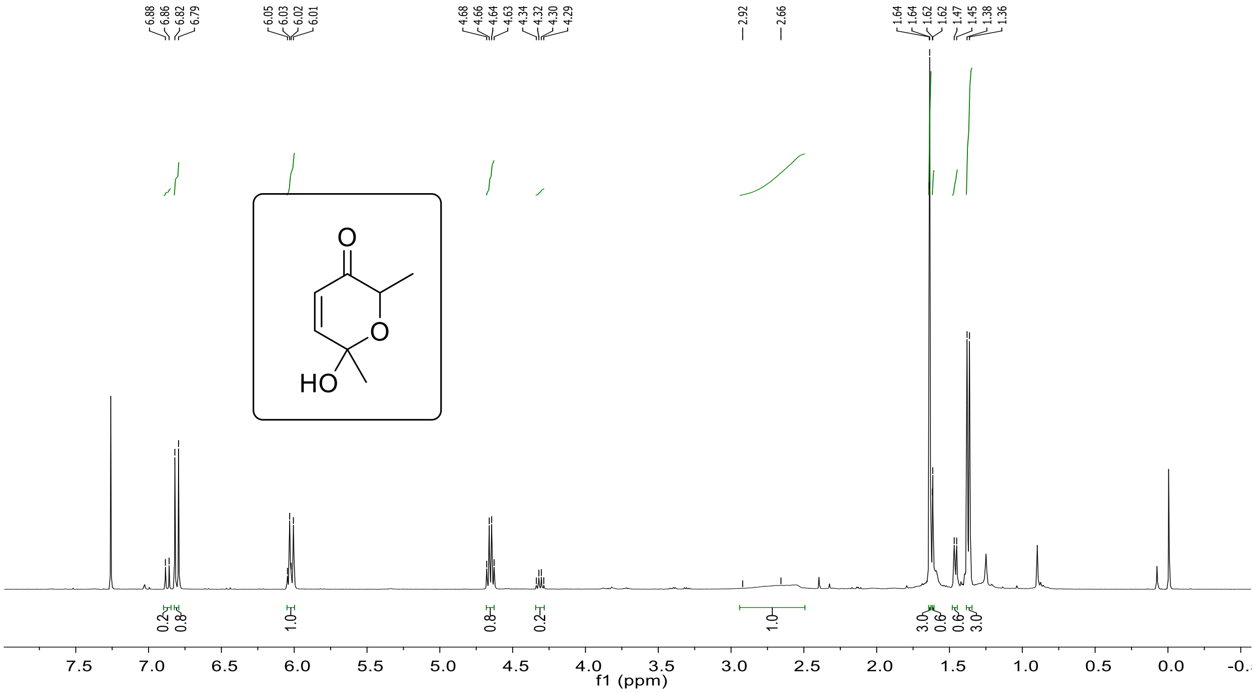

^{1}H NMR (400 MHz, CDCl3) spectrum of 6-hydroxy-6-methyl-2*H*-pyran-3(6*H*)-one **(3p)**

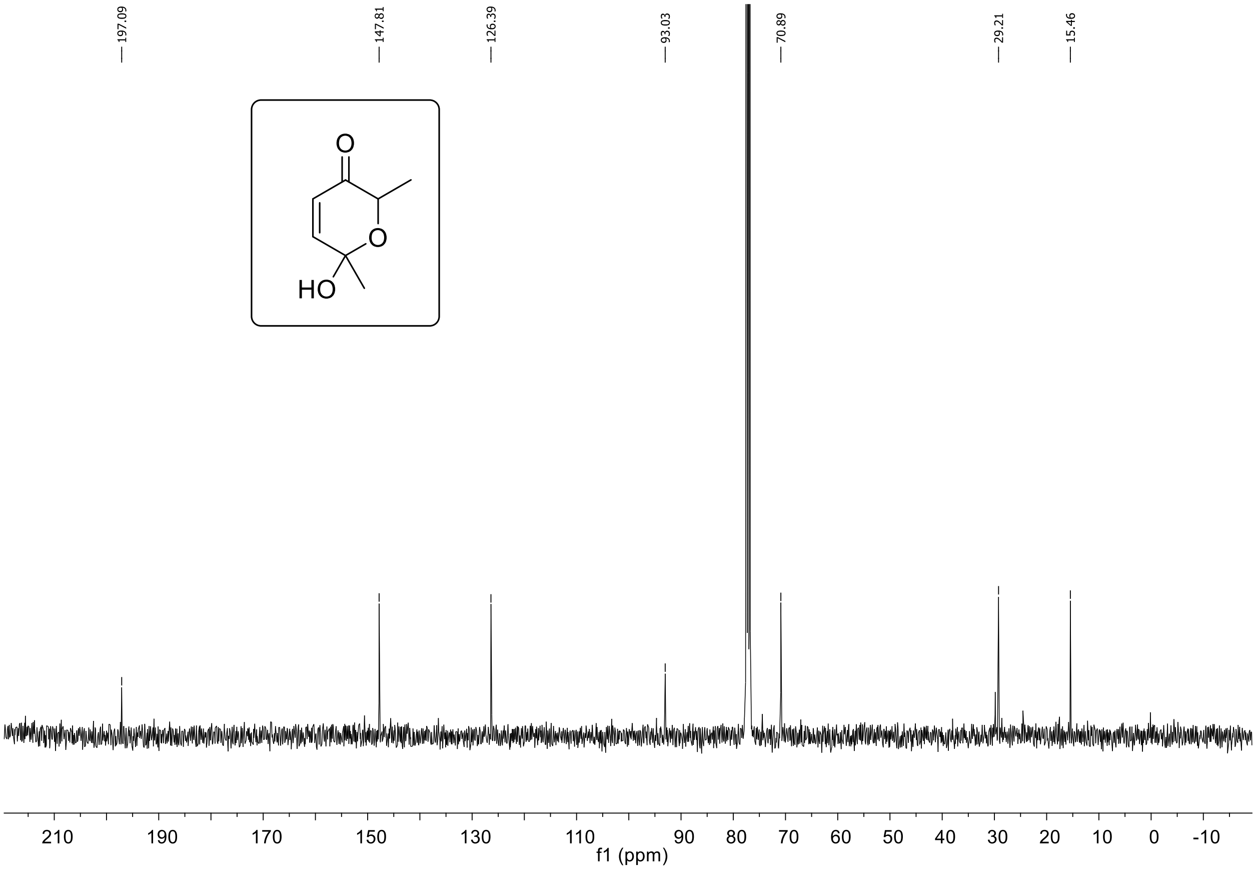

^{13}C NMR (100 MHz, CDCl3) spectrum of 6-hydroxy-6-methyl-2*H*-pyran-3(6*H*)-one (**3p**)
